# Hydroxy- and Hydro-Perfluoroalkylation of Styrenes by Controlling the Quenching Cycle of Eosin Y

**DOI:** 10.3390/molecules28227577

**Published:** 2023-11-14

**Authors:** Haruko Shibata, Moeko Nakayama, Koto Tagami, Tadashi Kanbara, Tomoko Yajima

**Affiliations:** Department of Chemistry, Ochanomizu University, 2-1-1 Otsuka, Bukyo-ku, Tokyo 104-8610, Japan

**Keywords:** perfluoroalkylation, photoredox catalyst, styrene, eosin Y

## Abstract

Fluoroalkyl compounds are widely used, underscoring a pressing need for the development of methods for their synthesis. However, reports on perfluoroalkylation to styrenes have been sparse. In this study, both hydroxy- and hydro-perfluoroalkylation of styrene were achieved using visible light reactions, catalyzed by eosin Y, by selecting appropriate additives and controlling the eosin Y quenching cycle. These reactions are heavy-metal free, use water as the hydroxyl or hydrogen source, and employ inexpensive and readily available reagents.

## 1. Introduction

Fluorine-containing organic compounds play a crucial role in agricultural and pharmaceutical products [1,2,3,4,5,6] and the development of functional materials [7,8]. However, very few fluorine-containing organic compounds exist naturally, and they must be synthesized artificially. The incorporation of perfluoroalkyl groups using perfluoroalkyl halides into organic compounds is an effective and widely used method for the synthesis of fluorine-containing compounds [9,10,11,12]. It is achieved using perfluoroalkyl halides, which are upstream raw materials in the fluorine industry, offering a diverse array of fluoroalkyl groups. Perfluoroalkyl halides possess low homolysis energies and redox potentials, causing them to readily form perfluoro radicals. Indeed, many examples of perfluoroalkylation to electron-rich olefins have been reported. However, few studies have reported on perfluoroalkylation with conjugated olefins [13], particularly styrene, due to the likelihood of side reactions such as oligomerization, polymerization, and nucleophilic trapping.

**Scheme 1:**
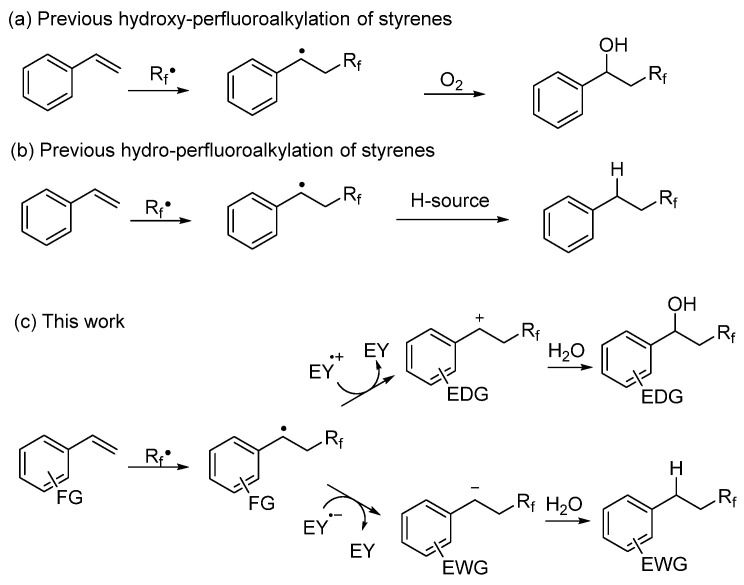
Hydroxy- and hydro-perfluoroalkylation of styrenes using perfluroalkyl halide as a fluorine source.

Hydroxy- [14,15,16,17,18,19,20,21,22,23,24,25] and hydro-perfluoroalkylation [26,27,28,29,30,31,32,33] have been reported as examples of perfluoroalkylation to styrene. Hydro-perfluoroalkylation was reported in 1999 using light irradiation with a metal-halogen lamp in the presence of (Bu_3_Sn)_2_ [14]. In 2018, visible-light-induced hydroxy-perfluoroalkylation by Jiao et al. was realized using ruthenium complexes as a photoredox catalyst [17]. Chen et al. [19] and our group [24,25] have reported visible-light-induced hydroxy-perfluoroalkylation. During such reactions, catalyzation is achieved by amines or enamines and the process is free of heavy metals. Oxygen molecules trap the intermediate benzyl radicals (Scheme 1a). For hydro-perfluoroalkylation, a reaction using a radical initiator was reported in 2010 by Postigo et al. In this type of reaction, the benzyl radicals produced are hydrogen-trapped by tris(trimethylsilyl)silane [26]. Noël et al. reported a visible light reaction in which an iridium-catalyzed reaction used 4-hydroxythiophen as a hydrogen donor [29] (Scheme 1b).

Here, we have developed a series of eosin-Y-catalyzed [34,35] visible-light-induced fluoroalkylations using perfluoroalkyl halides [36,37,38]. Eosin Y is an inexpensive, safe, and readily available organic dye. The reactions proceed by both reductive and oxidative quenching cycles. We have reported the iodoperfluoroalkylation of olefins by oxidative formation of perfluoroalkyl radicals with the addition of sodium thiosulfate [36], and their hydro-perfluoroalkylation, under reductive conditions, in the presence of amines [37]. We hypothesized that if this reaction were applied to styrene in the presence of water, hydroxy-perfluoroalkyl products would be obtained via benzyl cation intermediates under oxidative reaction conditions, and hydro-perfluoroalkylated products would be produced via benzyl anion intermediates under reductive reaction conditions (Scheme 1c). Such reactions via benzyl anions and cations have only been reported using trifluoromethylation reagents, and we have found no reports using perfluoroalkyl halides.

## 2. Results and Discussion

Based on our previous reaction conditions, perfluoroalkylations to styrenes were attempted (Table 1). Following our previously reported methodology on iodoperfluoroalkylation [36], we proceeded with the oxidative quenching cycle of eosin Y. Firstly, we reacted *p*-methoxystyrene with perfluorohexyl iodide (1.2 equiv.). This reaction uses eosin Y (10 mol%) as the catalyst in the presence of aqueous Na_2_S_2_O_3_. It requires photoirradiation for 3 h using a 12 W white light-emitting diode (LED) lamp. As expected, it yielded a 40% hydroxy-perfluorohexylated product (Entry 1). The addition of potassium carbonate to trap the byproduct HI improved the yield (Entry 2). When the amount of Na_2_S_2_O_3_ was reduced to 0.25 eq., the yield was further improved (Entry 3). However, the yield decreased when the reaction was performed without Na_2_S_2_O_3_ (Entry 4). The yield was not improved using 3 eq. of perfluorohexyl bromide (Entry 5). According to our previously reported hydro-perfluoroalkylation methodology [38], the reaction proceeded via the reductive quenching cycle of eosin Y (Entry 6). To test this, *p*-cyanostylene was reacted with perfluorohexyl iodide (3.0 equiv.) in the presence of diisopropylethylamine (DIPEA, 2.0 equiv.). As a result, a 70% hydro-perfluoroalkylated product was obtained as the sole product. When perfluoroalkyl bromide was used under reaction conditions in the presence of sodium thiosulfate, or conversely when perfluoroalkyl iodide was used in the presence of amine, no product was obtained (Scheme S1).

**Scheme 2:**
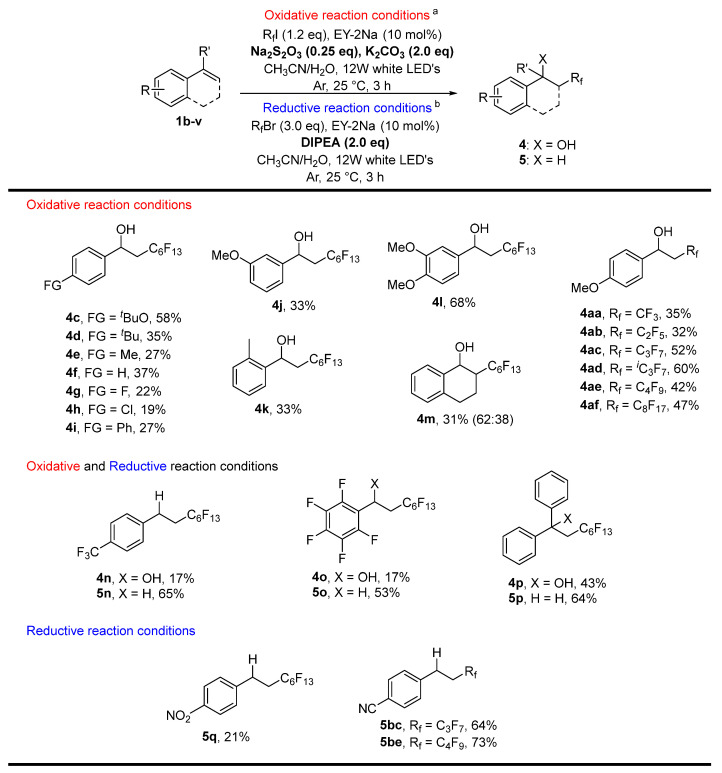
Hydroxy- and hydro-perfluoroalkylation of various styrenes. ^a^ Oxidative reaction conditions: Eosin Y (10 mol%), **1** (0.25 mmol), **2**, Na_2_S_2_O_3_ (0.25 equiv.), and K_2_CO_3_ (2.0 equiv.) in CH_3_CN/H_2_O (7.5 mL/1 mL) under an argon atmosphere with 3 h irradiation with white LED light at 25 °C. ^b^ Oxidative reaction conditions: Eosin Y (10 mol%), **1** (0.25 mmol), **3** and DIPEA (2.0 equiv.) in CH_3_CN/H_2_O (7.5 mL/1 mL) under an argon atmosphere with 3 h irradiation with white LED light at 25 °C.

Subsequently, reactions using oxidative (Table 1, Entry 3) or reductive (Table 1, Entry 6) quenching cycles were applied to substituted styrenes (Scheme 2). First, we tried oxidative reaction conditions using various *p*-substituted styrenes (**1c**–**i**). Styrene with a tert-butoxy group (**1c**) gave good yields of hydroxy-perfluorohexylated product (**4c**). The yields were lowered with tert-butyl (**1d**) and methyl (**1d**) substituents or unsubstituted styrene (**1f**); even lower yields were obtained for halogen-substituted styrene (**1g**, **h**). The reaction of m-methoxy (**1j**) and o-methyl (**1k**) styrene gave 33% yields whereas o,p-dimethoxystyrene (**1l**) improved the yield to 68%. The hydroxy-perfluorohexylation proceeded with 1,2-dihydronaphthalene (**1m**), giving a 31% yield with a 62:38 diastereomer ratio. Reactions with different perfluoroalkyl iodides were also investigated. The reaction with gaseous CF_3_I yielded a 35% hydroxy-trifluoromethylated product (**4aa**). Perfluoroethyl, propyl, *i*-propyl, and butyl octyl all yielded the corresponding hydro-perfluoroalkyalted products (**4ab**–**f**).

Next, *p*-(trifluorometyl)stylene (**1n**), 2,3,4,5,6-pentafluorostylene (**1o**), and 1,1-diphenylethylene (**1p**) were tested, indicating that reactions proceeded under both oxidative and reductive conditions for these substrates. In particular, 1,1-diphenylethylene (**1p**) produced good yields of hydroxy- (**4p**) and hydro-perfluoroalkylated product (**5p**). In contrast, previous radical perfluoroalkylation of **1p** yielded olefination products [39,40]. With *p*-nitrostyrene (**1q**), the yield was only 21%. With styrene, the yield was also 21%, but this may be due to the radical inhibitory effect of the nitro compound. The introduction of perfluoropropyl and butyl was investigated using p-cyanostylene (**1b**)**,** producing high yields of the products **5bc** and **5be**. The effect of substituents in para-substituted styrene is correlated with the Hammett constant [41,42]. Hydroxy-perfluoroalkylation (**4**) was dominant with electron-donating substituents, which have a smaller Hamett σ parameter. In contrast, hydro-perfluoroalkylation (**4**) was dominant with electron-withdrawing substituents (Table S1).

**Scheme 3:**
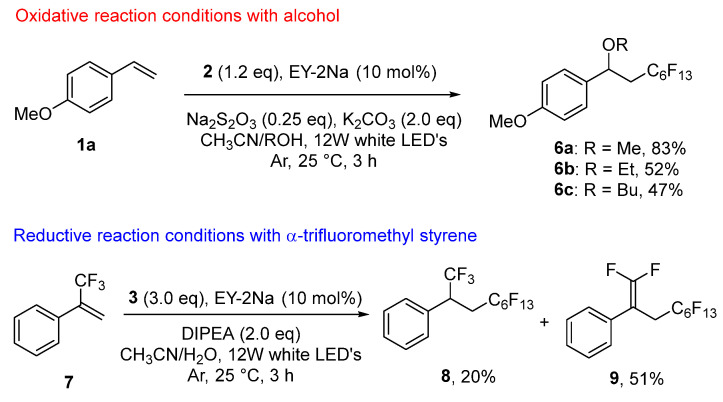
Types of perfluoroalkylation.

We attempted reactions utilizing the respective properties of benzyl cations and anions (Scheme 3). Under oxidative reaction conditions via benzyl cations, alkoxyperfluoroalkylation was investigated by the addition of alcohols instead of water. Adding methanol, ethanol, or butanol produced corresponding methoxy (**6a**), ethoxy (**6b**), and butoxy (**6c**) products. Under reductive reaction conditions via benzyl anions, the synthesis of fluoro-olefins using α-trifluoromethylstyrene (**7**) as a substrate was performed [43,44,45]. The reaction produced a 51% yield of fluoro-olefins (**9**) along with 20% of hydro-perfluoroalkylated product (**8**).

**Scheme 4:**
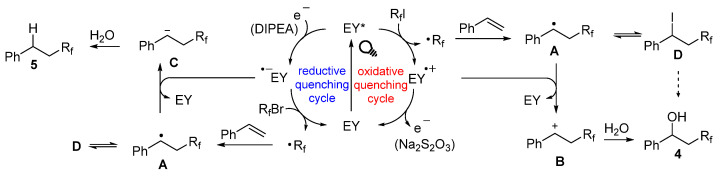
Proposed mechanism for hydroxy- and hydro-perfluoroalkylation.

Based on our experimental results and previous reports [36,37,38,46], we propose the following reaction mechanism (Scheme 4). Under oxidative reaction conditions, photo-irradiated eosin Y (EY*) is reacted with perfluoroalkyl iodide to give a perfluoroalkyl radical. This radical is added to styrene to give intermediate radical **A**. Radical **A** is converted to benzyl cation **B** via the radical cation of eosin Y. At this time, the presence of an excess amount of Na_2_S_2_O_3_ would consume the radical cation of eosin Y, thus reducing the yield of the desired product. Then we investigated the equimolar amount of Na_2_S_2_O_3_ and 0.25 equivalents were found to be optimal (Table S2). Then, cation **B** is reacted with water to give the hydroxy-perfluoroalkylated product **4** (Scheme S2). When the time course of the reaction was followed without potassium carbonate, iodide **D** was also observed during the reaction (Table S3). This suggests a possible pathway in which radical intermediate **A** is trapped by iodine once and then reverts back to a radical or is directly hydroxylated. Under a reductive quenching cycle, the radical anion of eosin Y is produced by the reaction of DIPEA and photo-irradiated eosin Y [47,48,49]. The radical anion of eosin Y reduces perfluoroalkyl bromide to produce the perfluoroalkyl radical, which is reacted with styrene. The subsequent reaction with the radical anion of eosin Y gives benzyl anion **C**. In this case, two equivalents of amine are required because the radical anion of eosin Y reacts with both the perfluoroalkyl bromide and the intermediate radical **A**. The benzyl anion **C** reacts with water to produce the hydro-perfluoroalkylated product **5.** This is suggested by the experimental results as the reaction in the absence of water does not yield product **5n**, and the yield is not improved even in the presence of THF, which can act as a hydrogen radical donor (Table S2). The stability of the intermediate benzyl cation (**B**) and anion (**C**) is influenced by the electronic properties of the substituent on styrene, which is linked to the correlation between the product ratio and the Hammett constant.

## 3. Materials and Methods

### 3.1. General Information

All reactions were performed under the argon atmosphere. ^1^H, ^13^C, and ^19^F NMR spectra were recorded on a JEOL GSX-400 spectrometer (Jeol, Akishima, Tokyo, Japan) (400 MHz for ^1^H, 126 MHz for ^13^C, and 376 MHz for ^19^F), a JEOL ECX-500 spectrometer (Jeol, Akishima, Tokyo, Japan) (500 MHz for ^1^H and 471 MHz for ^19^F), or a BRUKER AVANCE 600 spectrometer (Bruker, Billerica, MA, USA) (600 MHz for ^1^H and 151 MHz for ^13^C) with CDCl_3_ as the solvent, tetramethylsilane (TMS: δ 0 ppm for ^1^H), chloroform-d (CDCl_3_: δ 76.9 ppm for ^13^C), and hexafluorobenzene (C_6_F_6_: −162.2 ppm for ^19^F) as an internal standard. IR spectra were taken on SHIMADZU IRSpirit-T, and HRMS were obtained with a JEOL JMS-T100TD (FD) (Jeol, Akishima, Tokyo, Japan), JEOL JMS-700 (EI) (Jeol, Akishima, Tokyo, Japan), or BRUKER Compact (ESI) (Bruker, Billerica, MA, USA). Precoated Merck Kieselgel 60 F254 and Kanto silica gel 60 (spherical neutral) were used for thin-layer chromatography and flash chromatography, respectively. Visualization was accomplished by UV light (254 nm). All commercially available reagents and solvents were used as received without further purification.

### 3.2. General Procedure

#### 3.2.1. Oxidative Reaction Conditions

To a solution of styrene (0.300 mmol) in CH_3_CN/H_2_O (7.50 mL/1.00 mL) mixed solvent were added Eosin Y-2Na (20.7 mg, 0.0299 mmol), Na_2_S_2_O_3_ (11.9 mg, 0.0753 mmol), K_2_CO_3_ (82.9 mg, 0.600 mmol), and perfluoroalkyl iodide (0.360 mmol) under an Ar atmosphere. Then, the mixture was stirred under irradiation with 12 W white LEDs at room temperature for 3 h. Then, the mixture was extracted with dichloromethane, dried over Na_2_SO_4_, filtered, and concentrated in vacuo. The residue was purified by column chromatography on silica gel to yield hydroxy-perfluoroalkylation products as a colorless oil.

#### 3.2.2. Reductive Reaction Conditions

To a solution of styrene (0.200 mmol) in dry CH_3_CN/H_2_O (5.00 mL/1.00 mL) mixed solvent were added Eosin Y-2Na (13.8 mg, 0.020 mmol), DIPEA (0.07 mL, 0.402 mmol), and perfluoroalkyl bromide (0.600 mmol) under an Ar atmosphere. Then, the mixture was stirred under irradiation with 12 W white LEDs at room temperature for 3 h. Then, the mixture was extracted with dichloromethane, dried over Na_2_SO_4_, filtered, and concentrated in vacuo. The residue was purified by column chromatography on silica gel to yield hydro-perfluoroalkylation products as a colorless oil.

### 3.3. Characterization Data of Products

#### 3.3.1. 3,3,4,4,5,5,6,6,7,7,8,8,8-Tridecafluoro-1-(4-methoxyphenyl)octan-1-ol (**4a**)

The compound was synthesized as described in oxidative reaction conditions. The product was purified by column chromatography (Hexane/EtOAc = 10:1). Yellow oil (70.5 mg, 50%); ^1^H NMR (500 MHz, CDCl_3_) δ 7.31 (2H, d, *J* = 8.5 Hz), 6.91 (2H, d, *J* = 7.0 Hz), 5.16 (1H, dd, *J* = 8.5, 3.5 Hz), 3.81 (3H, s), 2.68–2.56 (1H, m), 2.45–2.33 (1H, m), 2.17 (1H, s); ^13^C NMR (151 MHz, CDCl_3_) δ 159.7, 134.2, 127.1 (2C), 119.7–110.3 (6C, m), 114.3 (2C), 67.7, 55.5, 39.8 (t, *J* = 21.0 Hz), 32.7, 18.3, 14.0; ^19^F NMR (471 MHz, CDCl_3_) δ −81.3 (3F, s), −113.0 (1F, d, *J* = 282.3 Hz), −114.4 (1F, d, *J* = 282.3 Hz), −122.3 (2F, s), −123.4 (2F, s), −124.2 (2F, s), −126.7 (2F, s); IR; 3431,3007, 2960, 2914, 1614, 1515, 1232, 1182, 1144, 1036, 812, 707; HRMS (EI^+^) calcd for C_15_H_11_O_2_F_13_ [M]^+^: 470.0551, found 470.0528.

#### 3.3.2. 3,3,3-Trifluoro-1-(4-methoxyphenyl) propan-1-ol (**4aa**)

The compound was synthesized as described in oxidative reaction conditions. The product was purified by column chromatography (Hexane/EtOAc = 10:1). Yellow oil (23.3 mg, 35%); ^1^H NMR (400 MHz, CDCl_3_) δ 7.29 (2H, d, *J* = 8.4 Hz), 6.90 (2H, d, *J* = 8.8 Hz), 5.02 (1H, d, *J* = 8.8 Hz), 3.81 (3H, s), 2.70–2.56 (1H, m), 2.49–2.36 (1H, m), 2.16 (1H, s); ^13^C NMR (151 MHz, CDCl_3_) δ 159.7, 134.6, 130.8–112.3 (m), 127.1 (2C), 114.3 (2C), 68.5 (dd, *J* = 3.0, 6.0 Hz), 55.5, 42.8 (dd, *J* = 27.2, 54.3 Hz); ^19^F NMR (376 MHz, CDCl_3_) δ −64.2 (3F, s); IR; 3425, 3007, 2911, 2840, 1613, 1514, 1241, 1123, 1102, 1032, 824; HRMS (EI^+^) calcd for C_10_H_11_O_2_F_3_ [M]^+^: 220.0711, found 220.0718.

#### 3.3.3. 3,3,4,4,4-Pentafluoro-1-(4-methoxyphenyl) tetran-1-ol (**4ab**)

The compound was synthesized as described in oxidative reaction conditions. The product was purified by column chromatography (Hexane/EtOAc = 10:1). Yellow solid (25.9 mg, 32%); ^1^H NMR (400 MHz, CDCl_3_) δ 7.31 (2H, d, *J* = 8.8 Hz), 6.86 (2H, d, *J* = 8.8 Hz), 5.15 (1H, dt, *J* = 8.8, 3.2 Hz), 3.81 (3H, s), 2.66–2.51 (1H, m), 2.41–2.12 (1H, m), 2.11 (1H, s); ^13^C NMR (151 MHz, CDCl_3_) δ 159.7, 134.9, 127.1, 120.0–113.4 (2C, m), 114.3, 67.7, 55.5, 39.7 (t, *J* = 21.0 Hz); ^19^F NMR (376 MHz, CDCl_3_) δ −86.3 (3F, s), −117.1 (1F, dd, *J* = 263.3, 37.6 Hz), −118.3 (1F, dd, *J* = 263.3, 37.6 Hz); IR; 3441, 3007, 2914, 2842, 1614, 1515, 1248, 1186, 1065, 1033; HRMS (EI^+^) calcd for C_11_H_11_O_2_F_5_ [M]^+^: 270.0679, found 270.0678.

#### 3.3.4. 3,3,4,4,5,5,5-Heptafluoro-1-(4-methoxyphenyl) pentan-1-ol (**4ac**)

The compound was synthesized as described in oxidative reaction conditions. The product was purified by column chromatography (Hexane/EtOAc = 10:1). Yellow oil (50.3 mg, 52%); ^1^H NMR (500 MHz, CDCl_3_) δ 7.30 (2H, d, *J* = 8.5 Hz), 6.91 (2H, d, *J* = 9.0 Hz), 5.16 (1H, dd, *J* = 8.5, 3.5 Hz), 3.81 (3H, s), 2.67–2.54 (1H, m), 2.44–2.32 (1H, m), 2.18 (1H, s); ^13^C NMR (151 MHz, CDCl_3_) δ 159.7, 134.9, 134.9–108.6 (3C, m), 127.1 (2C), 114.3 (2C), 67.6, 55.5, 39.5 (t, *J* = 21.0); ^19^F NMR (471 MHz, CDCl_3_) δ −80.9 (3F, s), −114.0 (1F, dd, *J* = 282.3, 47.1 Hz), −115.4 (1F, dd, *J* = 282.3, 47.1 Hz), −128.4 (2F, s); IR; 3455, 3007, 2842, 1613, 1515, 1304, 1155, 832, 713; HRMS (EI^+^) calcd for C_12_H_11_O_2_F_7_ [M]^+^: 320.0647, found 320.0657.

#### 3.3.5. 3,4,4,4-Tetrafluoro-3-trifluoromethyl-1-(4-methoxyphenyl) tetran-1-ol (**4ad**)

The compound was synthesized as described in oxidative reaction conditions. The product was purified by column chromatography (Hexane/EtOAc = 10:1). Yellow oil (57.8 mg, 60%); ^1^H NMR (500 MHz, CDCl_3_) δ 7.30 (2H, d, *J* = 8.5 Hz), 6.91 (2H, d, *J* = 8.5 Hz), 5.12 (1H, d, *J* = 22.5 Hz), 3.81 (3H, s), 2.66–2.57 (1H, m), 2.41–2.33 (1H, m), 2.13 (1H, s); ^13^C NMR (151 MHz, CDCl_3_) δ 159.7, 153.73, 128.9–113.9 (2C, m), 126.9 (2C), 114.3 (2C), 92.3–90.6 (m), 68.3 (d, *J* = 3.0 Hz), 55.5, 38.2 (t, *J* = 18.0 Hz); ^19^F NMR (471 MHz, CDCl_3_) δ −76.9 (3F, s), −77.5 (3F, s), −186.0 (1F, s); IR; 3455, 3007, 2917, 2842, 1613, 1515, 1304, 1217, 1155, 1036, 832, 713; HRMS (EI^+^) calcd for C_12_H_11_O_2_F_7_ [M]^+^: 320.0647, found 320.0652.

#### 3.3.6. 3,3,4,4,5,5,6,6,6-Nonafluoro-1-(4-methoxyphenyl) hexan-1-ol (**4ae**)

The compound was synthesized as described in oxidative reaction conditions. The product was purified by column chromatography (Hexane/EtOAc = 10:1). Yellow oil (46.4 mg, 42%); ^1^H NMR (500 MHz, CDCl_3_) δ 7.31 (2H, d, *J* = 8.5 Hz), 6.91 (2H, dd, *J* = 9.0 Hz), 5.16 (1H, dd, *J* = 8.5, 3.5 Hz), 3.81 (3H, s), 2.68–2.55 (1H, m), 2.45–2.34 (1H, m), 2.17 (1H, s); ^13^C NMR (151 MHz, CDCl_3_) δ 159.7, 134.9, 127.1 (2C), 119.5–108.7 (4C, m), 114.3 (2C), 67.7, 55.5, 39.7 (t, *J* = 21.0 Hz); ^19^F NMR (471 MHz, CDCl_3_) δ −81.3 (3F, s), −113.0 (1F, dd, *J* = 235.3, 47.1 Hz), −114.4 (1F, dd, *J* = 235.3, 47.1 Hz), −124.9 (2F, s), −126.2; IR; 3426, 2842, 2248, 1614, 1515, 1132, 1132, 1034, 881; HRMS (EI^+^) calcd for C_13_H_11_O_2_F_9_ [M]^+^: 370.0615, found 370.0617.

#### 3.3.7. 3,3,4,4,5,5,6,6,7,7,8,8,9,9,10,10,10-Heptadecafluoro-1-(4-methoxyphenyl) decan-1-ol (**4af**)

The compound was synthesized as described in oxidative reaction conditions. The product was purified by column chromatography (Hexane/EtOAc = 10:1). Yellow oil (78.1 mg, 47%); ^1^H NMR (500 MHz, CDCl_3_) δ 7.31 (2H, d, *J* = 8.5 Hz), 6.91 (2H, dd, *J* = 8.5 Hz), 5.17 (1H, dt, *J* = 5.5, 3.0 Hz), 3.81 (3H, s), 2.68–2.56 (1H, m), 2.45–2.34 (1H, m), 2.15 (1H, s); ^13^C NMR (151 MHz, CDCl_3_) δ 159.3, 134.9, 127.1 (2C), 119.7–108.4 (8C, m), 114.3 (2C), 67.7, 55.5, 39.9 (t, *J* = 21.0 Hz); ^19^F NMR (471 MHz, CDCl_3_) δ −81.3 (3F, s), −113.0 (1F, d, *J* = 282.3 Hz), −114.3 (1F, d, *J* = 282.3 Hz), −122.1 (2F, s), −122.4 (4F, s), −123.2 (2F, s), −124.1 (2F, s), −126.6 (2F, s); IR; 3326, 1517, 1199, 1145, 832; HRMS (EI^+^) calcd for C_17_H_11_O_2_F_17_ [M]^+^: 570.0487, found 570.0480.

#### 3.3.8. 1-(4-tert-Buthoxyphenyl)3,3,4,4,5,5,6,6,7,7,8,8,8-tridecafluorooctan-1-ol (**4c**)

The compound was synthesized as described in oxidative reaction conditions. The product was purified by column chromatography (Hexane/EtOAc = 10:1). Yellow oil (88.9 mg, 58%); ^1^H NMR (500 MHz, CDCl_3_) δ 7.27 (2H, d, *J* = 8.5 Hz), 6.98 (2H, d, *J* = 8.5 Hz), 5.18 (1H, d, *J* = 9.0 Hz), 2.68–2.56 (1H, m), 2.46–2.35 (1H, m), 2.28 (1H, s), 1.34 (3H, s); ^13^C NMR (151 MHz, CDCl_3_) δ 155.5, 137.6, 126.4 (2C), 124.6 (2C), 120.2–110.4 (6C, m), 79.0, 67.7, 39.9 (t, *J* = 21.5 Hz), 29.0; ^19^F NMR (471 MHz, CDCl_3_) δ −81.3 (3F, s), −112.9 (1F, d, *J* = 282.3 Hz), −114.4 (1F, d, *J* = 282.3 Hz), −122.3 (2F, s), −123.4 (2F, s), −124.1 (2F, s), −126.7 (2F, s); IR; 3432, 2981, 1608, 1508, 1234, 1191, 707; HRMS (ESI^+^) calcd for C_18_H_17_O_2_F_13_ [M+Na]^+^: 535.0913, found 535.0913.

#### 3.3.9. 1-(4-(tert-butyl)phenyl)-3,3,4,4,5,5,6,6,7,7,8,8,8-tridecafluorooctan-1-ol (**4d**)

The compound was synthesized as described in oxidative reaction conditions. The product was purified by column chromatography (Hexane/EtOAc = 10:1). Yellow oil (52.3 mg, 35%); ^1^H NMR (500 MHz, CDCl_3_) δ 7.41 (2H, d, *J* = 9.0 Hz), 7.32 (2H, d, *J* = 8.0 Hz), 5.20 (1H, dd, *J* = 9.0, 3.0 Hz), 2.69–2.56 (1H, m), 2.47–2.36 (1H, m), 2.18 (1H), 1.32 (9H, s); ^13^C NMR (151 MHz, CDCl_3_) δ 151.7, 139.8, 130.2, 128.4, 126.0, 125.5, 119.7–109.4 (6C, m), 67.9, 39.9 (*J* = 20.4 Hz), 34.7, 31.4 (3C); ^19^F NMR (471 MHz, CDCl_3_) δ −81.3 (3F, s), −112.9 (1F, d, *J* = 282.3 Hz), −114.5 (1F, d, *J* = 282.3 Hz), −123.3 (2F, s), −123.4 (2F, s), −124.1 (2F, s), −126.6 (2F, s); IR; 3400, 2966, 2908, 2872, 1512, 1364, 1235, 1143, 836, 731, 707; HRMS (EI^+^) calcd for C_18_H_17_OF_13_ [M]^+^: 496.1072, found 496.1085.

#### 3.3.10. 3,3,4,4,5,5,6,6,7,7,8,8,8-Tridecafluoro-1-(4-toyl) octan-1-ol (**4e**)

The compound was synthesized as described in oxidative reaction conditions. The product was purified by column chromatography (Hexane/EtOAc = 10:1). Yellow oil (30.6 mg, 22%); ^1^H NMR (400 MHz, CDCl_3_) δ 7.29 (2H, d, *J* = 8.4 Hz), 7.20 (2H, d, *J* = 8.0 Hz), 5.20 (1H, dd, *J* = 8.8, 3.2 Hz), 2.70–2.55 (1H, m), 2.47–2.32 (1H, m), 2.36 (3H, s), 2.11 (1H, s); ^13^C NMR (151 MHz, CDCl_3_) δ 139.9, 138.5, 130.3, 129.7, 125.7, 122.8, 122.8–108.7 (6C, m), 68.0, 39.9 (d, *J* = 20.4 Hz), 21.3; ^19^F NMR (376 MHz, CDCl_3_) δ −81.3 (3F, s), −112.9 (1F, d, *J* = 263.3 Hz), −114.4 (1F, d, *J* = 263.3 Hz) (1F, s), −123.3 (2F, s), −123.4 (2F, s), −124.1 (2F, s), −126.6 (2F, s); IR; 3423, 3030, 2928, 1517, 1364, 1232, 1144, 814, 707; HRMS (EI^+^) calcd for C_15_H_10_OF_13_ [M + H]^+^: 454.0602, found 454.0602.

#### 3.3.11. 3,3,4,4,5,5,6,6,7,7,8,8,8-Tridecafluoro-1-phenyl octan-1-ol (**4f**)

The compound was synthesized as described in oxidative reaction conditions. The product was purified by column chromatography (Hexane/EtOAc = 10:1). Yellow oil (47.5 mg, 36%); ^1^H NMR (500 MHz, CDCl_3_) δ 7.39 (4H, d, *J* = 4.5 Hz), 7.36–7.32 (1H, m), 5.22 (1H, dd, *J* = 9.0, 3.5 Hz), 2.69–2.57 (1H, m), 2.48–2.36 (1H, m), 2.27 (1H, br); ^13^C NMR (151 MHz, CDCl_3_) δ 142.8, 129.0 (2C), 128.6, 125.8 (2C), 120.2–108.7 (6C, m), 68.1, 40.0 (t, *J* = 21.0 Hz); ^19^F NMR (471 MHz, CDCl_3_) δ −81.3 (3F, s), −112.9 (1F, d, *J* = 282.3 Hz), −114.3 (1F, d, *J* = 282.3 Hz), −122.3 (2F, s), −123.4 (2F, s), −124.1 (2F, s), −126.6 (2F, s); IR; 3409, 3069, 3036, 1232, 1189, 1070, 607; HRMS (EI^+^) calcd for C_14_H_9_OF_13_ [M]^+^: 440.0446, found 440.0450.

#### 3.3.12. 3,3,4,4,5,5,6,6,7,7,8,8,8-Tridecafluoro-1-(4-fluorophenyl) octan-1-ol (**4g**)

The compound was synthesized as described in oxidative reaction conditions. The product was purified by column chromatography (Hexane/EtOAc = 10:1). Yellow oil (27.4 mg, 20%); ^1^H NMR (500 MHz, CDCl_3_) δ 7.40–7.36 (2H, m), 7.10–7.06 (2H, m), 5.23 (1H, dd, *J* = 8.5, 3.5 Hz), 2.67–2.55 (1H, m), 2.45–2.33 (1H, m), 2.19 (1H, br); ^13^C NMR (151 MHz, CDCl_3_) δ 162.7 (d, *J* = 247.5 Hz), 138.5 (d, *J* = 3.0 Hz), 127.6 (2C), 118.5–100.3 (6C, m), 118.2 (2C), 67.5, 40.1 (t, *J* = 21.0 Hz); ^19^F NMR (471 MHz, CDCl_3_) δ −81.3 (3F, s), −112.9 (1F, d, *J* = 282.3 Hz), −114.0 (1F, s), −114.2 (1F, d, *J* = 282.3 Hz), −122.3 (2F, s), −123.3 (2F, s), −124.1 (2F, s), −126.6 (2F, s); IR; 3436, 1607, 1512, 1228, 1187, 1142, 838, 707; HRMS (EI^+^) calcd for C_14_H_8_O_2_F_14_ [M]^+^: 458.0352, found 458.0334.

#### 3.3.13. 1-(4-Chlorophenyl)-3,3,4,4,5,5,6,6,7,7,8,8,8-tridecafluorooctan-1-ol (**4h**)

The compound was synthesized as described in oxidative reaction conditions. The product was purified by column chromatography (Hexane/EtOAc = 10:1). Yellow oil (36.3 mg, 25%); ^1^H NMR (400 MHz, CDCl_3_) δ 7.35 (4H, dd, *J* = 13.2, 8.8 Hz), 5.22 (1H, d, *J* = 8.8 Hz), 2.63–2.57 (1H, m), 2.44–2.33 (1H, m), 2.20 (1H, s); ^13^C NMR (151 MHz, CDCl_3_) δ 141.1, 134.3, 129.2 (2C), 127.2 (2C), 119.6–108.4 (6C, m), 67.5, 40.0 (t, *J* = 21.0 Hz); ^19^F NMR (376 MHz, CDCl_3_) δ −81.3 (3F, s), −112.9 (1F, d, *J* = 263.3 Hz), −114.2 (1F, d, *J* = 263.3 Hz), 122.3 (2F, s), −123.4 (2F, s), −124.1 (2F, s), −126.6 (2F, s); IR; 3413, 1494, 1364, 1232, 1187, 1144, 831, 699; HRMS (EI^+^) calcd for C_14_H_8_OClF_13_ [M]^+^: 474.0056, found 474.0062.

#### 3.3.14. 1-([1,1′-biphenyl]-4-yl)-3,3,4,4,5,5,6,6,7,7,8,8,8-tridecafluorooctan-1-ol (**4i**)

The compound was synthesized as described in oxidative reaction conditions. The product was purified by column chromatography (Hexane/EtOAc = 10:1). White solid (35.0 mg, 27%); ^1^H NMR (400 MHz, CDCl_3_) δ7.62–7.57 (4H, m), 7.50–7.43 (4H, m), 7.38–7.34 (1H, m), 5.29–5.25 (1H, m), 2.74–2.56 (1H, m), 2.53–2.39 (1H, m), 2.29 (1H, d, *J* = 1.8 Hz); ^13^C NMR (151 MHz, CDCl_3_) δ 141.7, 141.6, 140.6, 132.6, 130.2, 129.0, 128.4, 127.8, 127.7, 127.3, 126.2, 120.2–108.4 (6C, m), 67.9, 40.0 (t, *J* = 20.4 Hz); ^19^F NMR (471 MHz, CDCl_3_) δ −81.2 (3F, s), −112.8 (1F, d, *J* = 134.9 Hz), −114.2 (1F, d, *J* = 134.9 Hz), −122.2 (2F, s), −123.3 (2F, s), −124.0 (2F, s), −126.6 (2F, s); IR; 3299, 1234, 1189, 1144, 1007, 839, 766, 692, 651; HRMS (ESI^+^) calcd for C_20_H_13_OF_13_ [M+Na]^+^: 539.0651, found 539.0644.

#### 3.3.15. 3,3,4,4,5,5,6,6,7,7,8,8,8-Tridecafluoro-1-(4-methoxyphenyl) octan-1-ol (**4j**)

The compound was synthesized as described in oxidative reaction conditions. The product was purified by column chromatography (Hexane/EtOAc = 10:1). Yellow oil (46.8 mg, 33%); ^1^H NMR (400 MHz, CDCl_3_) δ 7.40 (1H, t, *J* = 7.3 Hz), 6.96 (1H, d, *J* = 3.2 Hz), 6.95 (1H, s), 5.19 (1H, dd, *J* = 8.8, 3.2), 3.82 (3H, s), 2.72–2.53 (2H, m), 2.48–2.31 (1H, m); ^13^C NMR (151 MHz, CDCl_3_) δ 160.1, 144.4, 130.1, 120.3–109.2 (6C, m), 117.9, 113.9, 111.3, 68.0, 55.4, 40.0 (t, *J* = 21.0 Hz); ^19^F NMR (376 MHz, CDCl_3_) δ −81.2 (3F, s), −112.8 (1F, d, *J* = 263.3 Hz), −114.3 (1F, d, *J* = 263.3 Hz), −122.2 (2F, s), −123.2 (2F, s), −124.0 (2F, s), −126.5 (2F, s); IR; 3495, 2963, 2842, 1603, 1364, 1221, 1144, 1047, 786, 696; HRMS (EI^+^) calcd for C_15_H_11_O_2_F_13_ [M]^+^: 470.0551, found 470.0559.

#### 3.3.16. 3,3,4,4,5,5,6,6,7,7,8,8,8-Tridecafluoro-1-(4-toyl) octan-1-ol (**4k**)

The compound was synthesized as described in oxidative reaction conditions. The product was purified by column chromatography (Hexane/EtOAc = 10:1). Yellow oil (56.6 mg, 42%); ^1^H NMR (400 MHz, CDCl_3_) δ 7.54 (1H, dd, *J* = 7.6, 1.2 Hz), 7.23 (1H, m), 5.48 (1H, dd, *J* = 8.8, 1.6), 2.68–2.49 (2H, m), 2.42–2.52 (1H, m), 2.34 (1H, s), 2.15 (1H, br); ^13^C NMR (151 MHz, CDCl_3_) δ 140.9, 134.0, 130.9, 128.2, 126.9, 125.2, 120.2–108.4 (6C, m), 64.3, 39.1, 18.9; ^19^F NMR (471 MHz, CDCl_3_) δ −81.3 (3F, s), −113.3 (1F, d, *J* = 263.3 Hz), −117.7 (1F, d, *J* = 263.3 Hz), −122.2 (2F, s), −123.2 (2F, s), −124.1 (2F, s), −126.6 (2F, s); IR; 3416, 3029, 2957, 1364, 1232, 1187, 1144, 812, 707; HRMS (EI^+^) calcd for C_15_H_11_OF_13_ [M]^+^: 454.0602, found 454.0601.

#### 3.3.17. 1-(3,4-Dimethoxyphenyl)-3,3,4,4,5,5,6,6,7,7,8,8,8-tridecafluorooctan-1-ol (**4l**)

The compound was synthesized as described in oxidative reaction conditions. The product was purified by column chromatography (Hexane/EtOAc = 10:1). Yellow oil (85.0 mg, 68%); ^1^H NMR (400 MHz, CDCl_3_) δ 6.93–6.85 (3H, m), 5.18 (1H, dd, *J* = 6.18, 2.52 Hz), 3.91 (3H, s), 3.89 (3H, s), 2.70–2.55 (1H, m), 2.47–2.33 (1H, m), 2.18 (1H, s); ^13^C NMR (151 MHz, CDCl_3_) δ149.4, 149.1, 135.4, 119.9–109.1 (6C, m), 118.0, 111.2, 108.6, 68.0, 56.1, 56.0, 40.0 (t, *J* = 21.1 Hz), 29.9; ^19^F NMR (376 MHz, CDCl_3_) δ −81.3 (3F, s), −112.9 (1F, d, *J* = 289.0 Hz), −114.4 (1F, d, *J* = 289.0 Hz), −122.3 (2F, s), −123.3 (2F, s), −124.1 (2F, s), −126.6 (2F, s); IR; 3498, 2841, 1518, 1231, 1188, 1141, 1116, 1026, 809, 765, 735, 707; HRMS (ESI^+^) calcd for C_16_H_13_O_3_F_13_ [M + Na]^+^: 523.0549, found 523.0557.

#### 3.3.18. 2-(1,1,2,2,3,3,4,4,5,5,6,6,6-Tridecafluorohexyl)-1,2,3,4-tetrahydronaphthalen-1-ol (**4m**)

The compound was synthesized as described in oxidative reaction conditions (62:38 diastereomer mixture, dr was measured by crude ^1^H NMR). The product was purified by column chromatography (Hexane/EtOAc = 10:1). Yellow oil (43.5 mg, 31%); ^1^H NMR (400 MHz, CDCl_3_) δ 7.50–7.11 (major and minor, 4H, m), 5.14 (major, 1H, t, *J* = 6.5 Hz), 5.10 (minor, 1H, s), 3.07–2.52 (major and minor, 3H, m), 2.33–2.09 (major and minor, 2H, m), 1.92–1.83 (major and minor, 1H, m); ^13^C NMR (151 MHz, CDCl_3_) δ 136.7 (minor), 136.4 (major), 132.6 (minor), 130.2 (major), 129.3 (minor), 129.0 (minor), 128.8 (major), 128.5 (major), 128.4 (minor), 128.2 (major), 127.0 (major), 126.8 (minor), 121.0–106.9 (major and minor, 6C, m), 66.9 (major), 66.1 (minor), 45.6 (major, t, *J* = 19.6 Hz), 42.4 (minor, t, *J* = 19.6 Hz), 28.5 (minor), 27.5 (major), 20.6 (major), 16.2 (minor); ^19^F NMR (471 MHz, CDCl_3_) δ −81.2 (major and minor, 3F, s), −113.5 (major, 1F, d, *J* = 288.8 Hz), −114.4 (minor, 1F, d, *J* = 288.8 Hz), −116.0 (minor, 1F, d, *J* = 288.8 Hz), −117.0 (major, 1F, d, *J* = 288.8 Hz), −120.5–−124.1 (major and minor, 6F, m), −125.7–−127.4 (major and minor, 2F, m); IR; 3447, 3063, 3013, 2986, 2936, 2884, 1491, 1451, 1227, 1190, 1179, 1142, 1121, 1045, 743; HRMS (EI^+^) calcd for C_16_H_11_OF_13_ [M]^+^: 466.0602, found 466.0565.

#### 3.3.19. 3,3,4,4,5,5,6,6,7,7,8,8,8-Tridecafluoro-1-(4-(trifluoromthyl)phenyl) octan-1-ol (**4n**)

The compound was synthesized as described in oxidative reaction conditions. The product was purified by column chromatography (Hexane/EtOAc = 10:1). Yellow oil (25.9 mg, 17%); ^1^H NMR (400 MHz, CDCl_3_) δ 7.66 (2H, d, *J* = 8.0 Hz), 7.54 (2H, d, *J* = 8.0 Hz), 5.31 (1H, d, *J* = 8.6 Hz), 2.68–2.56 (1H, m), 2.48–2.36 (1H, m), 2.28 (1H, s); ^13^C NMR (151 MHz, CDCl_3_) δ: 146.3, 132.6, 130.8 (dd, *J* = 64.9, 33.2 Hz), 130.2, 128.3, 126.2, 126.0 (q, *J* = 3.0 Hz), 121.3–106.5 (6C, m), 67.5, 40.1 (t, *J* = 21.1 Hz); ^19^F NMR (376 MHz, CDCl_3_) δ −63.1 (3F, s), −81.2 (3F, s), −112.8 (1F, d, *J* = 265.8 Hz), −114.0 (1F, d, *J* = 265.8 Hz), −122.2 (2F, s), −123.3 (2F, s), −124.0 (2F, s), −126.6 (2F, s); IR; 3443, 1326, 1237, 1129, 1069, 1017, 844, 707; HRMS (EI^+^) calcd for C_15_H_8_OF_16_ [M]^+^: 508.0320, found 508.0320.

#### 3.3.20. 3,3,4,4,5,5,6,6,7,7,8,8,8-Tridecafluoro-1-(2,3,4,5,6-pentafluorophenyl) octan-1-ol (**4o**)

The compound was synthesized as described in oxidative reaction conditions. The product was purified by column chromatography (Hexane/EtOAc = 10:1). Yellow oil (27.2 mg, 17%); ^1^H NMR(500 MHz, CDCl_3_) δ 5.60 (1H, dd, *J* = 12.5, 6.0 Hz), 3.00–2.89 (1H, m), 2.72–2.61 (1H, m), 2.47 (1H, d, *J* = 6.5 Hz); ^13^C NMR (151 MHz, CDCl_3_) δ; 145.7, 144.1, 138.6 (2C), 137.0 (2C), 119.2–106.7 (6C, m), 59.4, 37.3 (t, *J* = 19.5 Hz); ^19^F NMR (471 MHz, CDCl_3_) δ −81.3 (3F, s), −113.8 (1F, d, *J* = 282.3 Hz), −114.7 (1F, d, *J* = 282.3 Hz) −122.3 (2F, s), −123.4 (2F, s), −124.0 (2F, s), −126.6 (2F, s), −143.7 (2F, d, *J* = 47.1 Hz), −153.4 (1F, d, *J* = 47.1 Hz), 161.2 (2F, s); IR; 3486, 1656, 1523, 1504, 1235, 1192, 1142, 996, 947, 700; HRMS (EI^+^) calcd for C_14_H_4_OF_18_ [M]^+^: 529.9975, found 529.9976.

#### 3.3.21. 3,3,4,4,5,5,6,6,7,7,8,8,8-Tridecafluoro-1,1-diphenyloctan-1-ol (**4p**)

The compound was synthesized as described in oxidative reaction conditions. The product was purified by column chromatography (Hexane/EtOAc = 10:1). Yellow oil (60.3 mg, 43%); ^1^H NMR (400 MHz, CDCl_3_) δ 7.45–7.42 (4H, m), 7.36–7.31 (4H, m), 7.28–7.24 (2H, m), 3.17 (2H, t, *J* = 18.3 Hz), 2.74 (1H, t, *J* = 2.1 Hz); ^13^C NMR (151 MHz, CDCl_3_) δ: 145.5 (2C), 137.7, 132.6, 130.2, 128.6 (2C), 128.4, 127.7 (2C), 125.5 (2C), 120.1–108.4 (6C, m), 76.6, 41.0 (t, *J* = 19.6 Hz); ^19^F NMR (376 MHz, CDCl_3_) δ −81.3 (3F, s), −109.5 (2F, s), −122.1 (2F, s), −123.3 (2F, s), −124.1 (2F, s), −126.6 (2F, s); IR; 3470, 3088, 3061, 2971, 3028, 1655, 1495, 1233, 1188, 1142, 1121, 812, 696; HRMS (ESI^−^) calcd for C_20_H_13_OF_13_ [M − H]^−^: 515.0681, found 515.0641.

#### 3.3.22. 4-(3,3,4,4,5,5,6,6,7,7,8,8,8-Tridecafluorooctyl)benzonitrile (**5b**)

The compound was synthesized as described in reductive reaction conditions. The product was purified by column chromatography (Hexane). Yellow oil (96.5 mg, 72%); ^1^H NMR (400 MHz, CDCl_3_) δ 7.64–7.61 (2H, m), 7.34 (2H, d, *J* = 8.2 Hz), 3.01–2.98 (2H, m), 2.46–2.33 (2H, d); ^13^C NMR (151 MHz, CDCl_3_) δ: 144.7, 132.8 (2C), 129.3 (2C), 119.8–108.4 (6C, m), 118.8, 111.0, 32.4 (t, *J* = 22.7 Hz), 26.8 (t, *J* = 4.5 Hz); ^19^F NMR (376 MHz, CDCl_3_) δ −81.2 (3F, s) −115.0 (2F, s), −122.3 (2F, s), −123.3 (2F, s), −123.9 (2F, s), −126.6 (2F, s); IR; 3062, 2227, 1185, 1165, 1119, 1086, 1072, 979, 908, 862, 812, 736, 704; HRMS (ESI^+^) calcd for C_15_H_8_F_13_N [M + Na]^+^: 472.0347, found 472.0347.

#### 3.3.23. 4-(3,3,4,4,5,5,5-Heptafluoropentyl)benzonitrile (**5bc**)

The compound was synthesized as described in reductive reaction conditions. The product was purified by column chromatography (Hexane). Yellow oil (57.4 mg, 64%); ^1^H NMR (400 MHz, CDCl_3_) δ 7.63 (2H, dd, *J* = 6.6, 1.6 Hz), 7.33 (2H, d, *J* = 8.2 Hz), 3.02–2.97 (2H, m), 2.45–2.32 (2H, d); ^13^C NMR (151 MHz, CDCl_3_) δ: 144.6, 132.7 (2C), 129.3 (2C), 120.7–107.0 (3C, m), 118.8, 111.0, 32.1 (t, *J* = 22.7 Hz), 26.7 (t, *J* = 3.0 Hz); ^19^F NMR (376 MHz, CDCl_3_) δ −81.0 (3F, s) −116.0 (2F, s), −128.2 (2F, s); IR; 2961, 2231, 1352, 1220, 1169, 1112, 1085, 947, 910, 824, 743, 680; HRMS (ESI^+^) calcd for C_12_H_8_F_7_N [M + Na]^+^: 322.0442, found 322.0443.

#### 3.3.24. 4-(3,3,4,4,5,5,6,6,6-Nonafluorohexyl)benzonitrile (**5be**)

The compound was synthesized as described in reductive reaction conditions. The product was purified by column chromatography (Hexane). Yellow oil (76.0 mg, 73%); ^1^H NMR (400 MHz, CDCl_3_) δ 7.65–7.62 (2H, m), 7.34 (2H, d, *J* = 8.2 Hz), 3.02–2.98 (2H, m), 2.46–2.33 (2H, d); ^13^C NMR (151 MHz, CDCl_3_) δ: 144.6, 132.8 (2C), 129.3 (2C), 120.3–108.7 (4C, m), 118.8, 111.0, 32.3 (t, *J* = 22.7 Hz), 26.8 (t, *J* = 3.0 Hz); ^19^F NMR (376 MHz, CDCl_3_) δ −81.5 (3F, s) −115.2 (2F, s), −124.9 (2F, s), −126.5 (2F, s); IR; 2960, 2231, 1357, 1217, 1131, 1088, 1009, 976, 881, 824, 722, 692; HRMS (ESI^+^) calcd for C_13_H_8_F_9_N [M + Na]^+^: 372.0411, found 372.0411.

#### 3.3.25. 1-(3,3,4,4,5,5,6,6,7,7,8,8,8-Tridecafluorooctyl)-4-(trifluoromethyl)benzene (**5n**)

The compound was synthesized as described in reductive reaction conditions. The product was purified by column chromatography (Hexane). Yellow oil (63.8 mg, 65%); ^1^H NMR (500 MHz, CDCl_3_) δ 7.59 (2H, d, *J* = 8.0 Hz), 7.34 (2H, d, *J* = 8.0 Hz), 2.99 (2H, tt, *J* = 8.5, 3.0 Hz), 2.39 (2H, qt, *J* = 19.0, 18.5 Hz); ^13^C NMR (151 MHz, CDCl_3_) δ143.3, 129.3 (q, *J* = 33.2 Hz), 128.8, 125.9 (q, *J* = 3.0 Hz), 119.9–108.4 (6C, m), 32.7 (t, *J* = 21.1 Hz), 26.5 (t, *J* = 4.5 Hz); ^19^F NMR (471 MHz, CDCl_3_) δ −62.3 (3F, s), −81.3 (3F, s) −115.0 (2F, d, *J* = 272.5), −122.4 (2F, s), −123.4 (2F, s), −124.0 (2F, s), −126.6 (2F, s); IR; 1612, 1422, 1325, 1235, 1166, 1069, 1020, 826, 707; HRMS (APCI^+^) calcd for C_15_H_8_F_16_ [M]^+^: 492.0370 found, 492.0370.

#### 3.3.26. 1,2,3,4,5-Pentafluoro-6-(3,3,4,4,5,5,6,6,7,7,8,8,8-tridecafluorooctyl)benzene (**5o**)

The compound was synthesized as described in reductive reaction conditions. The product was purified by column chromatography (Hexane). Colorless oil (54.5 mg, 53%); ^1^H NMR (500 MHz, CDCl_3_) δ 2.99 (2H, t, *J* = 7.5 Hz), 2.38 (2H, qt, *J* = 18.0, 18.0 Hz); ^13^C NMR (150 MHz, CDCl_3_) δ 146.1–136.8 (6C, m), 120.2–108.4 (6C, m), 30.2 (t, *J* = 22.6 Hz), 14.0; ^19^F NMR (471 MHz, CDCl_3_) δ −81.2 (3F, s), −115.8 (2F, d, *J* = 272.5), −122.4 (2F, s), −123.4 (2F, s), −124.0 (2F, s), −126.6 (2F, s), −144.3 (2F,s), −156.0 (2F,s), −162.2 (2F,s); IR; 1659, 1522, 1506, 1232, 1191, 1144, 1013, 968, 697; HRMS (FD^+^) calcd for C_14_H_4_F_18_ [M − H]^+^: 512.9947 found 512.9938.

#### 3.3.27. (3,3,4,4,5,5,6,6,7,7,8,8,8-Tridecafluorooctane-1,1-diyl)dibenzene (**5p**)

The compound was synthesized as described in reductive reaction conditions. The product was purified by column chromatography (Hexane). Yellow oil (64.0 mg, 64%); ^1^H NMR (500 MHz, CDCl_3_) δ 7.32–7.26 (8H, m), 7.24–7.19 (2H, m), 4.46 (1H, t, *J* = 7.2), 2.92 (2H, td, *J* = 28.4, 11.2); ^13^C NMR (151 MHz, CDCl_3_) δ 143.2 (2C), 128.9 (4C), 127.6 (4C), 127.0 (2C), 120.2–108.4 (6C, m), 43.8, 36.3 (t, *J* = 19.6 Hz); ^19^F NMR (471 MHz, CDCl_3_) δ −81.3 (3F, s), −113.3 (2F, s), −122.2 (2F, s), −123.3 (2F, s), 124.0 (2F, s), −126.6 (2F, s); IR; 3090, 3066, 3032, 1601, 1495, 1364, 1235, 1186, 1144, 696; HRMS (ESI^+^) calcd for C_20_H_13_F_18_ [M + Na]^+^: 523.0702 found 523.0702.

#### 3.3.28. 1-Nitro-4-(3,3,4,4,5,5,6,6,7,7,8,8,8-tridecafluorooctyl)benzene (**5q**)

The compound was synthesized as described in reductive reaction conditions. The product was purified by column chromatography (Hexane). Yellow oil (19.7 mg, 21%); ^1^H NMR (500 MHz, CDCl_3_) δ 8.20 (2H, d, *J* = 8.4, 2.8 Hz), 7.40 (2H, d, *J* = 8.8 Hz), 3.05 (2H, tt, *J* = 8.4, 2.4 Hz), 2.42 (2H, qt, *J* = 17.6, 18.8 Hz); ^13^C NMR (151 MHz, CDCl_3_) δ 147.1, 146.7, 129.4 (2C), 124.2 (2C), 119.6–105.8 (6C, m), 32.4 (t, *J* = 21.0 Hz), 26.6; ^19^F NMR (471 MHz, CDCl_3_) δ −81.3 (3F, s) −115.0 (2F, s), −122.4 (2F, s), −123.4 (2F, s), −123.9 (2F, s), −126.7 (2F, s); IR; 2961, 1611, 1534, 1349, 1198, 1138, 1073, 977, 855, 782, 752, 693; HRMS (ESI^+^) calcd for C_14_H_8_NO_2_F_13_ [M + Na]^+^: 492.0240 found, 492.0240.

#### 3.3.29. 1-Methoxy-4-(3,3,4,4,5,5,6,6,7,7,8,8,8-tridecafluoro-1-methoxyoctyl)benzene (**6a**)

The compound was synthesized as described in oxidative reaction conditions using methanol instead of water. The product was purified by column chromatography (Hexane/EtOAc = 10:1). Colorless oil (71.0 mg, 48%); ^1^H NMR (400 MHz, CDCl_3_) δ 7.26 (2H, d, *J* = 8.8 Hz), 6.92 (2H, d, *J* = 8.8 Hz), 4.52 (1H, dd, *J* = 8.4, 4.0 Hz), 3.82 (3H, s), 3.20 (3H, s), 2.71–2.55 (1H, m), 2.39–2.25 (1H, m); ^13^C NMR (151 MHz, CDCl_3_) δ 159.7, 132.4, 127.9 (2C), 119.3–111.0 (6C, m), 114.4 (2C), 76.4, 56.5, 55.3, 39.3 (t, *J* = 21.0); ^19^F NMR (376 MHz, CDCl_3_) δ −81.3 (3F, s), −112.7 (1F, d, *J* = 225.7 Hz), −114.0 (1F, d, *J* = 225.7 Hz), −122.3 (2F, s), −123.4 (2F, s), −124.2 (2F, s), −126.6 (2F, s); IR; 3002, 2940, 2841, 1613, 1514, 1144, 1036, 707; HRMS (EI^+^) calcd for C_16_H_13_O_2_F_13_ [M]^+^: 484.0708, found 484.0732.

#### 3.3.30. 1-(1-Ethoxy-3,3,4,4,5,5,6,6,7,7,8,8,8-tridecafluorooctyl)-4-methoxybenzene (**6b**)

The compound was synthesized as described in oxidative reaction conditions using ethanol instead of water. The product was purified by column chromatography (Hexane/EtOAc = 10:1). Colorless oil (77.3 mg, 52%); ^1^H NMR (400 MHz, CDCl_3_) δ 7.26 (2H, d, *J* = 8.4 Hz), 6.91 (2H, d, *J* = 8.8 Hz), 4.63 (1H, dd, *J* = 8.4, 4.0 Hz), 3.82 (3H, s), 3.20 (3H, s), 3.41–3.29 (2H, m), 2.71–2.56 (1H, m), 2.38–2.24 (1H, m), 1.17 (3H, t, *J* = 6.8 Hz); ^13^C NMR (151 MHz, CDCl_3_) δ 159.6, 133.2, 127.7, 119.3–109.2 (6C, m), 114.2, 74.5, 64.2 55.4, 39.4 (t, *J* = 21.0 Hz), 15.2; ^19^F NMR (471 MHz, CDCl_3_) δ −81.3 (3F, s), −112.7 (2F, d, *J* = 300.9 Hz), −113.9 (2F, d, *J* = 300.9 Hz), −122.3 (2F, s), −123.4 (2F, s), −124.1 (2F, s), −126.6 (2F, s); IR; 2981, 1613, 1514, 1235, 1102, 1037, 832, 707; HRMS (APCI^+^) calcd for C_17_H_15_O_2_F_13_ [M]^+^: 498.0864; found 498.0859.

#### 3.3.31. 1-(1-Butoxy-3,3,4,4,5,5,6,6,7,7,8,8,8-tridecafluorooctyl)-4-methoxybenzene (**6c**)

The compound was synthesized as described in oxidative reaction conditions using *n*-butanol instead of water. The product was purified by column chromatography (Hexane/EtOAc = 10:1). Colorless oil (75.8 mg, 47%); ^1^H NMR (500 MHz, CDCl_3_) δ 7.26 (2H, d, *J* = 8.5 Hz), 6.91 (2H, d, *J* = 8.5 Hz), 4.61 (1H, dd, *J* = 8.5, 3.5 Hz), 3.82 (3H, s), 3.32 (3H, s), 3.41–3.23 (2H, m), 2.66–2.57 (1H, m), 2.33–2.24 (1H, m), 1.54–1.48 (1H, m), 1.38–1.31 (1H, m), 0.87 (3H, t, *J* = 7.0 Hz); ^13^C NMR (151 MHz, CDCl_3_) δ 159.6, 133.2, 127.7 (2C), 119.3–110.4 (6C, m), 114.2 (2C), 74.8, 68.7, 55.4, 39.4 (t, *J* = 4.5 Hz), 31.9, 19.4, 13.0; ^19^F NMR (471 MHz, CDCl_3_) δ −81.3 (3F, s), −112.6 (2F, d, *J* = 272.5 Hz), −114.0 (2F, d, *J* = 272.5 Hz), −122.3 (2F, s), −123.4 (2F, s), −124.1 (2F, s), −126.6 (2F, s); IR; 2963, 2938, 2877, 1613, 1514, 1144, 1102, 1037, 707; HRMS (ESI^+^) calcd for C_19_H_19_O_2_F_13_ [M + Na]^+^: 549.1070 found, 549.1078.

#### 3.3.32. (1,1,1,4,4,5,5,6,6,7,7,8,8,9,9,9-Hexadecafluorononan-2-yl)benzene (**8**)

The compound was synthesized as described in reductive reaction conditions. The product was purified by column chromatography (Hexane). Colorless oil (20.0 mg, 20%); ^1^H NMR (500 MHz, CDCl_3_) 7.55–7.28 (5H, m), 3.78–3.71 (1H, m), 2.84–2.68 (2H, m); ^13^C NMR (151 MHz, CDCl_3_) δ 155.6 (t, *J* = 294.5 Hz), 132.5 (t, *J* = 4.5 Hz), 129.1 (t, *J* = 9.1 Hz), 128.8, 128.7, 128.3, 128.2, 120.6–108.4 (6C, m), 83.7 (t, *J* = 19.6 Hz), 30.7 (t, *J* = 22.7 Hz); ^19^F NMR (471 MHz, CDCl_3_) −71.0 (3F, s), −81.3 (3F, s), −112.4 (2F, d, *J* = 272.7 Hz), −114.7 (2F, d, *J* = 272.7 Hz), −122.4 (2F, s), −123.4 (2F, s), −123.9 (2F, s), −126.7 (2F, s); IR; 1232, 1187, 1141, 1119, 1033, 845, 812, 778, 755, 744; HRMS (FD^+^) calcd for C_15_H_8_F_16_ [M − H]^+^: 491.0293, found 491.0289.

#### 3.3.33. (1,1,4,4,5,5,6,6,7,7,8,8,9,9,9-Pentadecafluoronon-1-en-2-yl)benzene (**9**)

The compound was synthesized as described in reductive reaction conditions. The product was purified by column chromatography (Hexane). Colorless oil (49.0 mg, 51%); ^1^H NMR (500 MHz, CDCl_3_) δ 7.46–7.26 (5H, m), 3.21 (2H, t, *J* = 17.5 Hz); ^13^C NMR (151 MHz, CDCl_3_) δ 129.1 (2C), 128.3 (2C), 128.8, 128.3, 128.2, 120.6–108.2 (7C, m), 43.6 (q, *J* = 27.2 Hz), 31.0 (t, *J* = 28.7 Hz); ^19^F NMR (471 MHz, CDCl_3_) δ −81.3 (3F), −86.0 (1F, d, *J* = 57.9 Hz), −86.4 (1F, d, *J* = 27.3 Hz), −122.7 (2F, s), −122.3 (2F, s), −123.4 (2F, s),−123.7 (2F, s), −126.6 (2F, s); IR; 1736, 1232, 1197, 1139, 868, 847, 812, 799, 778, 755, 707, 696, 663; HRMS (FD^+^) calcd for C_15_H_7_F_15_ [M]^+^: 472.0308, found 472.0327.

## 4. Conclusions

In conclusion, we have achieved the hydroxy- and hydro-perfluoroalkylation of styrenes by controlling the quenching cycle of eosin Y through additive selection. In the oxidative quenching cycle with sodium thiosulfate addition, the reaction of perfluoroalkyl iodide with styrene had electron-donating substituents, producing hydroxy-perfluoroalkylated products. Conversely, in the reductive quenching cycle where amines were added, the reaction of perfluoroalkyl bromide with styrene had electron-withdrawing substituents and yielded hydro-perfluoroalkylated products. These reactions utilize the properties of the organic dye eosin Y and use water as the hydroxyl group or hydrogen source. The reagents used in these reactions are inexpensive and readily available and do not use heavy metals or other harmful reagents, making these reactions environmentally adaptive.

## Figures and Tables

**Table 1:** Optimization of reaction conditions ^a^.

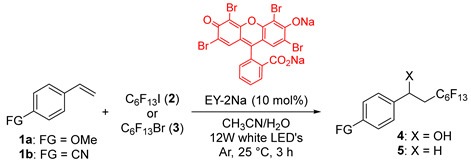					
Entry	1	2 or 3	Additive	Yield of 4 (%) ^b^	Yield of 5 (%) ^b^
1	**1a**	**2** (1.2 equiv.)	Na_2_S_2_O_3_ (5.0 equiv.)	40	n.d. ^c^
2	**1a**	**2** (1.2 equiv.)	Na_2_S_2_O_3_ (5.0 equiv.), K_2_CO_3_ (2.0 equiv.)	53	n.d. ^c^
3	**1a**	**2** (1.2 equiv.)	Na_2_S_2_O_3_ (0.25 equiv.), K_2_CO_3_ (2.0 equiv.)	70 (50)	n.d. ^c^
4	**1a**	**2** (1.2 equiv.)	K_2_CO_3_ (2.0 equiv.)	20	n.d. ^c^
5	**1a**	**2** (3.0 equiv.)	Na_2_S_2_O_3_ (0.25 equiv.), K_2_CO_3_ (2.0 equiv.)	66	n.d. ^c^
6	**1b**	**3** (3.0 equiv.)	DIPEA (2.0 equiv.)	n.d.	70 (72)

^a^ Reaction conditions: The mixture of **1** (0.25 mmol), Eosin Y (10 mol%), styrene (**2** or **3**), and additives in CH_3_CN/H_2_O (7.5 mL/1 mL) under an argon atmosphere was irradiated for 3 h with white LED light at 25 °C. ^b^ Yields were determined by ^19^F NMR using PhCF_3_ as the internal standard. Yields in parentheses are isolated yields. ^c^ n.d.; Not detected.

## Data Availability

The data presented in this study are available in Supplementary Material.

## Supplementary Materials

The following supporting information can be downloaded at: https://www.mdpi.com/article/10.3390/molecules28227577/s1, Scheme S1: Reactions using different perfluoroalkyl halide; Scheme S2: Reactions in the absence of H_2_O. ^1^H, ^13^C and ^19^F NMR spectra of products **4a**, **4c**–**4p**, **4aa**–**4af**, **5b**, **5n**–**5q**, **5bc**–**5be**, **6a**–**c**, **8**, and **9**; Table S1: Oxidative and Reductive reactions with various styrenes; Table S2: Investigation of the number of equivalents of Na_2_S_2_O_3_; Table S3: Investigation of reaction time for oxidative reactions in the absence of K_2_CO_3_; Table S4: Investigation of reaction condition for reductive reaction conditions.
