# Supplementary material for: Hydroxy- and Hydro-Perfluoroalkylation of Styrenes by Controlling the Quenching Cycle of Eosin Y
Source: Molecules. 2023 Nov 14;28(22):7577. doi: 10.3390/molecules28227577 (PMC10674426; doi:10.3390/molecules28227577)

Supporting Information

Hydroxy- and Hydro-Perfluoroalkylation of Styrenes by Controlling the Quenching Cycle of Eosin Y

Haruko Shibata, Moeko Nakayama, Koto Tagami, Tadashi Kanbara, Tomoko Yajima
Department of Chemistry, Ochanomizu University, 2-1-1 Otsuka, Bukyo-ku,
Tokyo 104-8610, Japan

Table of contents

1. Schemes and Tables S2

2. ^{1}H, ^{13}C and ^{19}F NMR spectra S5

1. Schemes and Tables

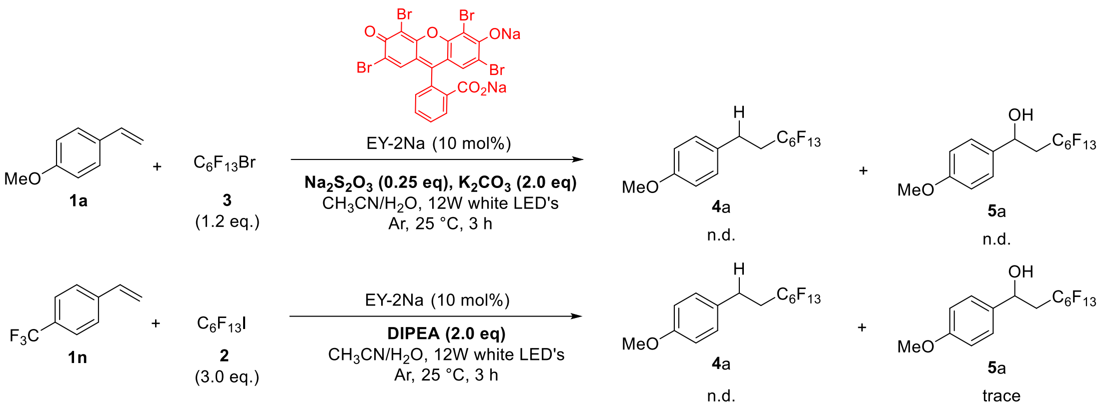

Scheme S1. Reactions using different perfluoroalkyl halide.

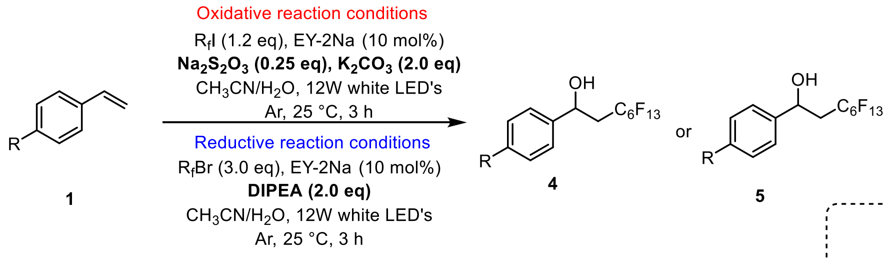

-R	σ_{para}[a]	Oxidative reaction conditions **4**[b]	Reductive reaction conditions **5**[b]
**OMe** (**1a**)	-0.268	70%	n.d.
**Me** (**1e**)	-0.170	27%	n.d.
**H** (**1f**)	0.000	37%	10%
**F** (**1g**)	0.062	22%	5%
**Cl** (**1h**)	0.227	19%	14%
**CF_3** (**1n**)	0.551	17%	65%

a) Hammett Equation., H. H. Jaffe, *Chem. Rev.* **1953,** *53,* 2, 191-261.
b) Determined by crude ^{19}F NMR using BTB as an internal standard.

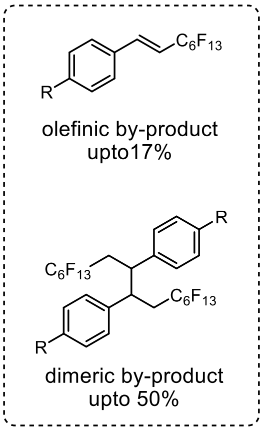

Table S1. Oxidative and Reductive reactions with various styrenes.

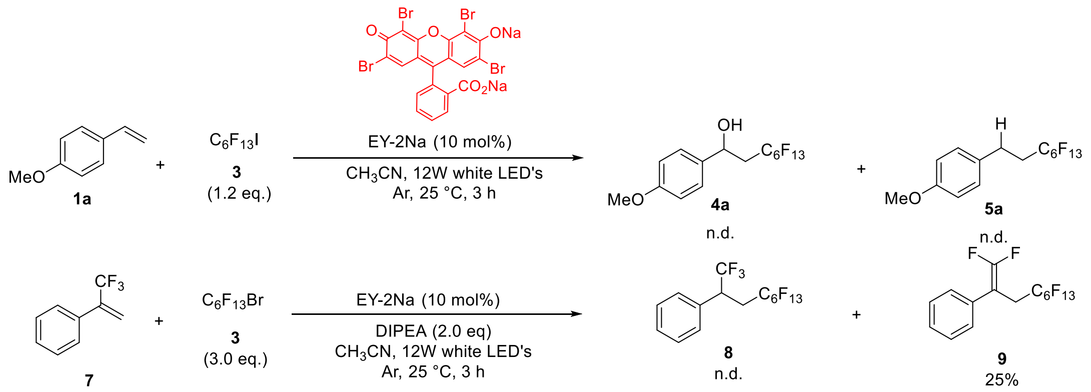

Scheme S2. Reactions in the absence of H_2O.

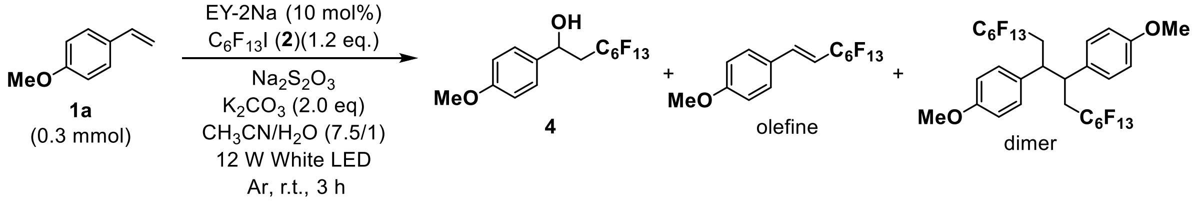

entry	$Na_2S_2O_3$	total yield[a]	**4**	olefine	dimer
1	5.0 eq.	90%	53%	15%	22%
2	1.0 eq.	93%	53%	10%	30%
3	0.5 eq.	88%	65%	15%	8%
4	0.25 eq.	97%	70%	17%	10%
5	0.1 eq.	46%	40%	6%	trace
6	none	22%	20%	2%	n.d.

a) Determined by crude ^{19}F NMR (terminal CF_3) using BTB as an internal standard.

Table S2. Investigation of the number of equivalents of $Na_2S_2O_3$.

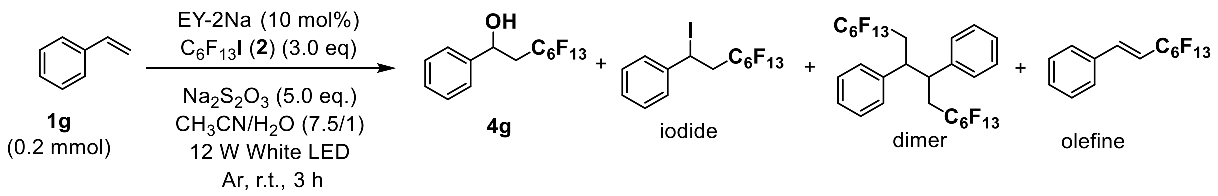

entry	time	total yield[a]	**4g**	iodide	dimer	olefine
1	1 h	70%	15%	21%	4%	30%
2	2 h	68%	13%	9%	4%	42%
3	3 h	75%	26%	n.d.	10%	39%

a) Determined by crude ^{19}F NMR (terminal CF_3) using BTF as an internal standard.

Table S3. Investigation of reaction time for oxidative reactions in the absence of K_2CO_3.

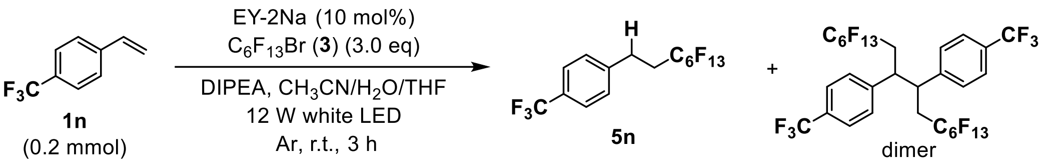

entry	DIPEA	$CH_3CN/H_2O/THF$	total yield[a]	**5n**	dimer
1	2.0 eq.	5 mL/1 mL/ -	65%	65%	n.d.
2	2.0 eq.	5 mL/ - / -	66%	n.d.	66%
3	2.0 eq.	2.5 mL/1 mL/2.5 mL	64%	50%	14%
4	1.0 eq.	5 mL/1 mL/ -	60%	43%	15%

a) Determined by crude ^{19}F NMR (terminal CF_3) using BTF as an internal standard.

Table S4. Investigation of reaction condition for reductive reaction conditions.

2. [1] H, ^{13}C and ^{19}F NMR spectra

4a: ^{1}H NMR ($CDCl_3$, 500 MHz)

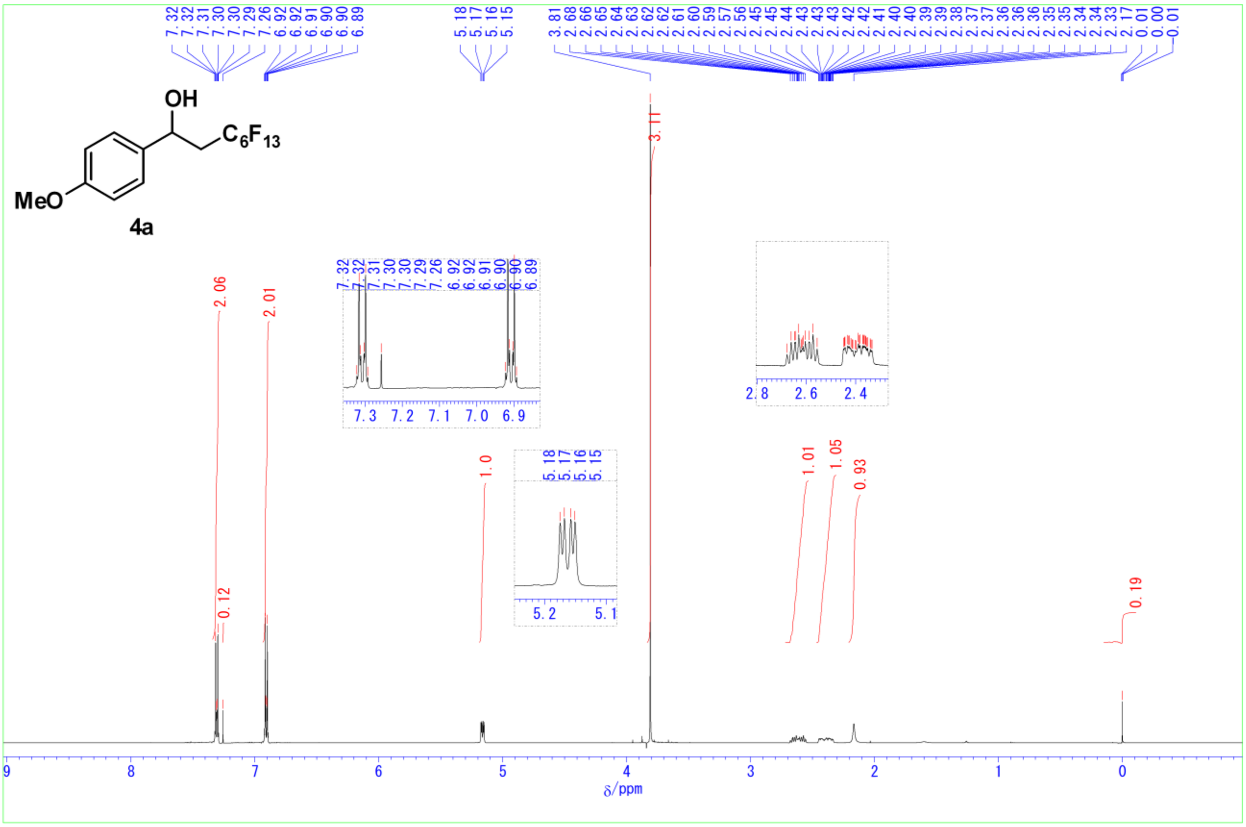

4a: ^{13}C NMR ($CDCl_3$ 151 MHz)

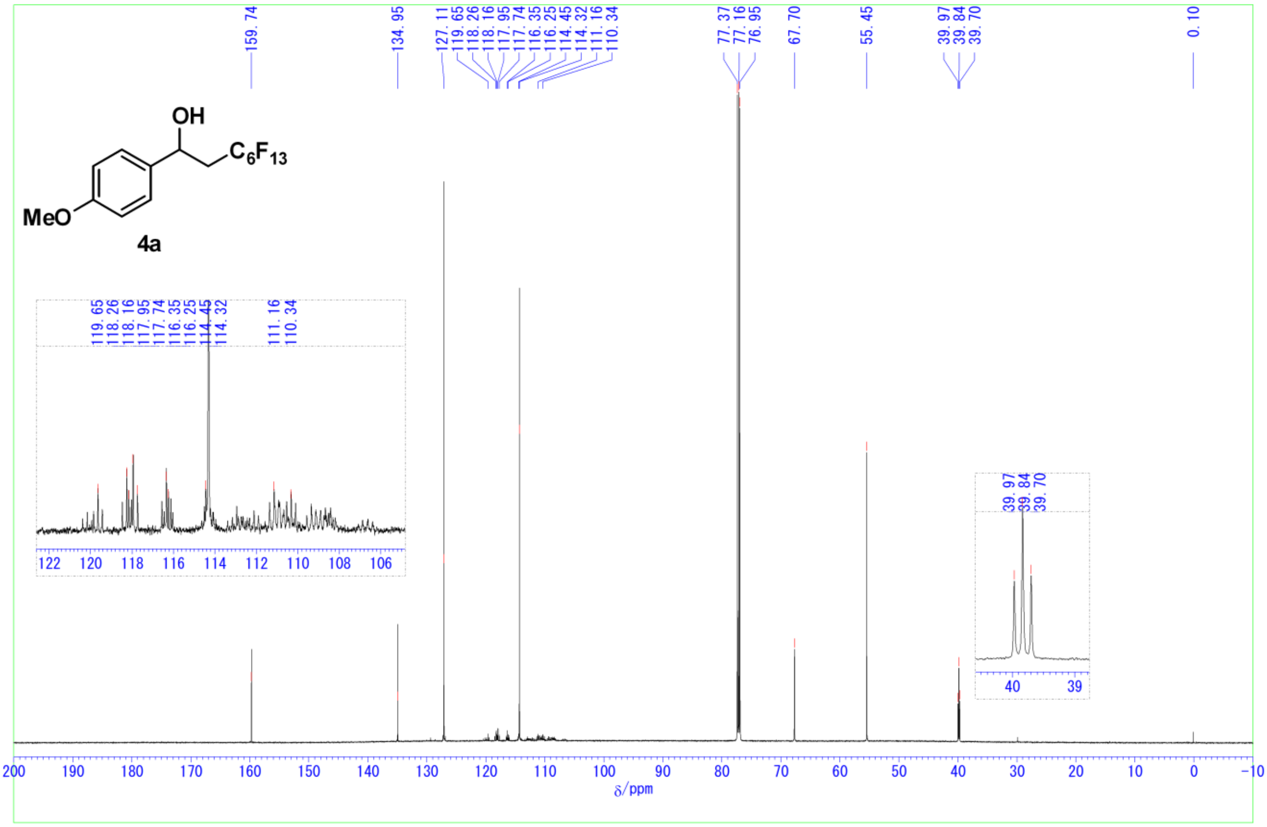

4a: ^{19}F NMR (CDCl$_3$, 471 MHz)

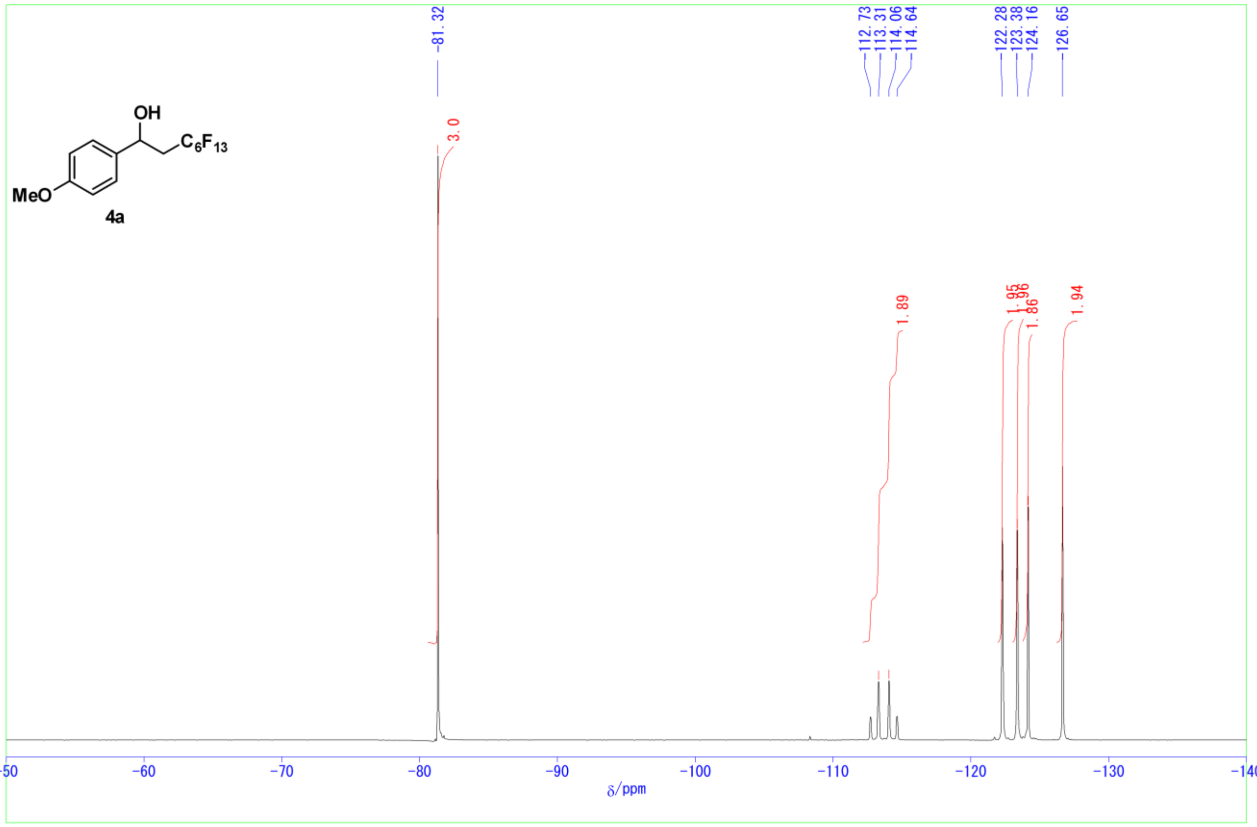

4aa: ^{1}H NMR (CDCl$_3$, 400 MHz)

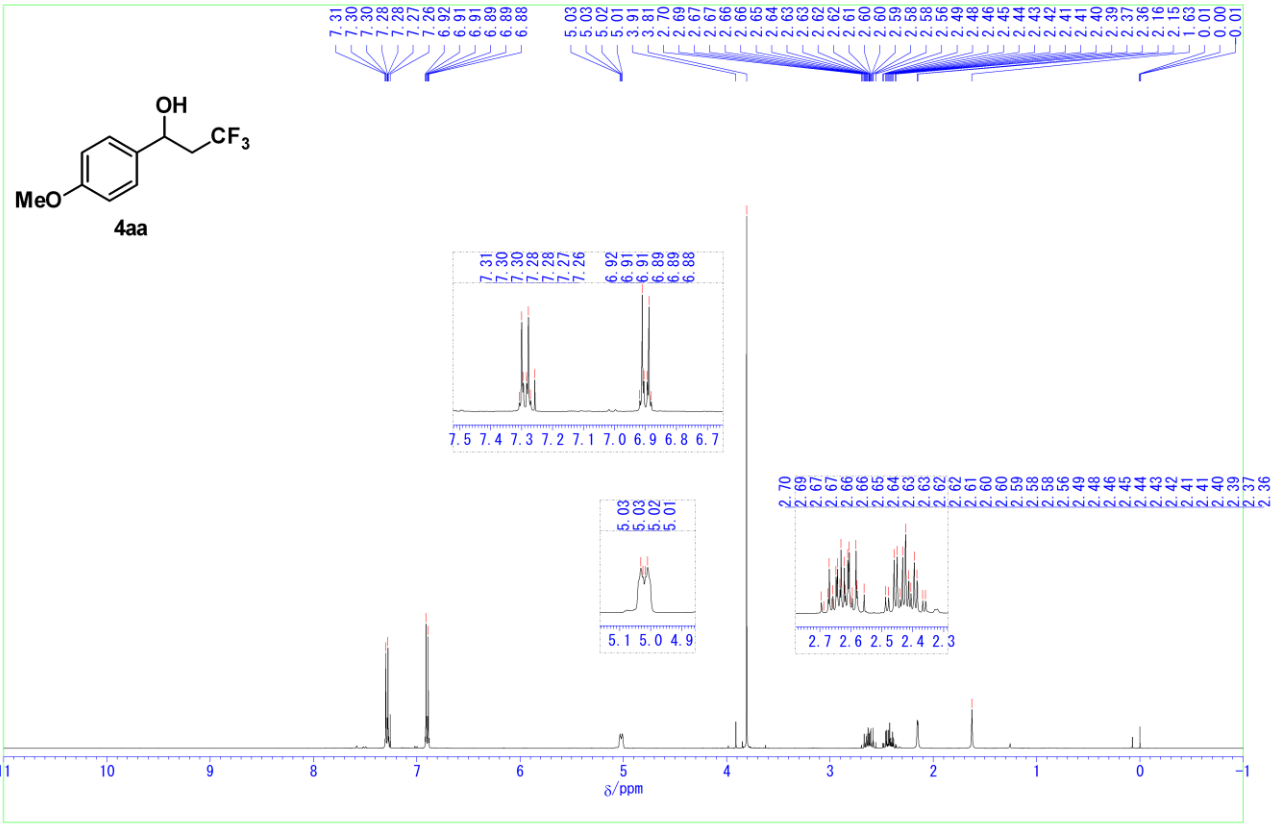

4aa: ^{13}C NMR ($CDCl_3$, 151 MHz)

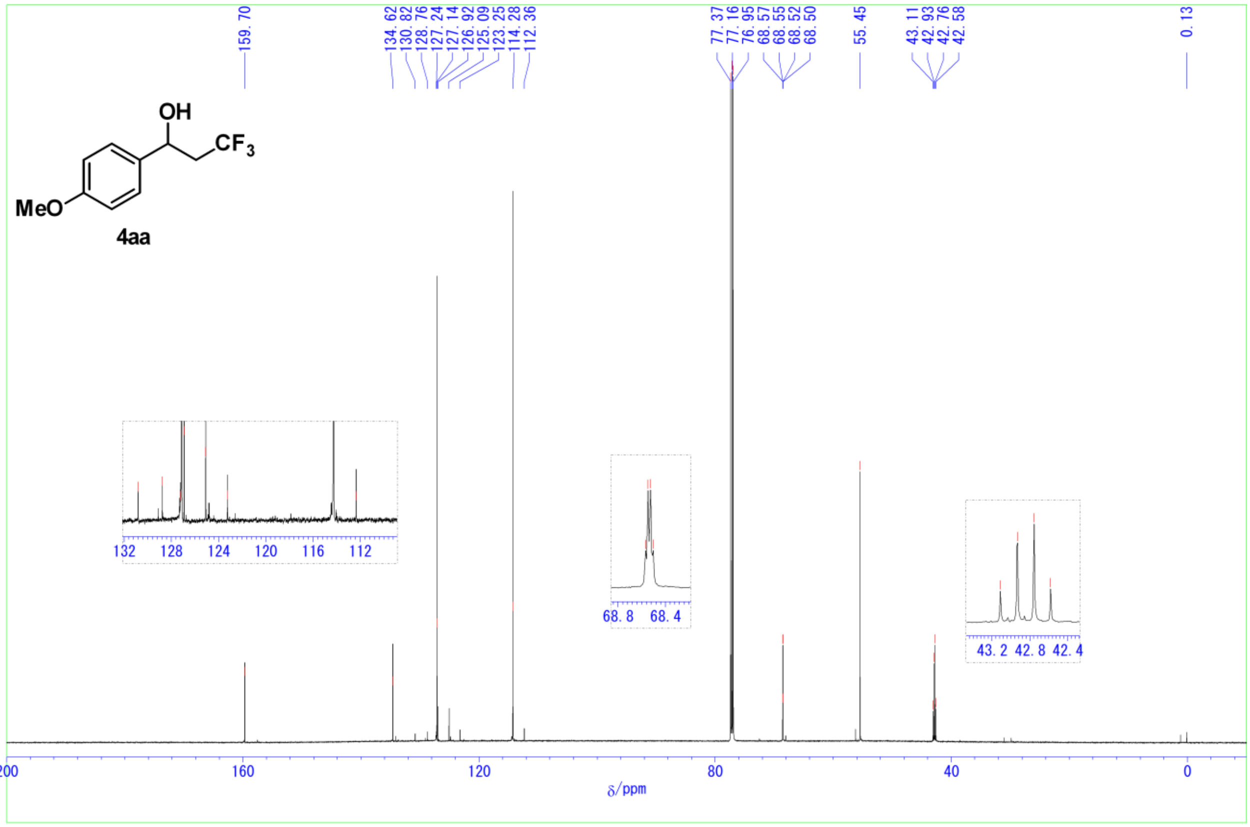

4aa: ^{19}F NMR ($CDCl_3$, 376 MHz)

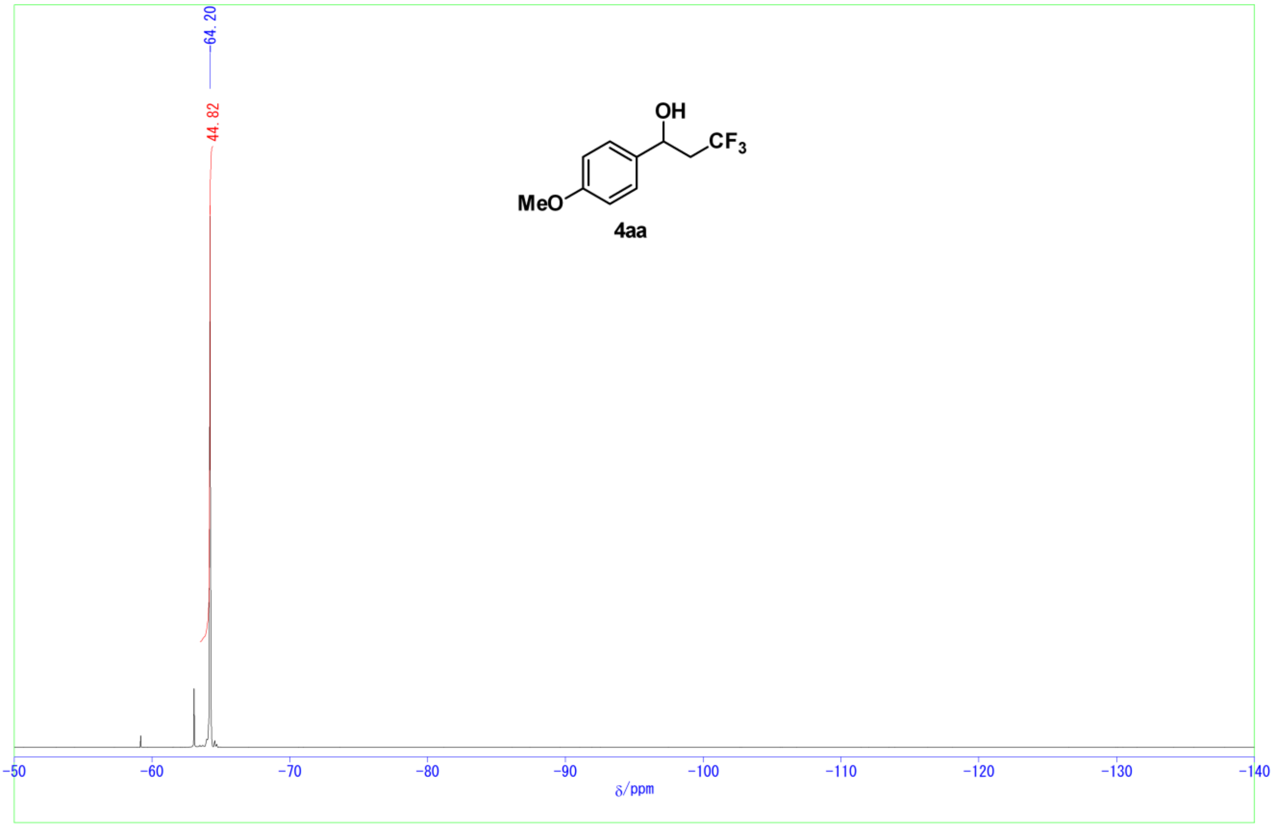

4ab: ^{1}H NMR ($CDCl_3$, 400 MHz)

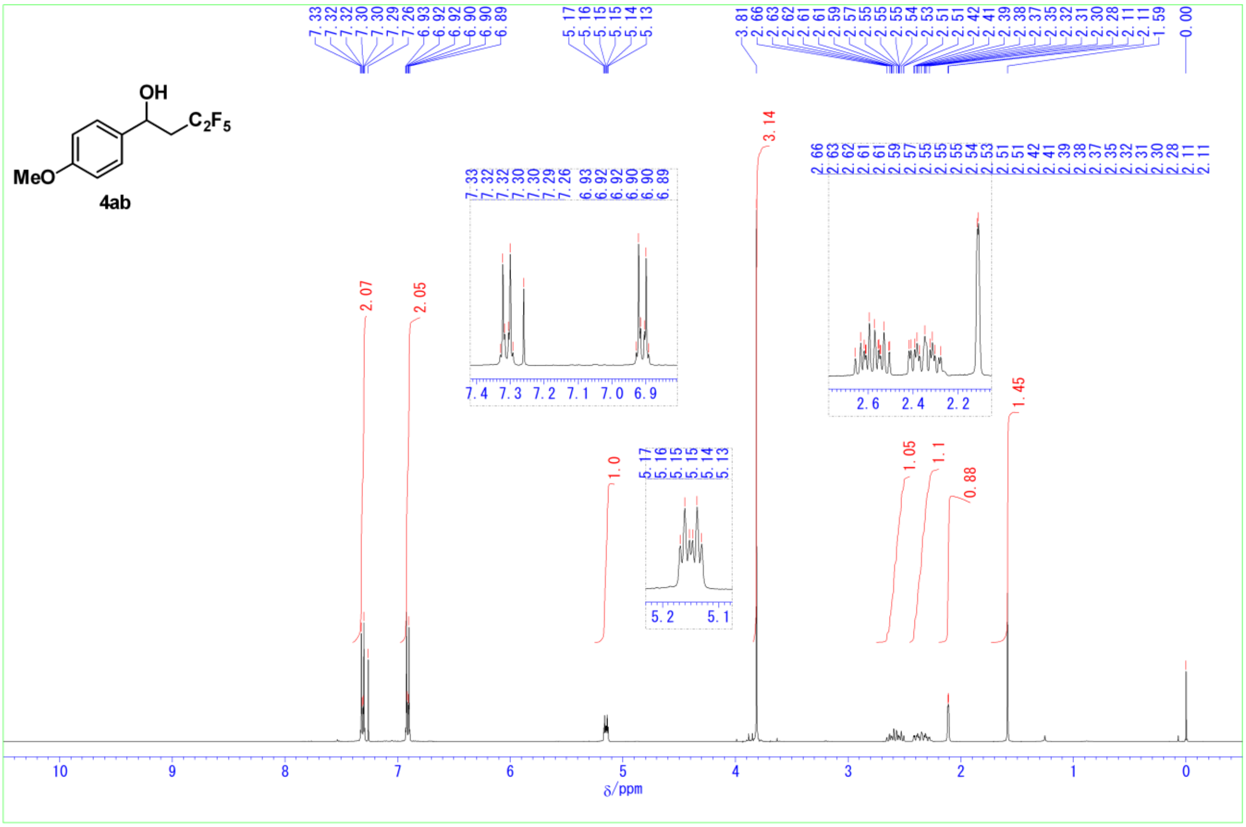

4ab: ^{13}C NMR ($CDCl_3$, 151 MHz)

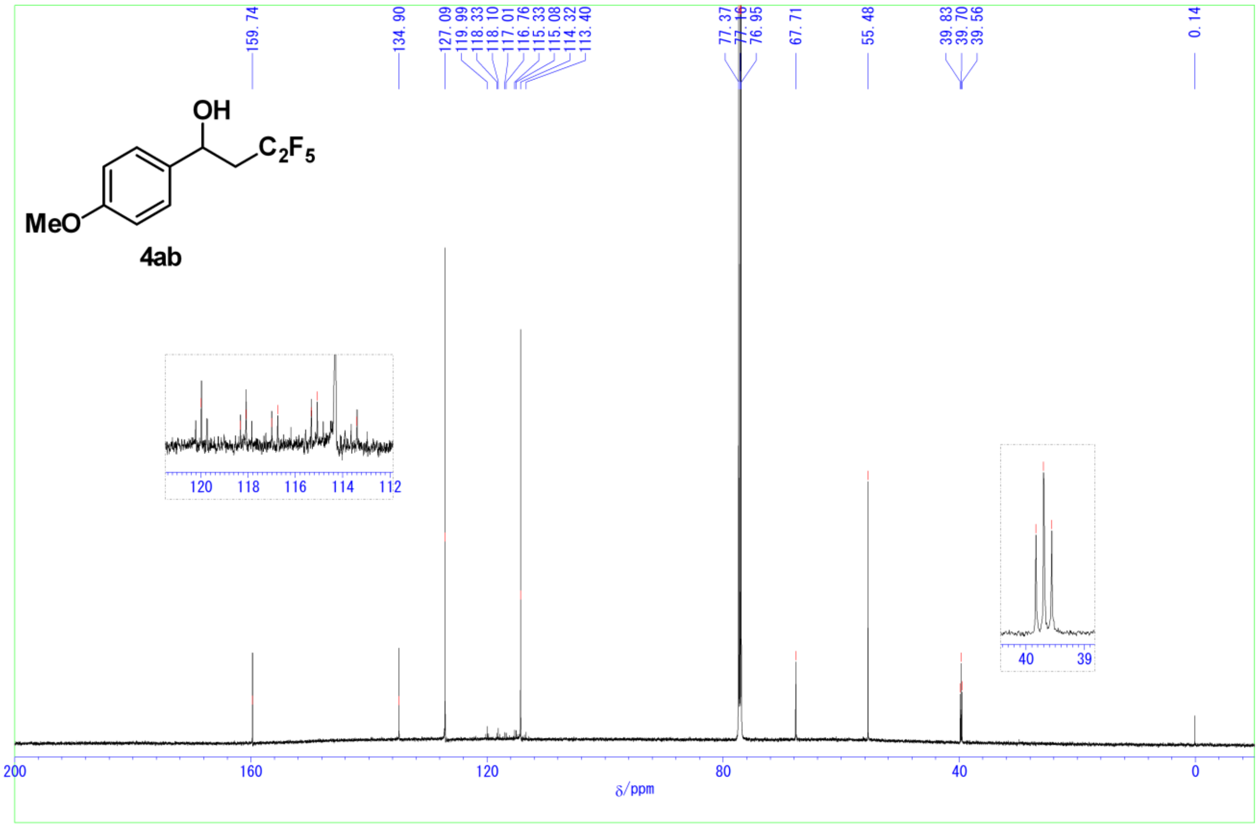

4ab: ^{19}F NMR ($CDCl_3$, 376 MHz)

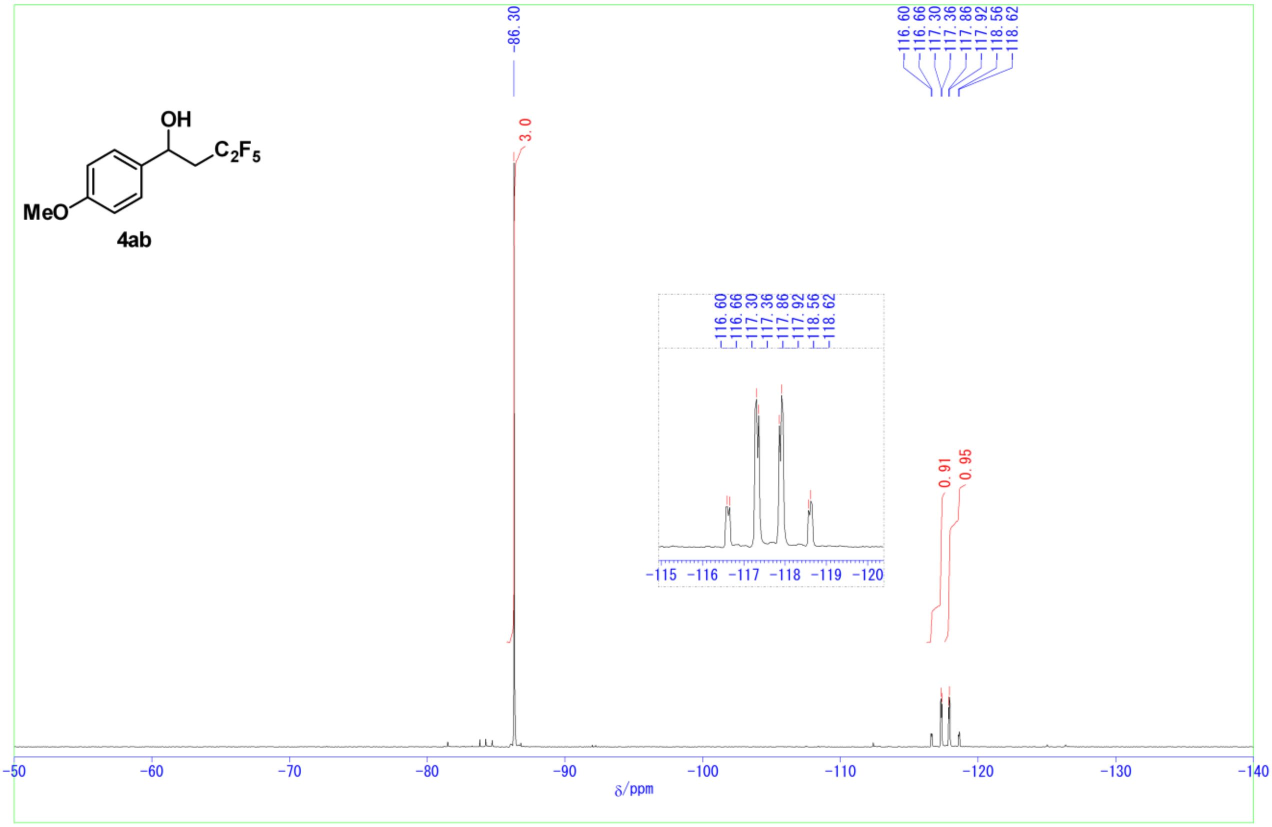

4ac: ^{1}H NMR ($CDCl_3$, 500 MHz)

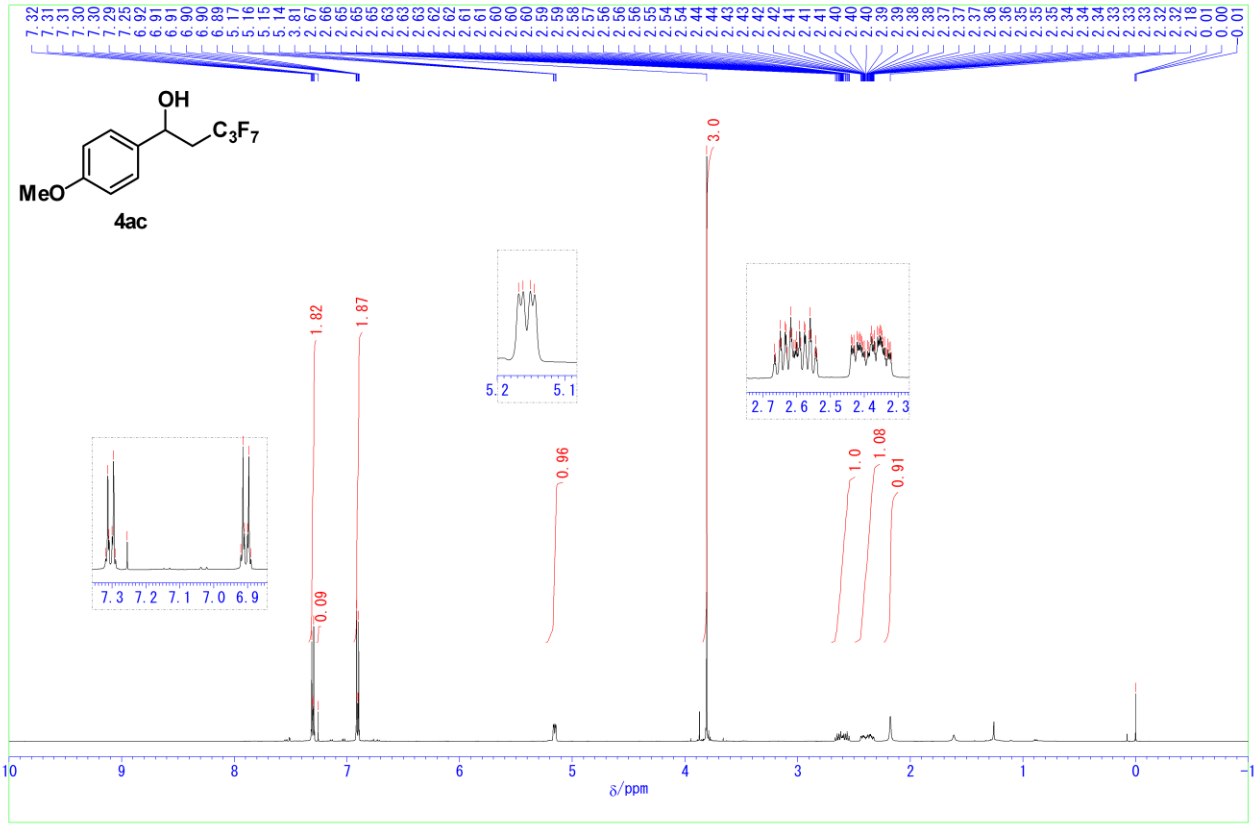

4ac: ^{13}C NMR (CDCl$_3$, 151 MHz)

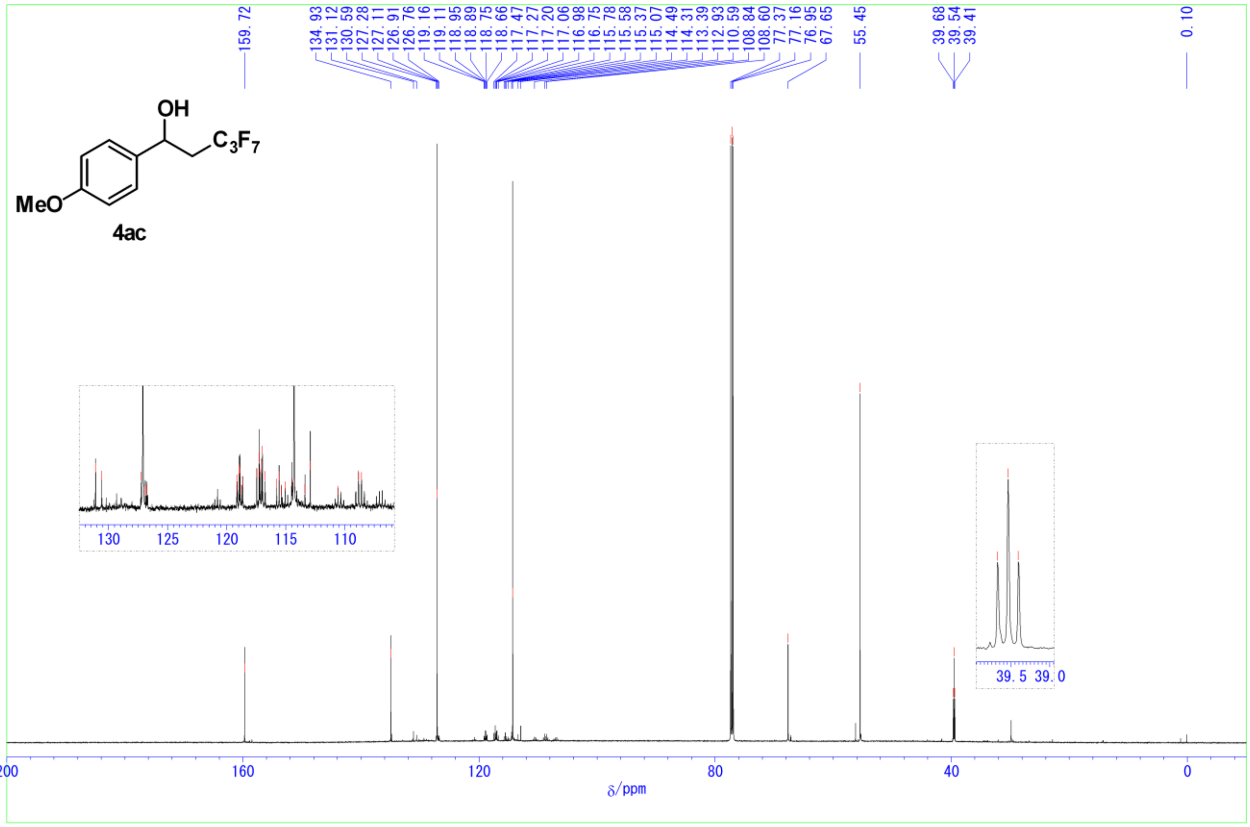

4ac: ^{19}F NMR (CDCl$_3$, 471 MHz)

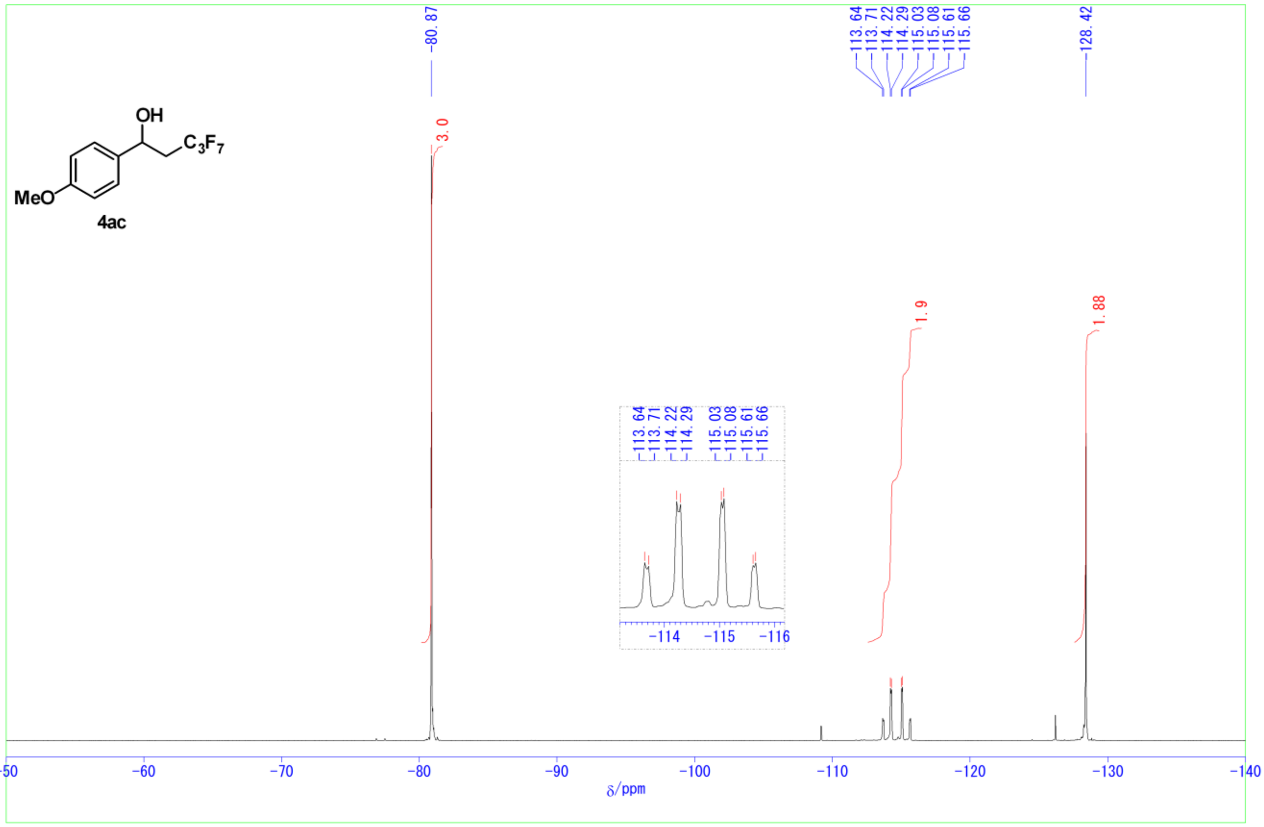

4ad: ^{1}H NMR ($CDCl_3$, 500 MHz)

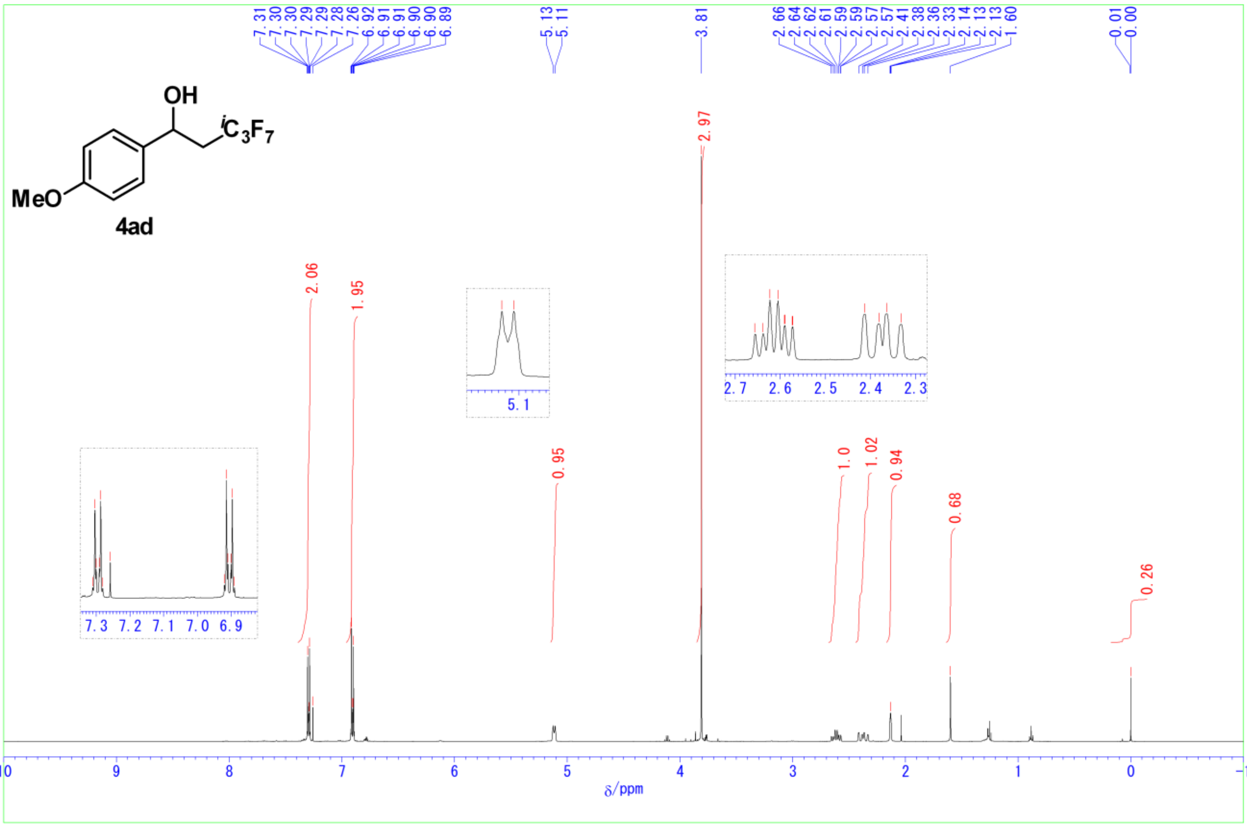

4ad: ^{13}C NMR ($CDCl_3$, 151 MHz)

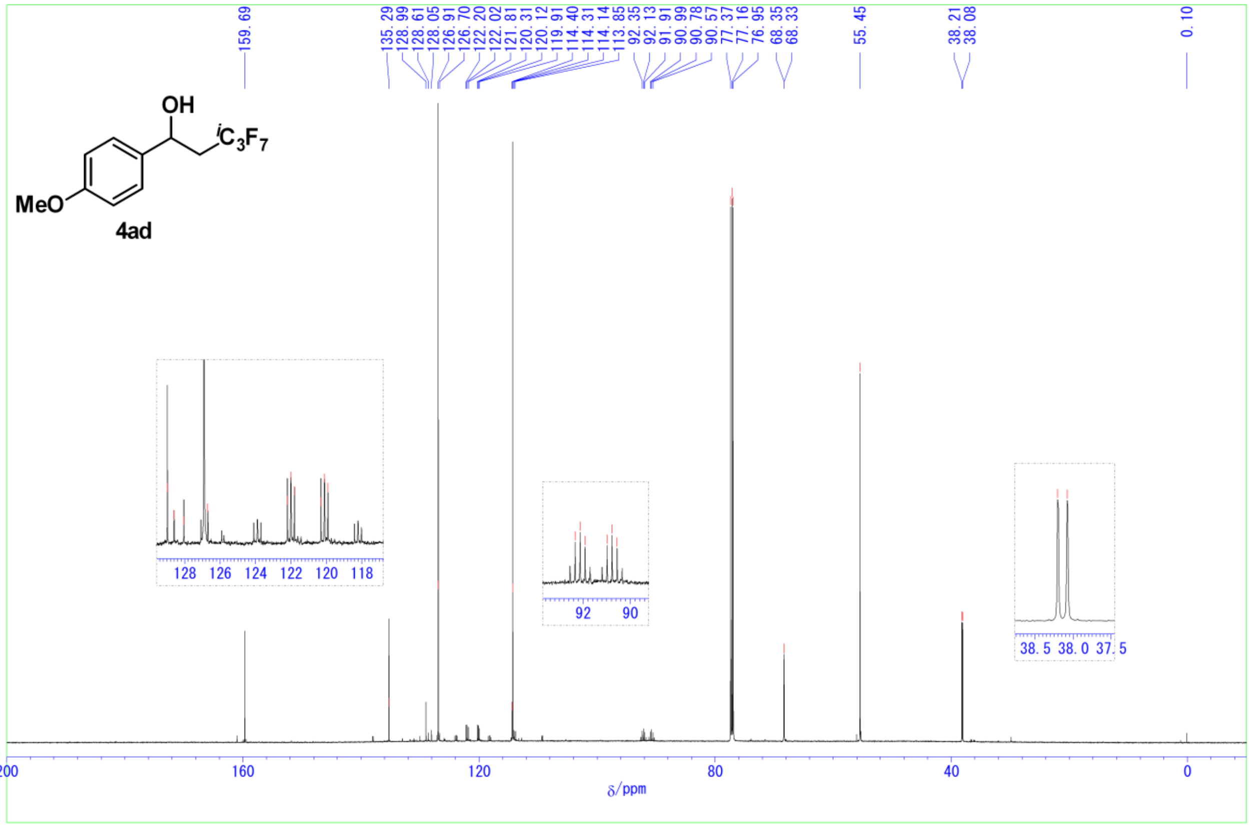

4ad: ^{19}F NMR ($CDCl_3$, 471 MHz)

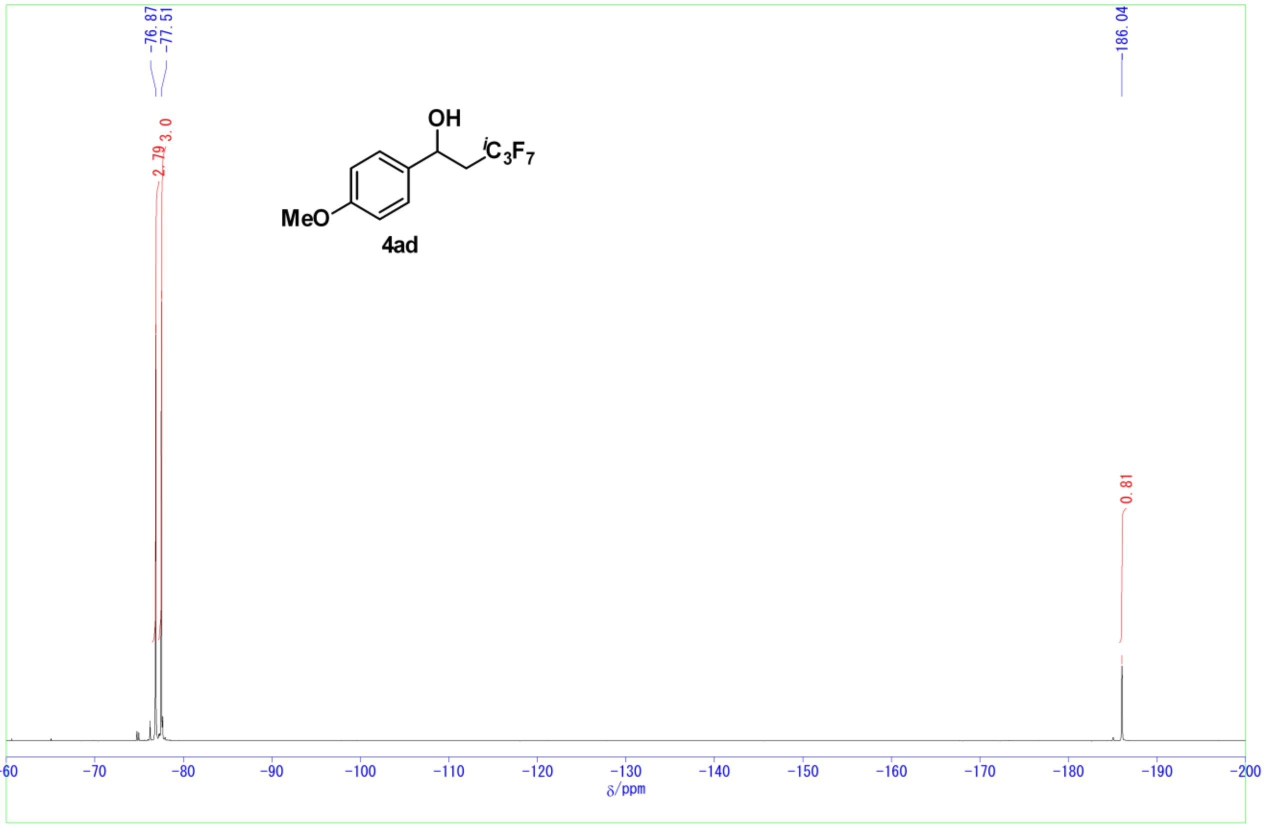

4ae: ^{1}H NMR ($CDCl_3$, 500 MHz)

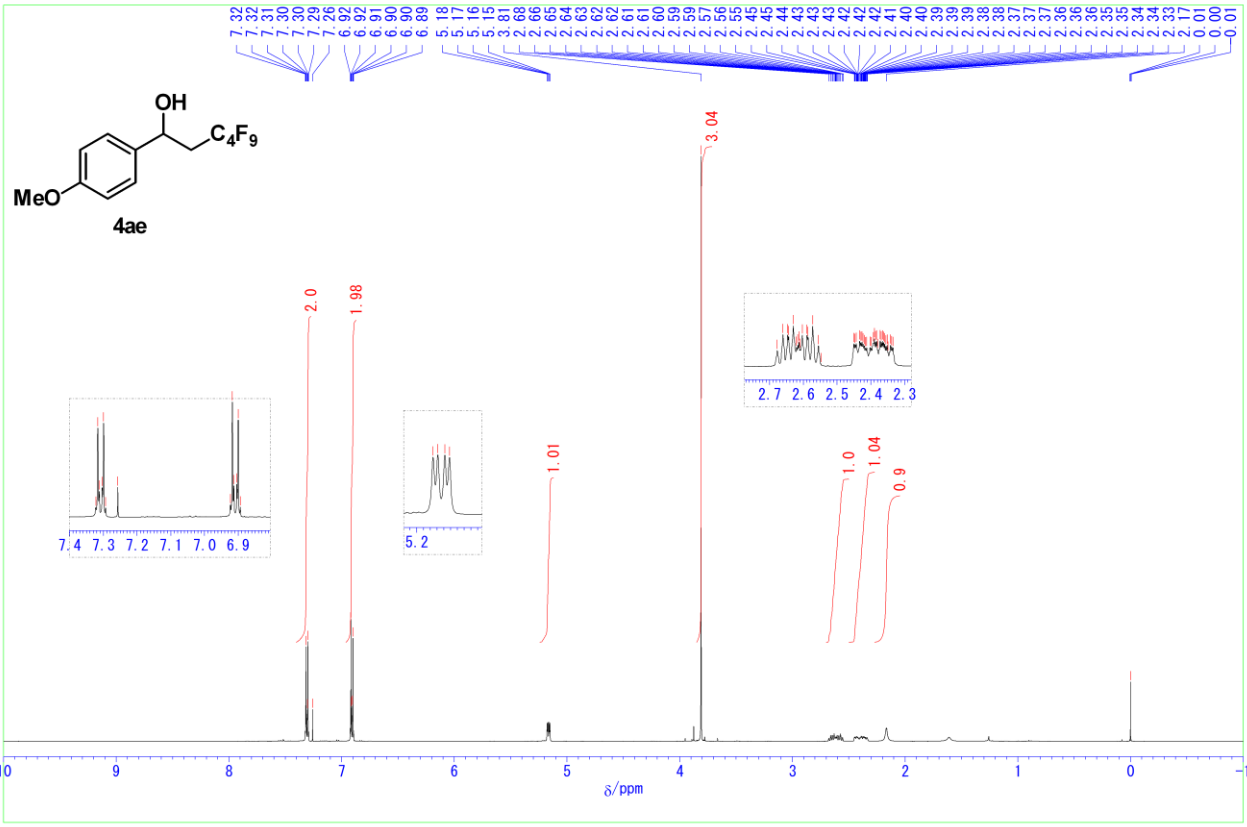

4ae: ^{13}C NMR (CDCl$_3$, 151 MHz)

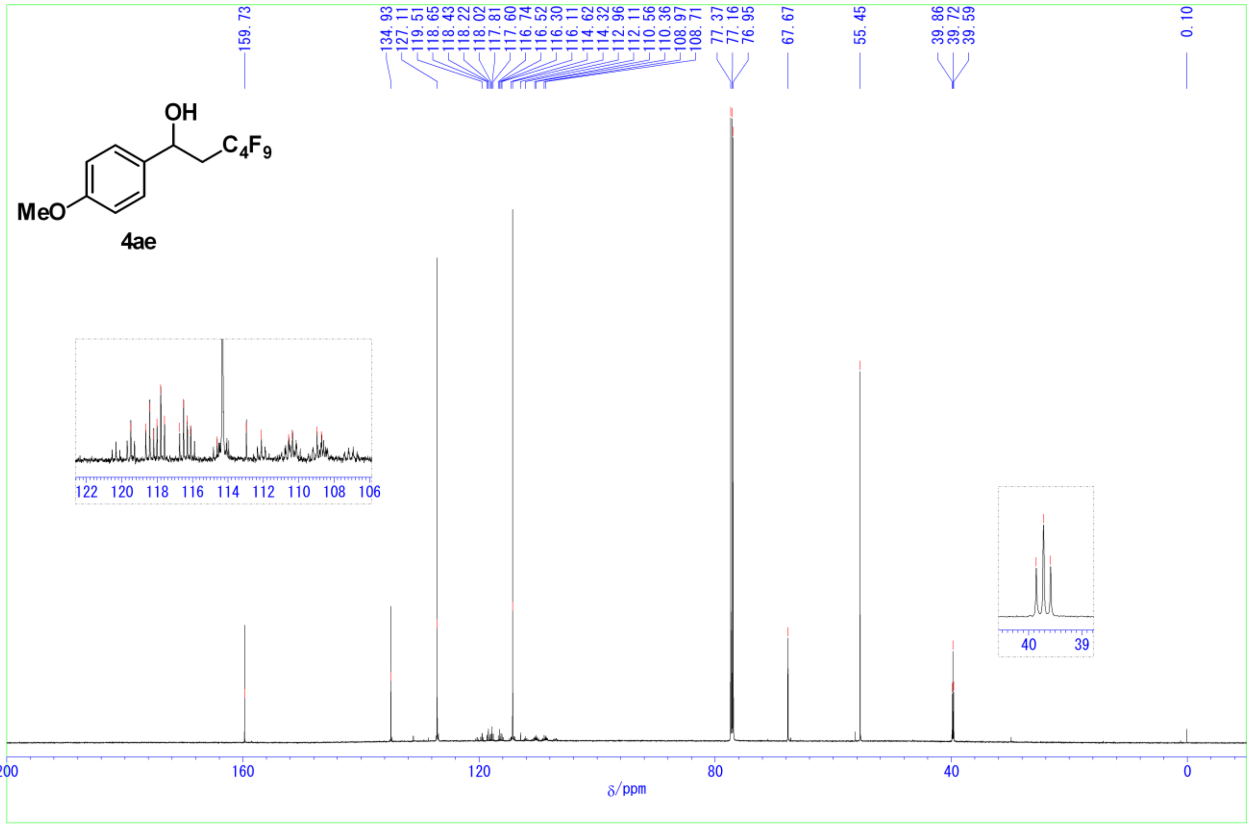

4ae: ^{19}F NMR (CDCl$_3$, 471 MHz)

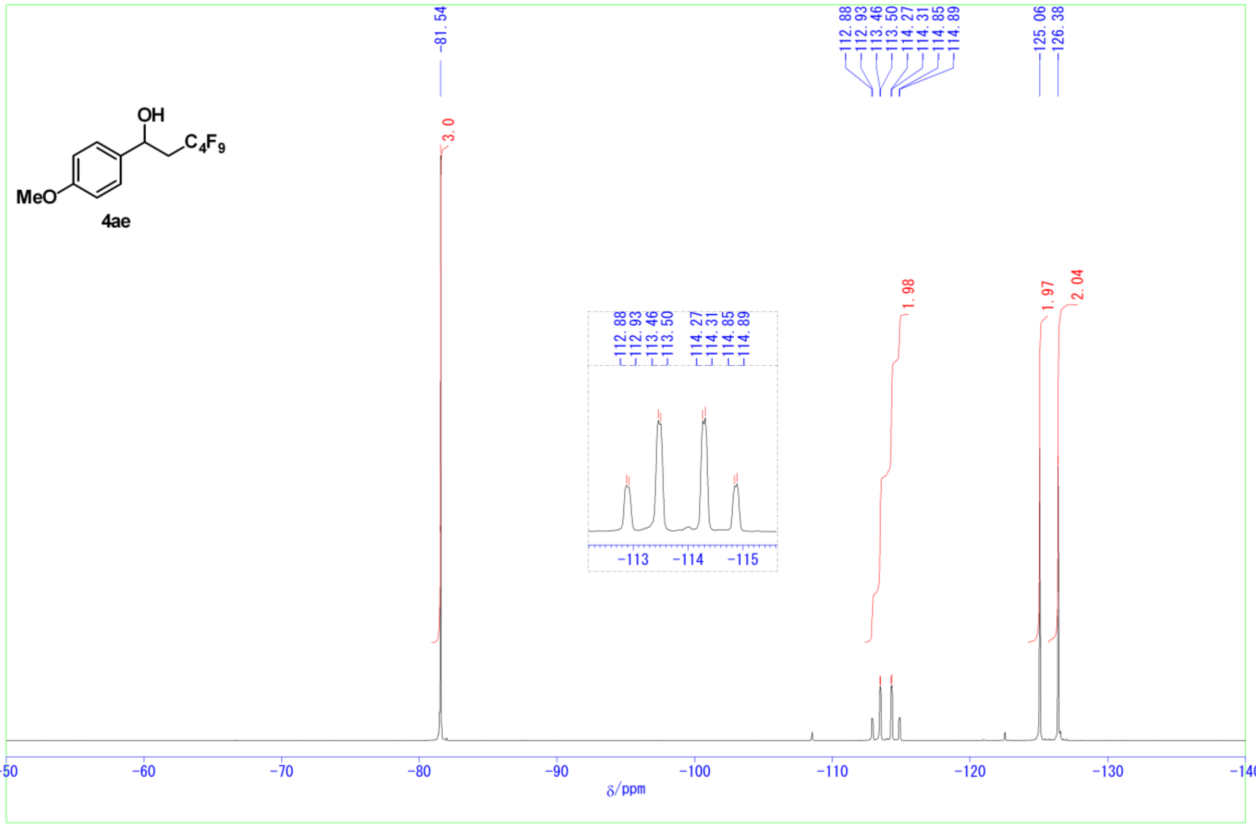

4af: ^{1}H NMR ($CDCl_3$, 500 MHz)

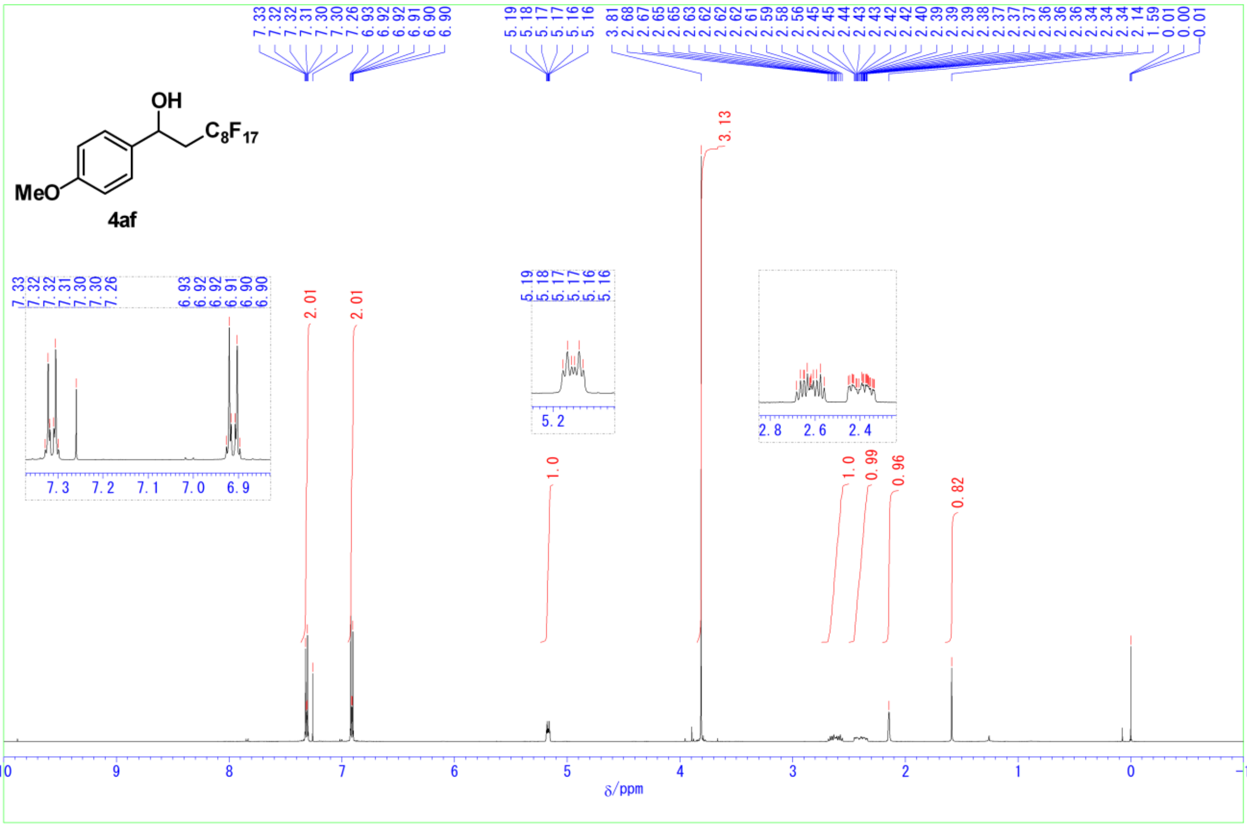

4af: ^{13}C NMR ($CDCl_3$, 151 MHz)

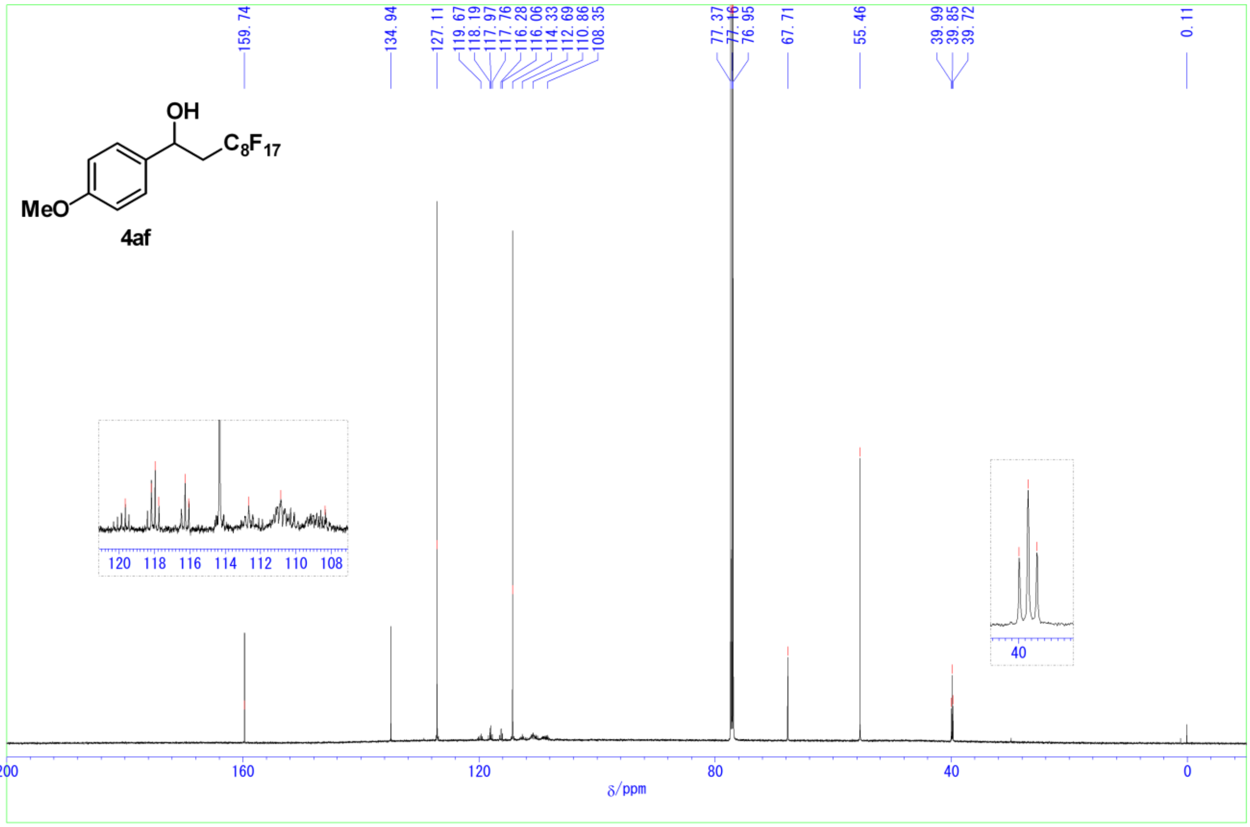

4af: ^{19}F NMR (CDCl$_3$, 471 MHz)

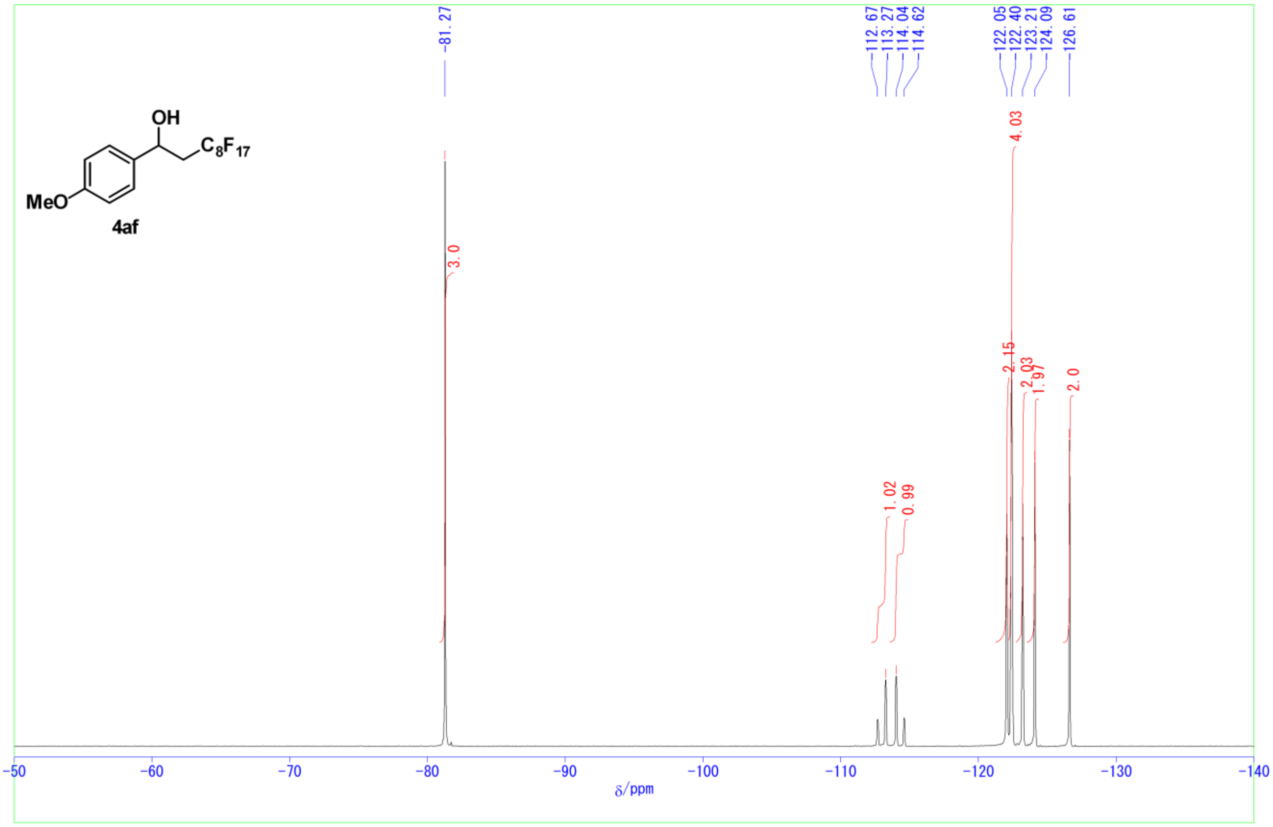

4c: ^{1}H NMR (CDCl$_3$, 400 MHz)

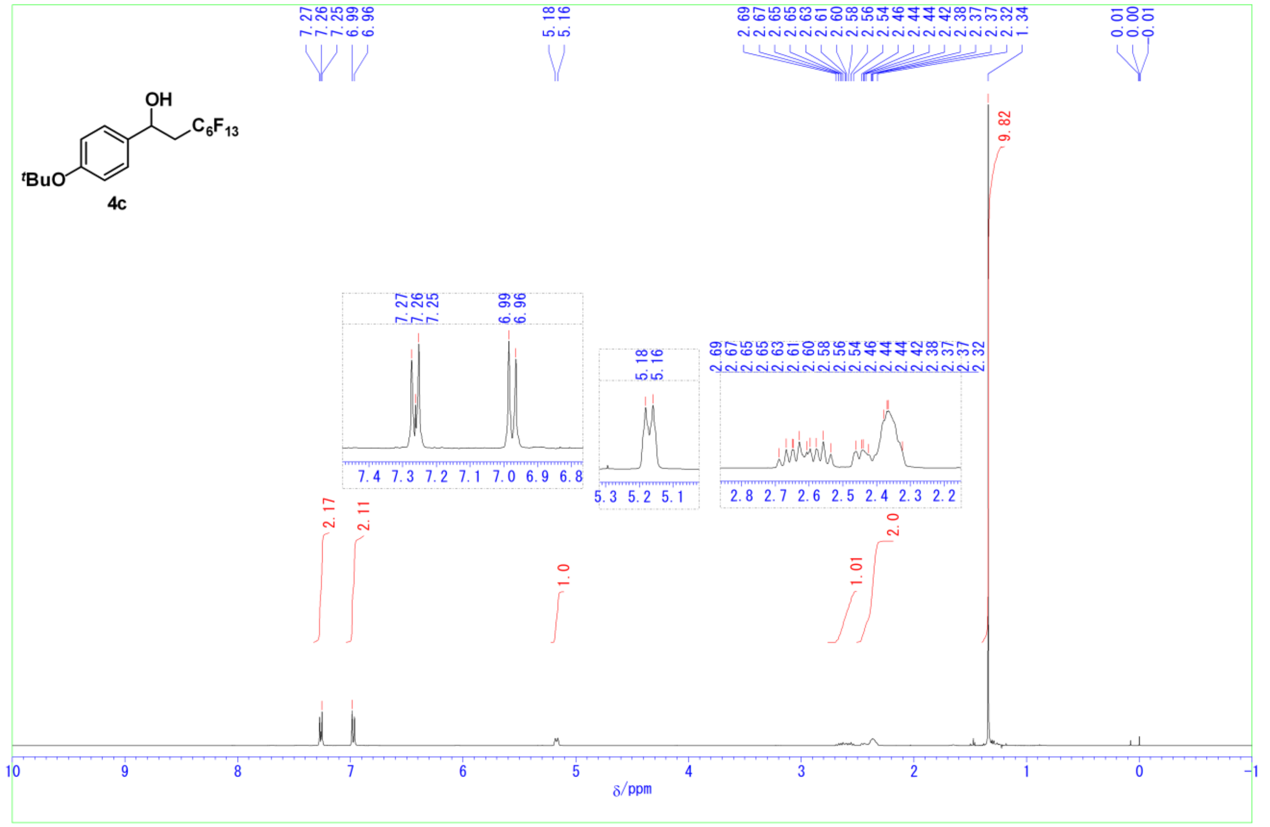

4c: ^{13}C NMR (CDCl$_3$, 151 MHz)

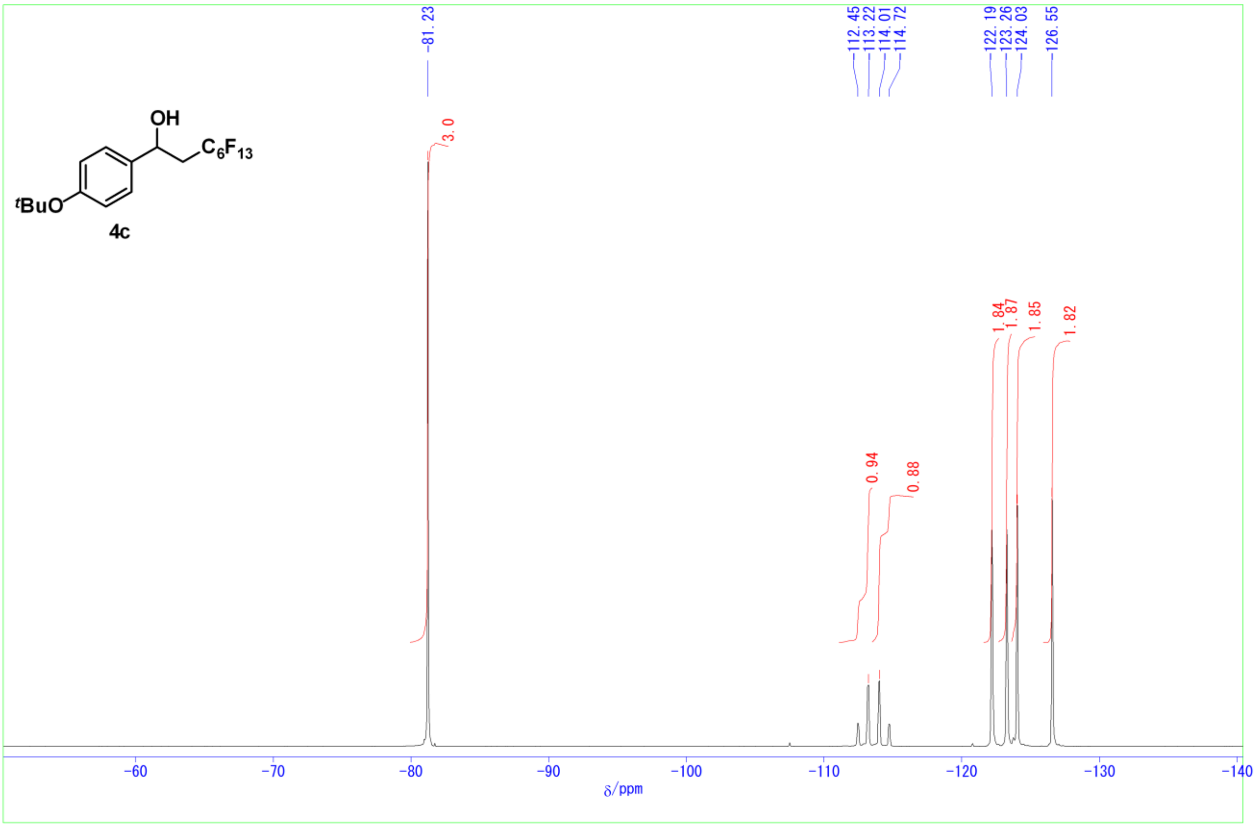

4c: ^{19}F NMR (CDCl$_3$, 376 MHz)

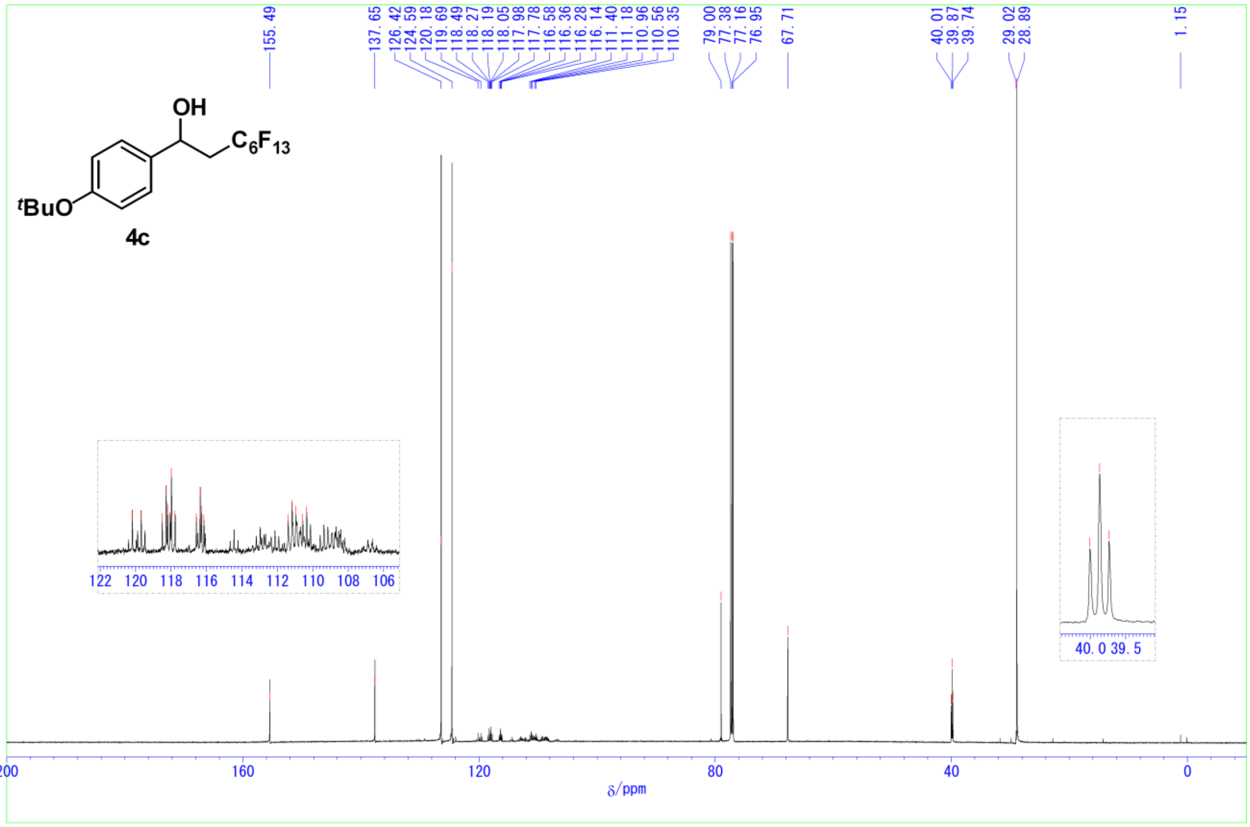

4d: ^{1}H NMR ($CDCl_3$, 500 MHz)

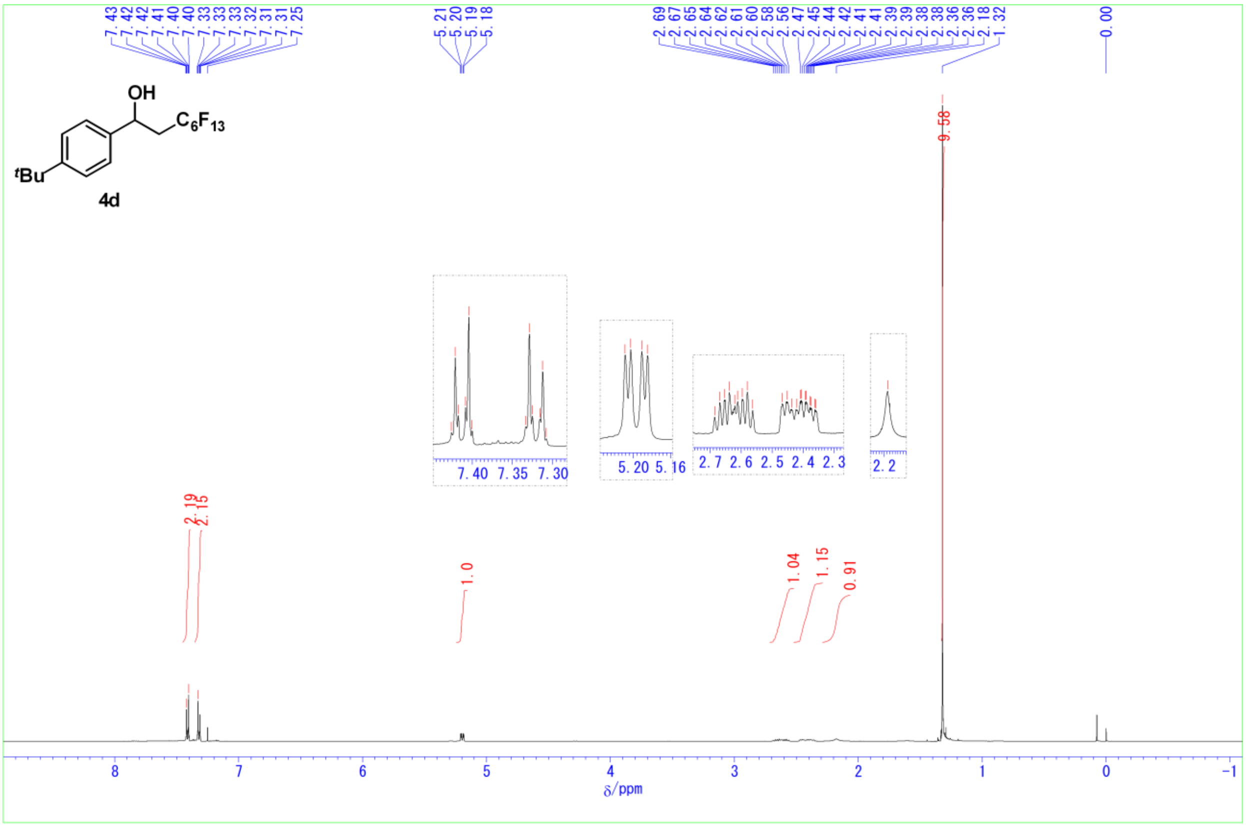

4d: ^{13}C NMR ($CDCl_3$, 151 MHz)

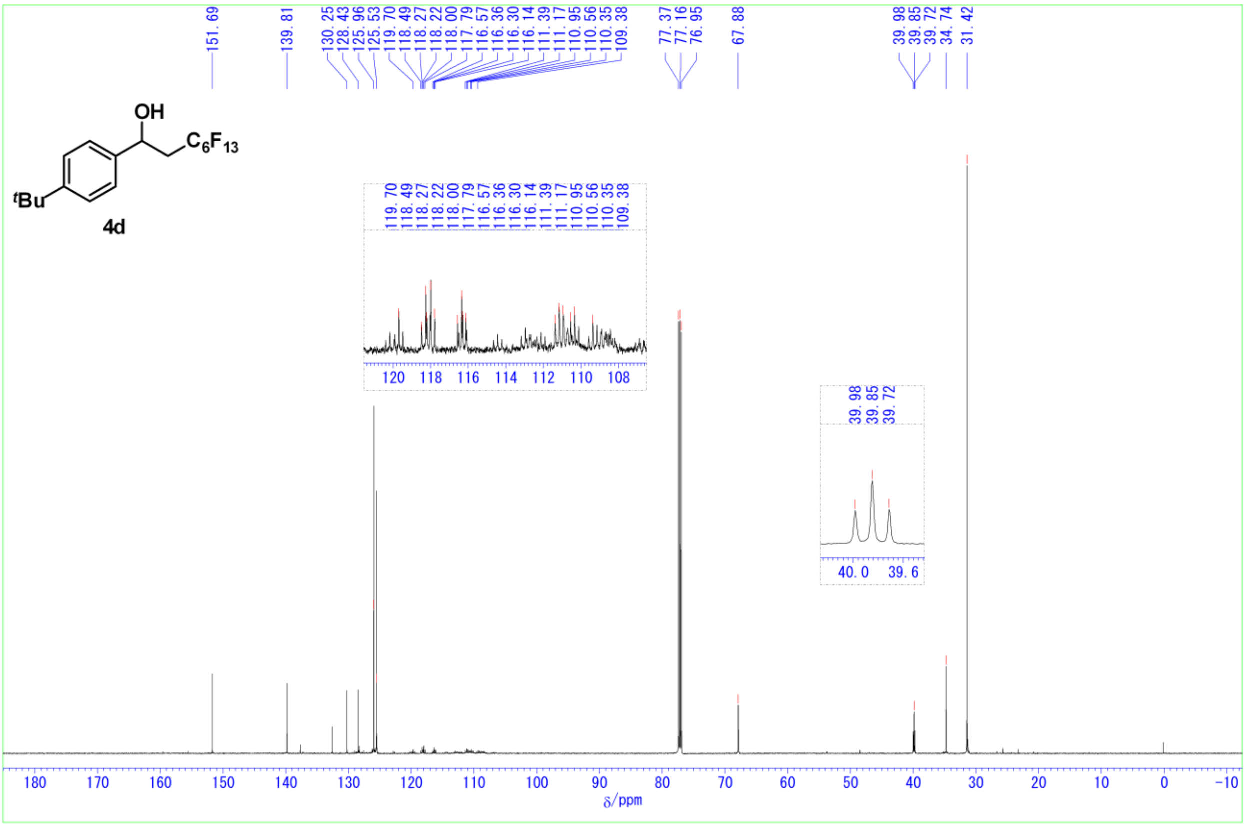

4d: ^{19}F NMR ($CDCl_3$, 471 MHz)

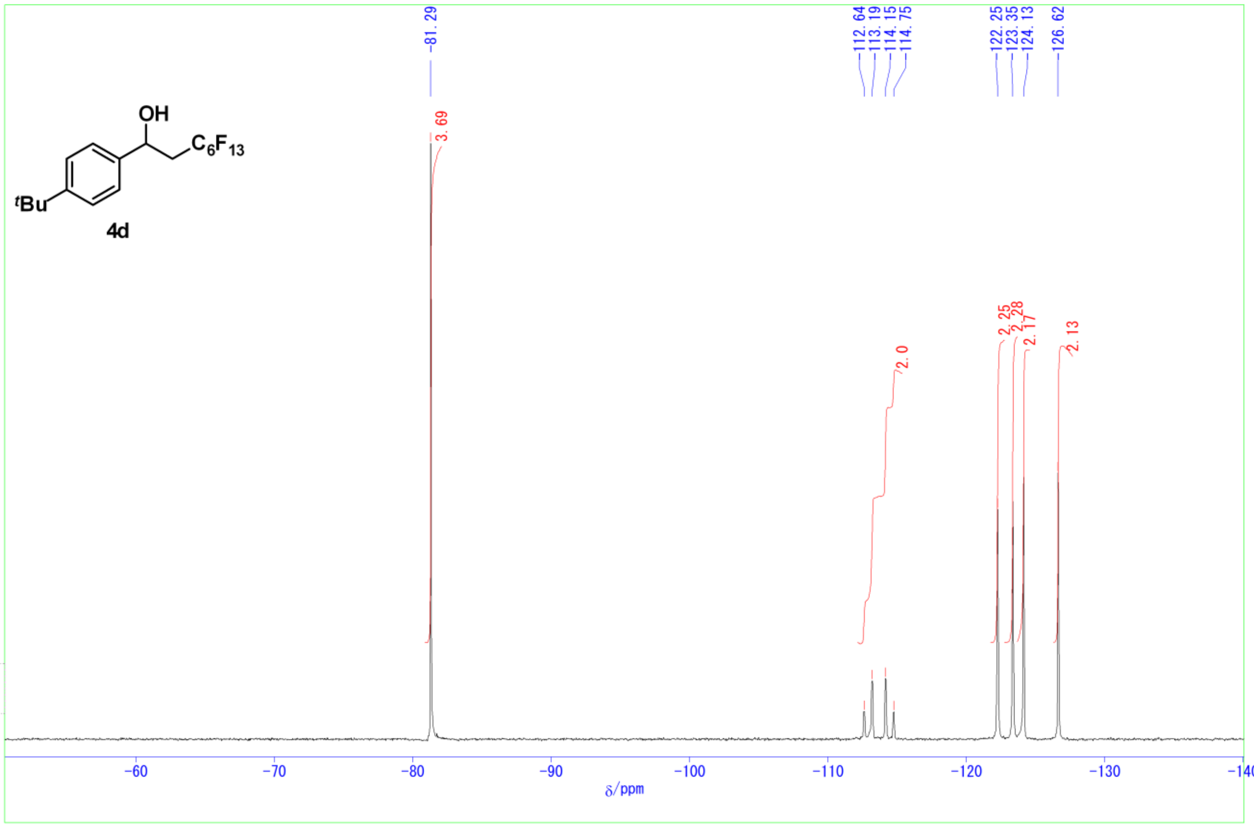

4e: ^{1}H NMR ($CDCl_3$, 400 MHz)

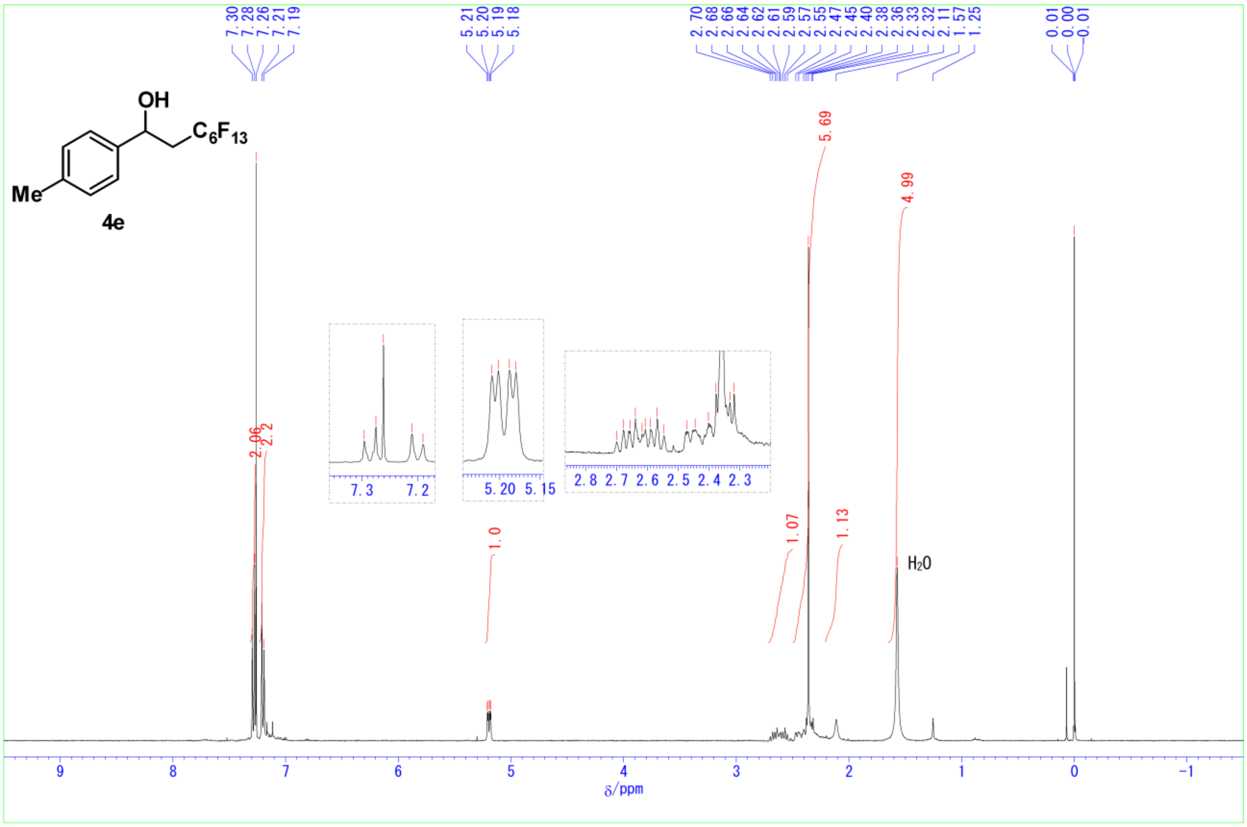

4e: ^{13}C NMR ($CDCl_3$, 151 MHz)

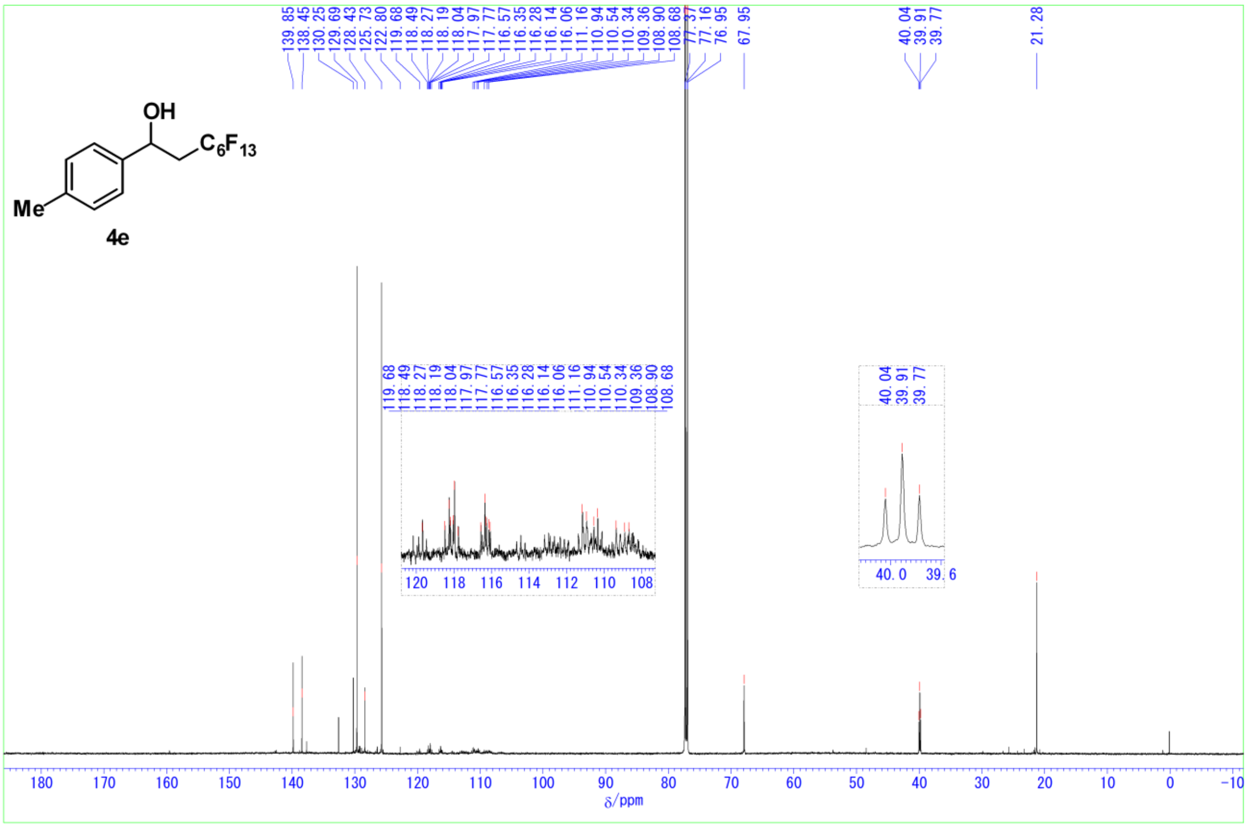

4e: ^{19}F NMR ($CDCl_3$, 376 MHz)

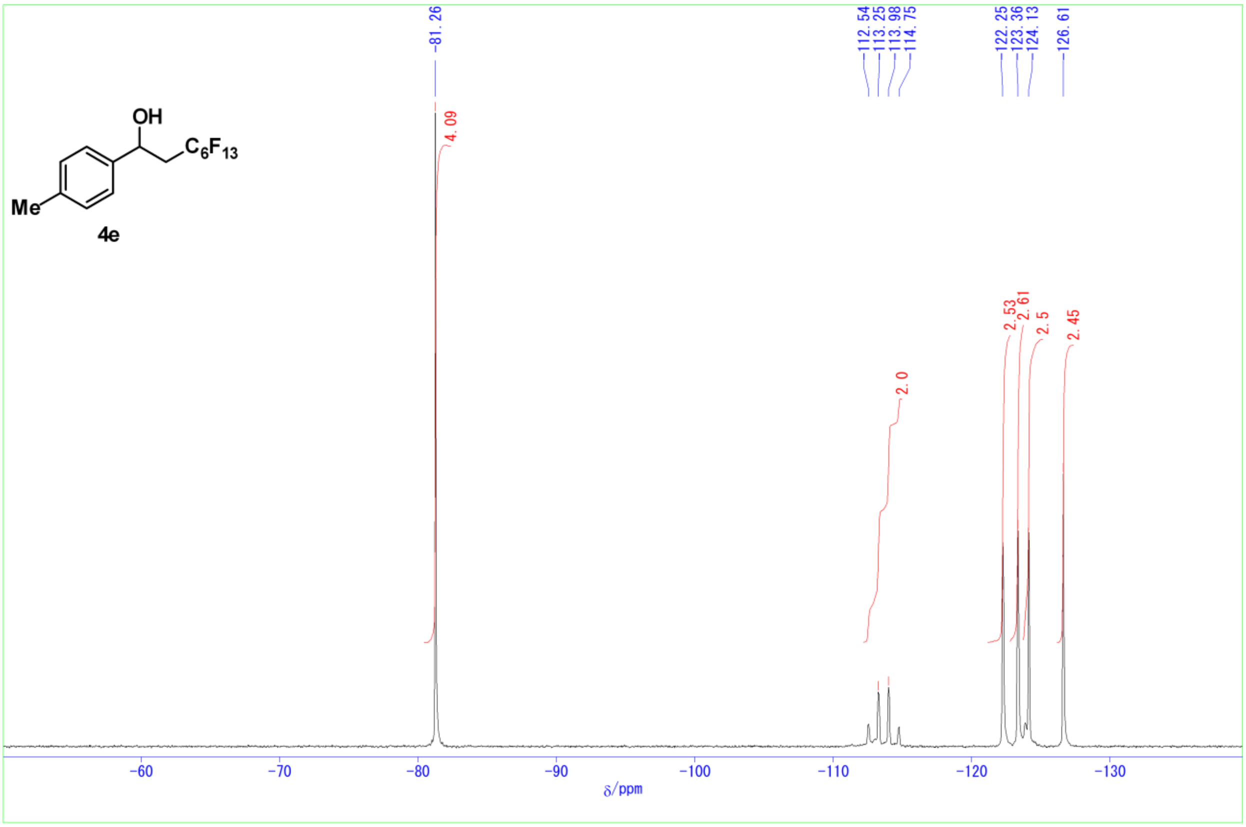

4f: ^{1}H NMR ($CDCl_3$, 500 MHz)

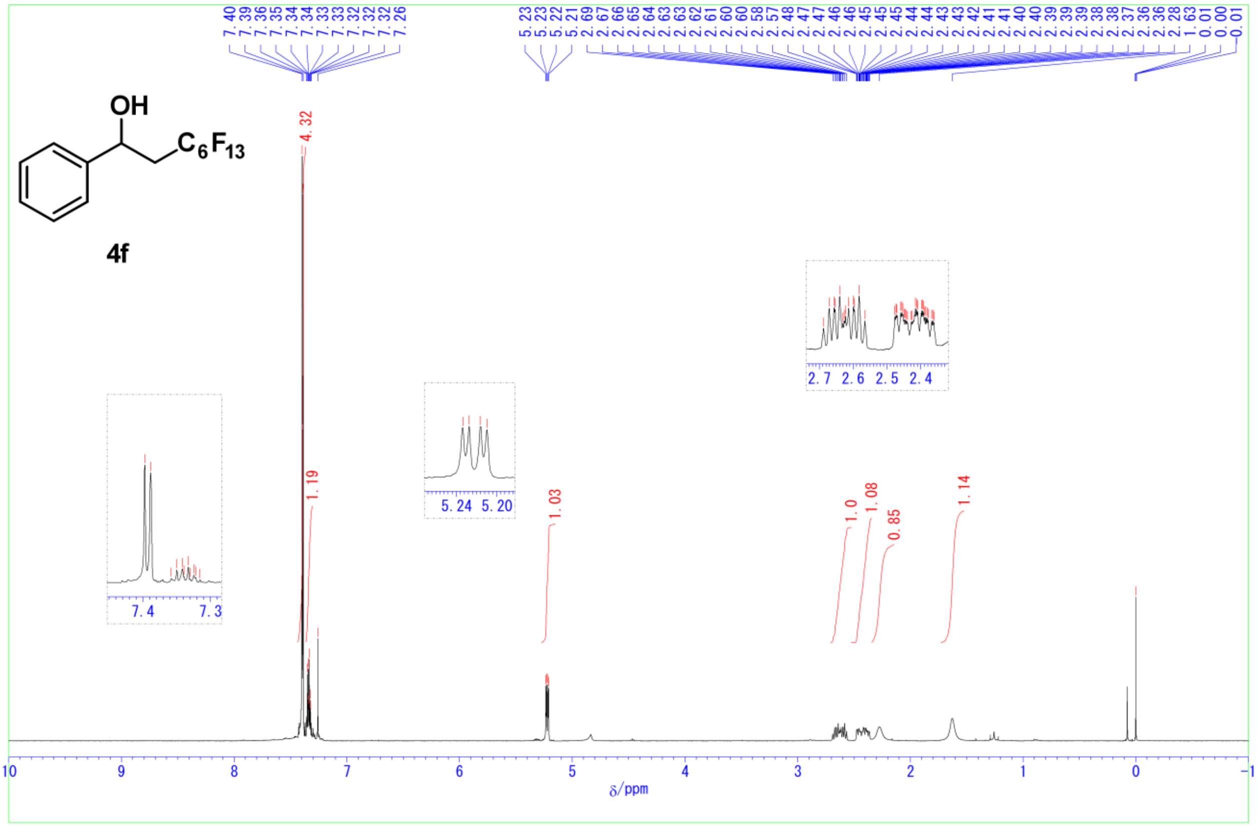

4f: ^{13}C NMR ($CDCl_3$, 151 MHz)

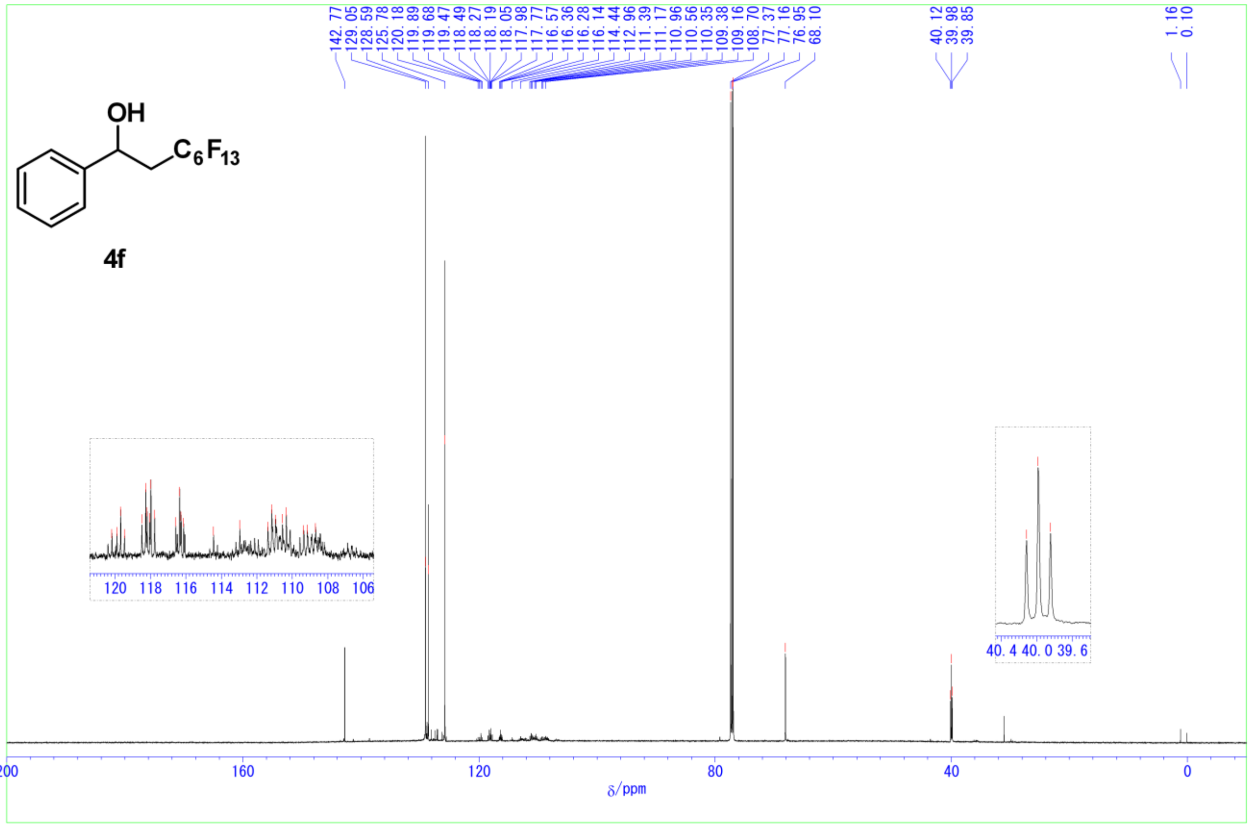

4f: ^{19}F NMR ($CDCl_3$, 471 MHz)

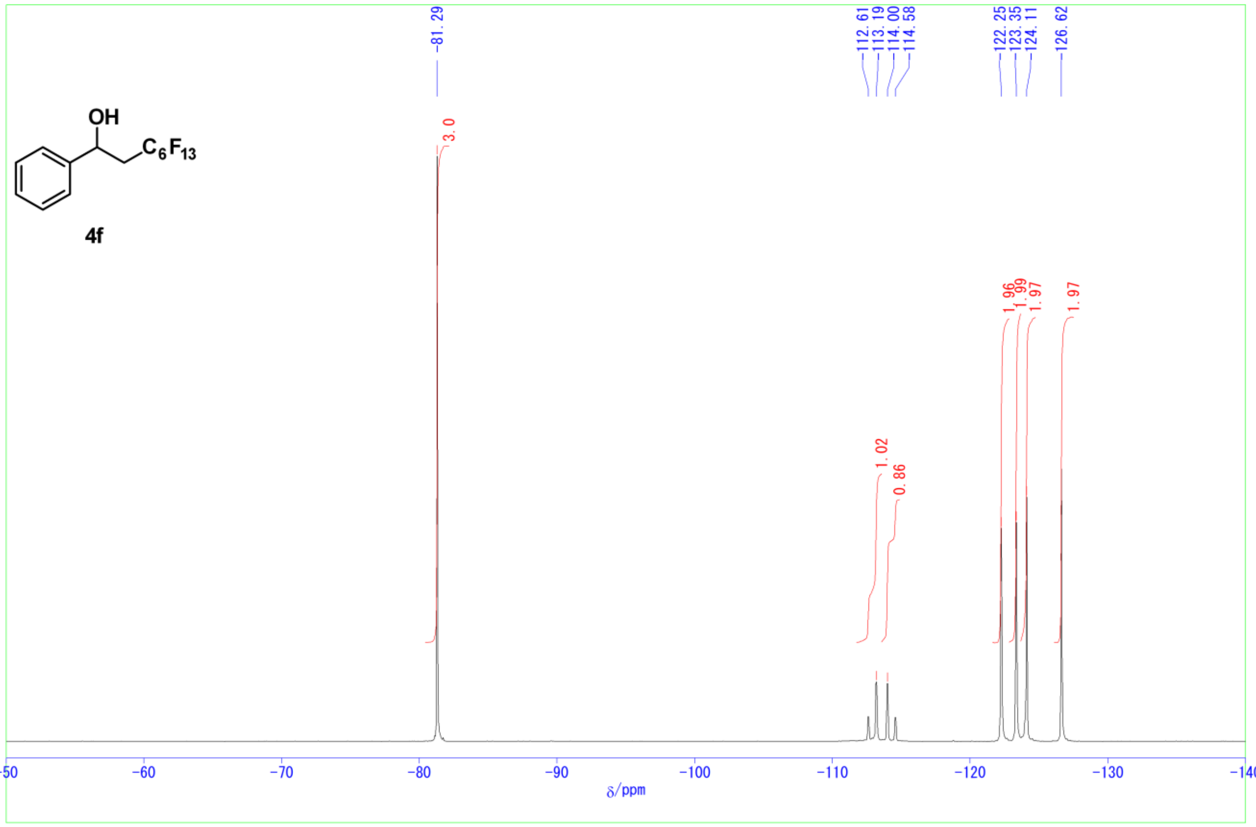

4g: ^{1}H NMR ($CDCl_3$, 500 MHz)

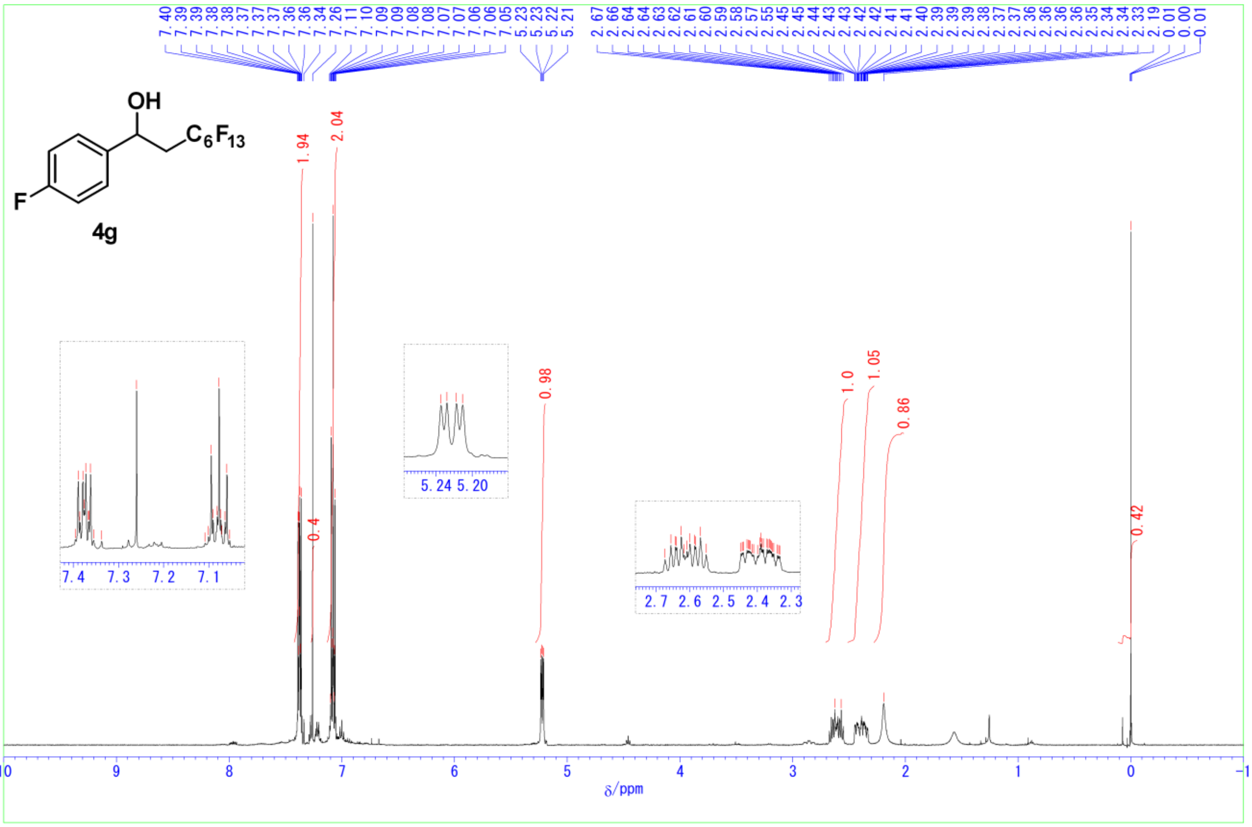

4g: ^{13}C NMR (CDCl$_3$, 151 MHz)

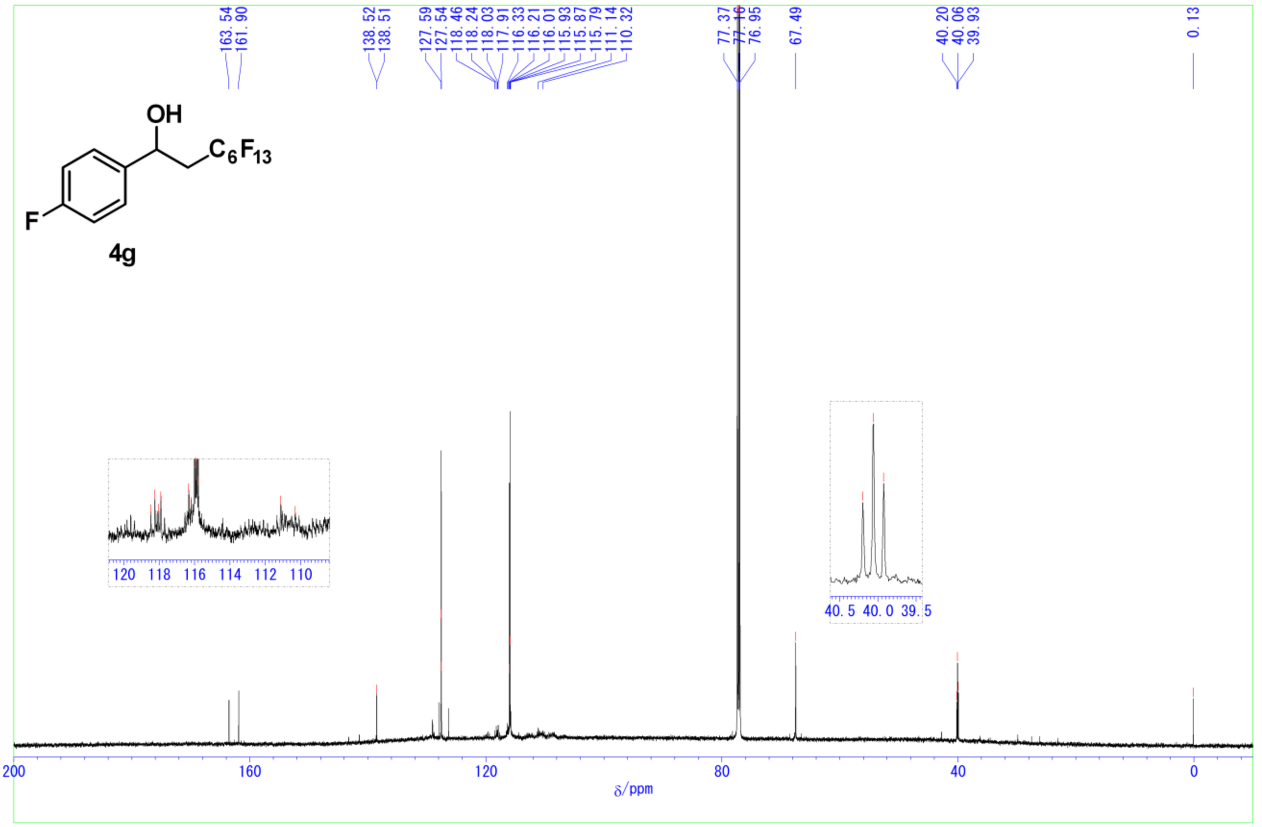

4g: ^{19}F NMR (CDCl$_3$, 471 MHz)

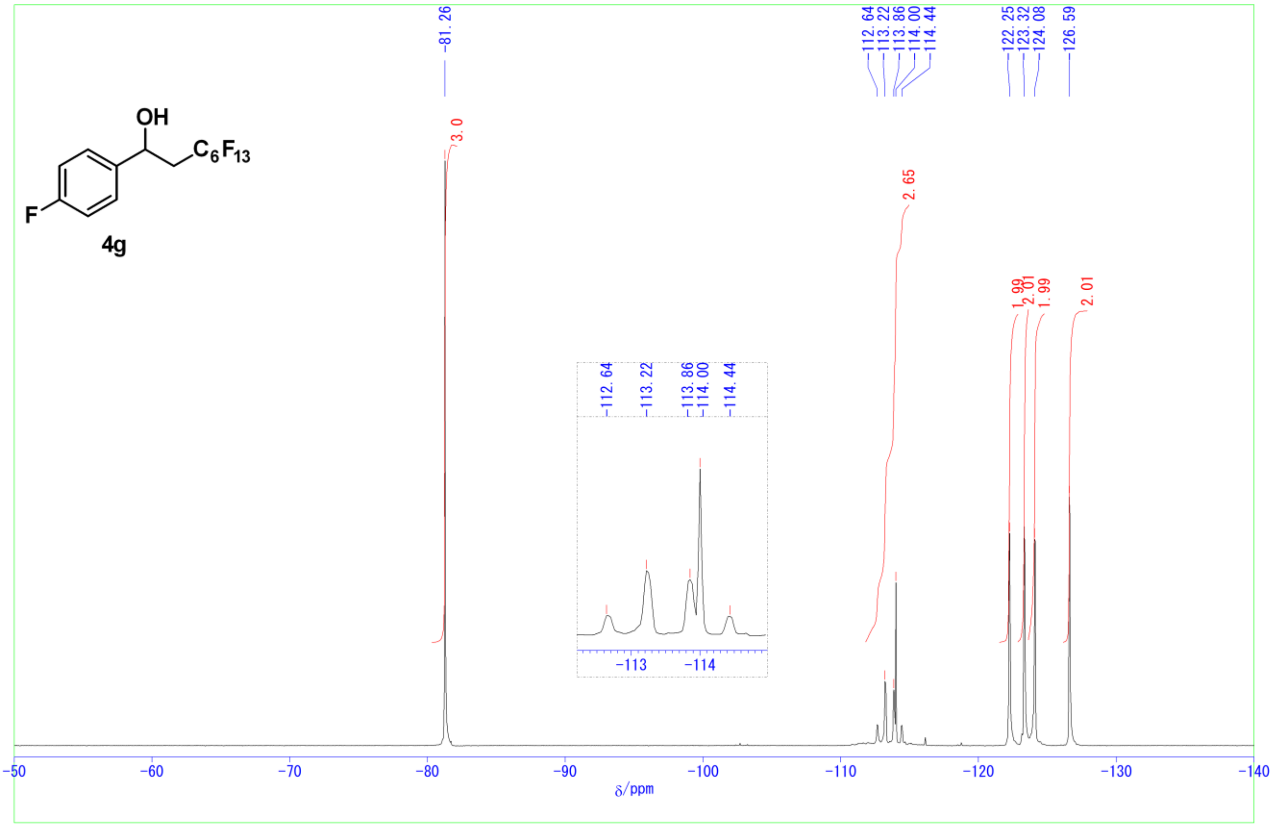

4h: ^{1}H NMR ($CDCl_3$, 400 MHz)

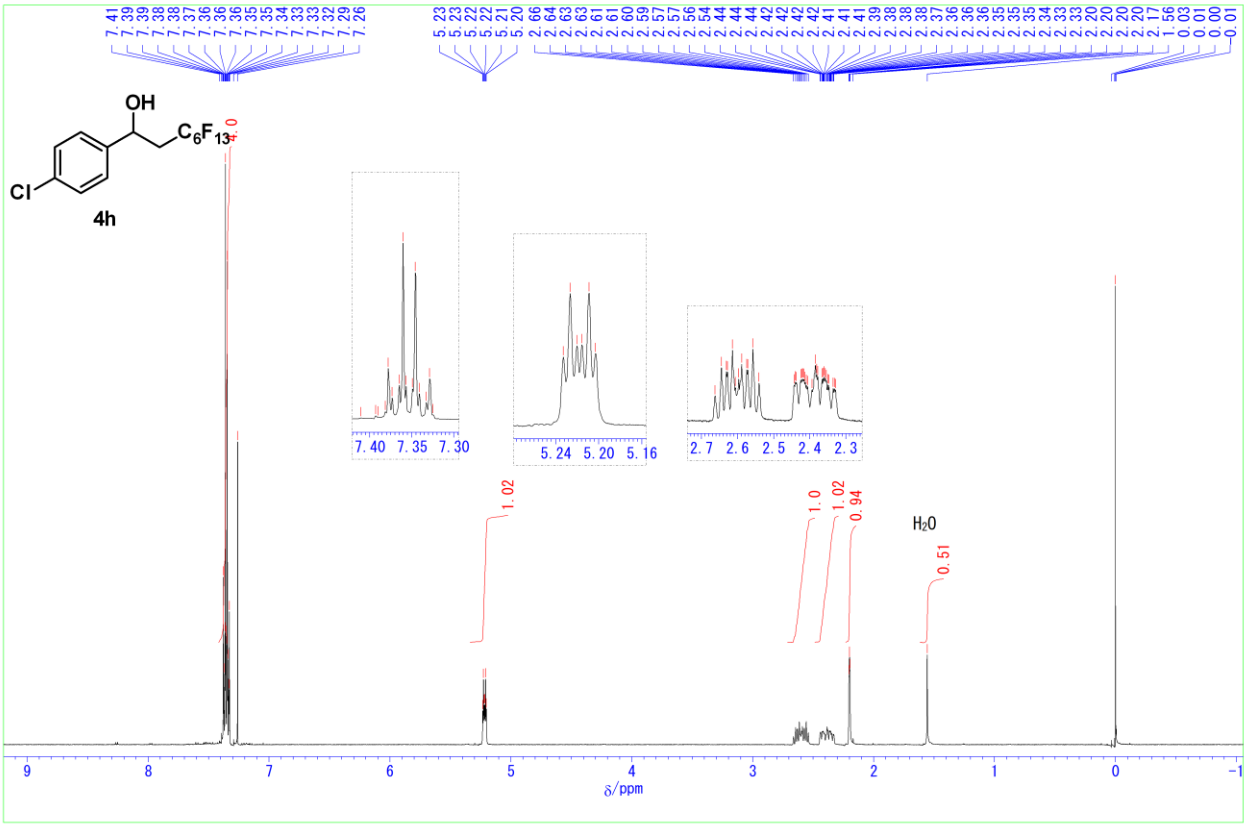

4h: ^{13}C NMR ($CDCl_3$, 151 MHz)

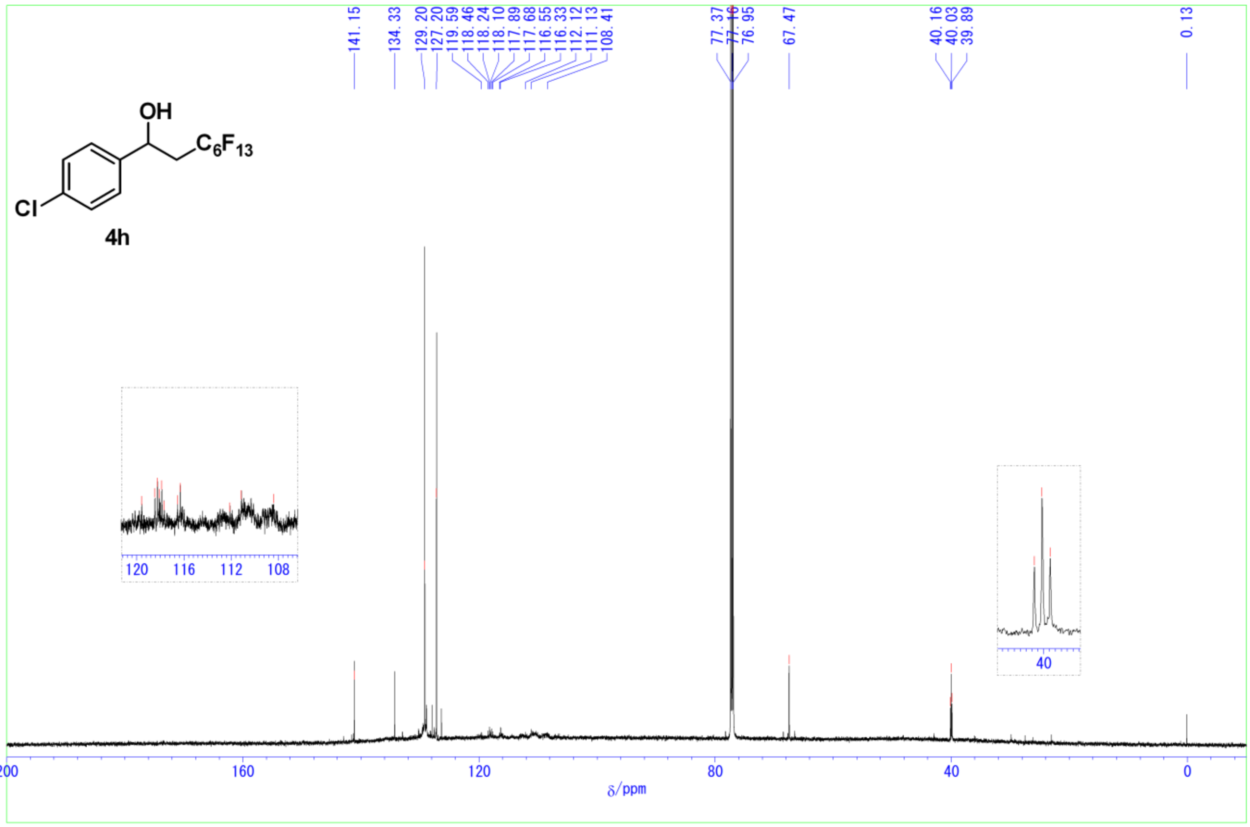

4h: ^{19}F NMR ($CDCl_3$, 376 MHz)

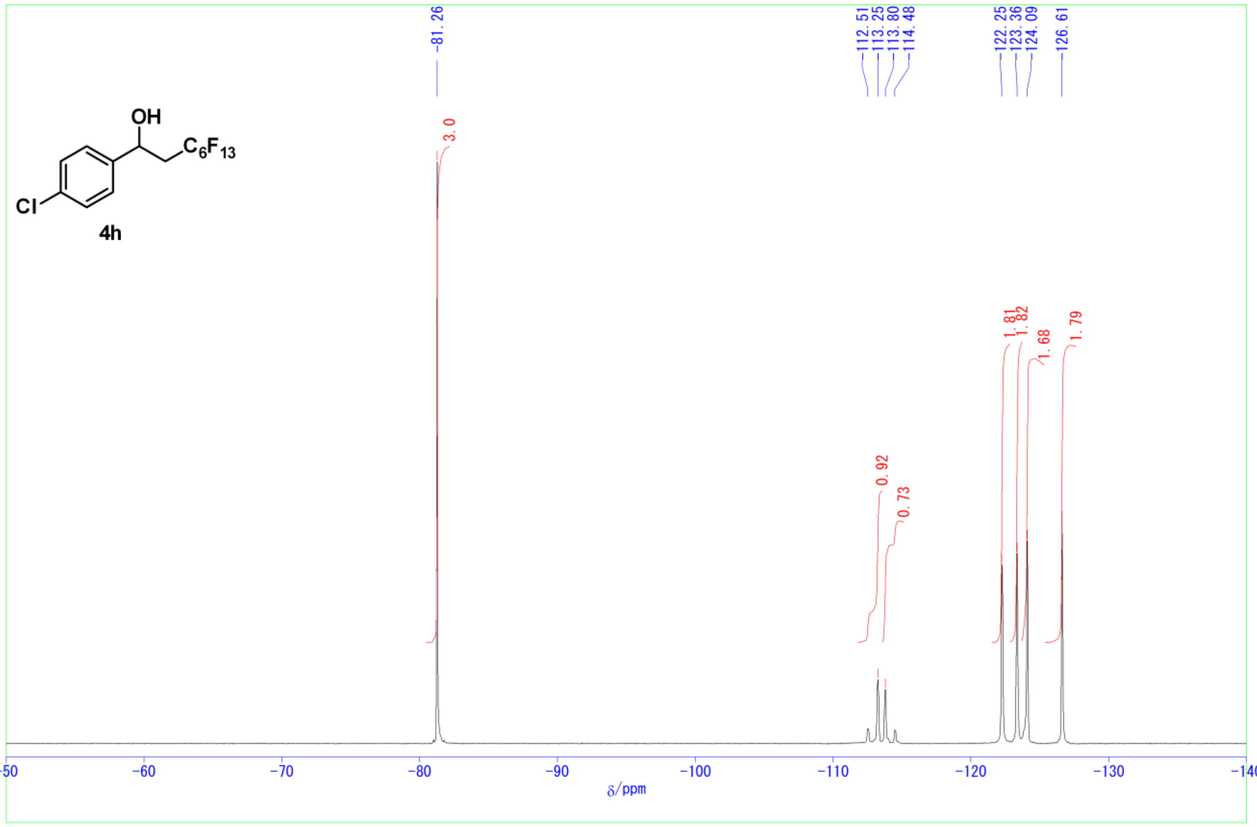

4i: ^{1}H NMR ($CDCl_3$, 400 MHz)

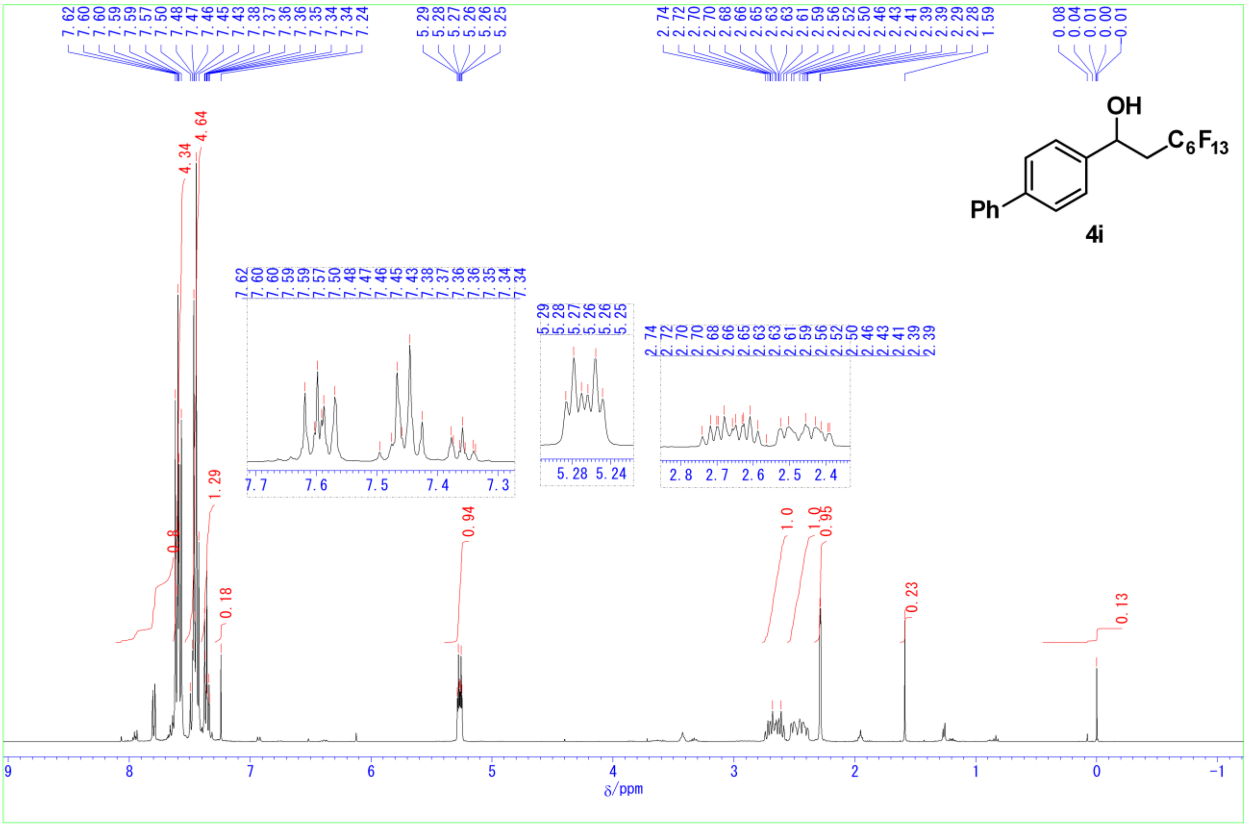

4i: ^{13}C NMR (CDCl$_3$, 151 MHz)

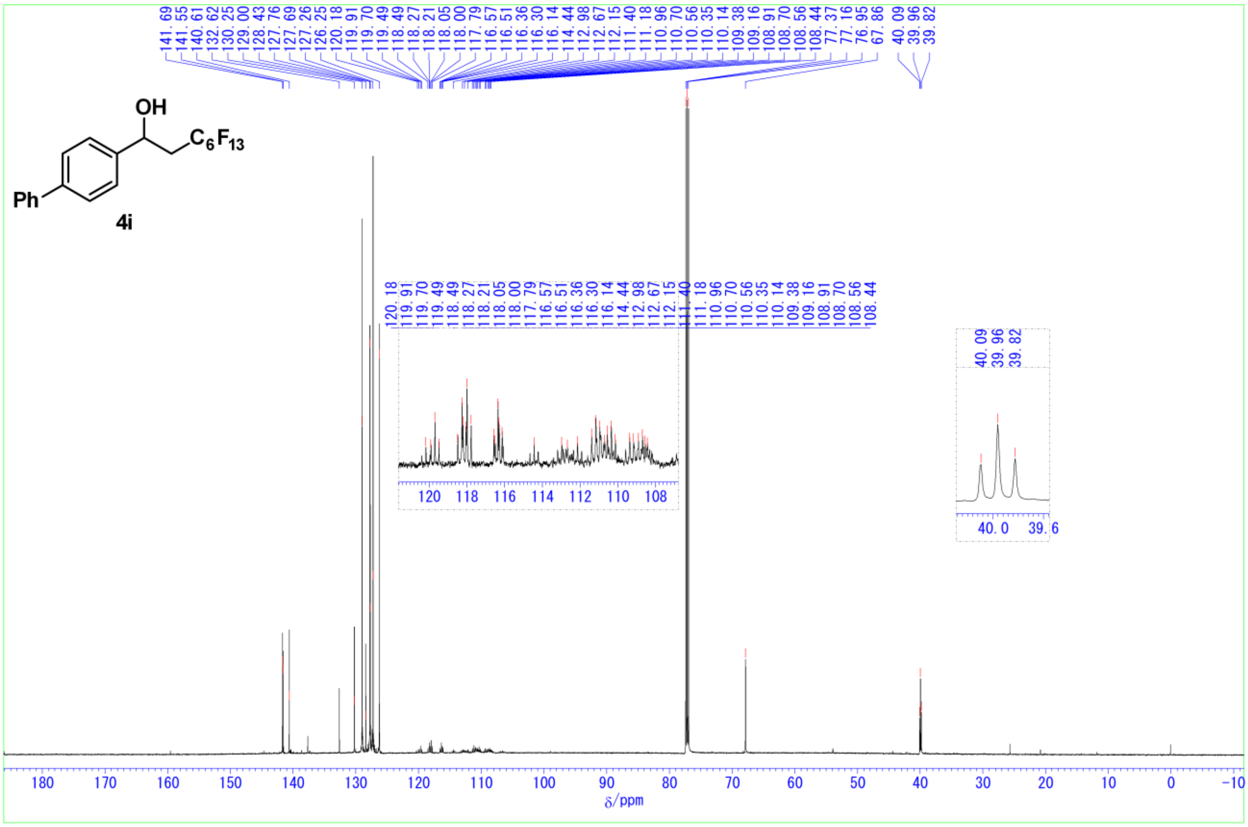

4i: ^{19}F NMR (CDCl$_3$, 376 MHz)

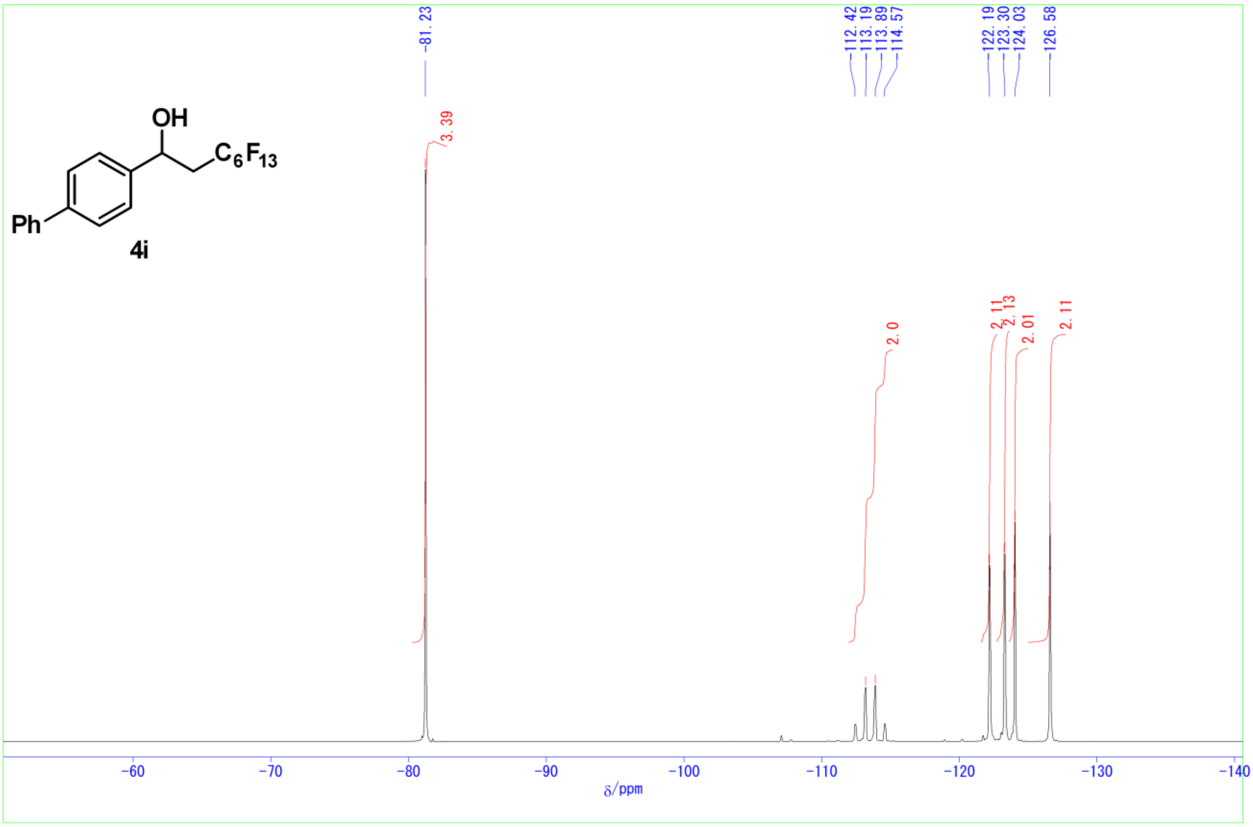

4j: ^{1}H NMR (CDCl$_3$, 400 MHz)

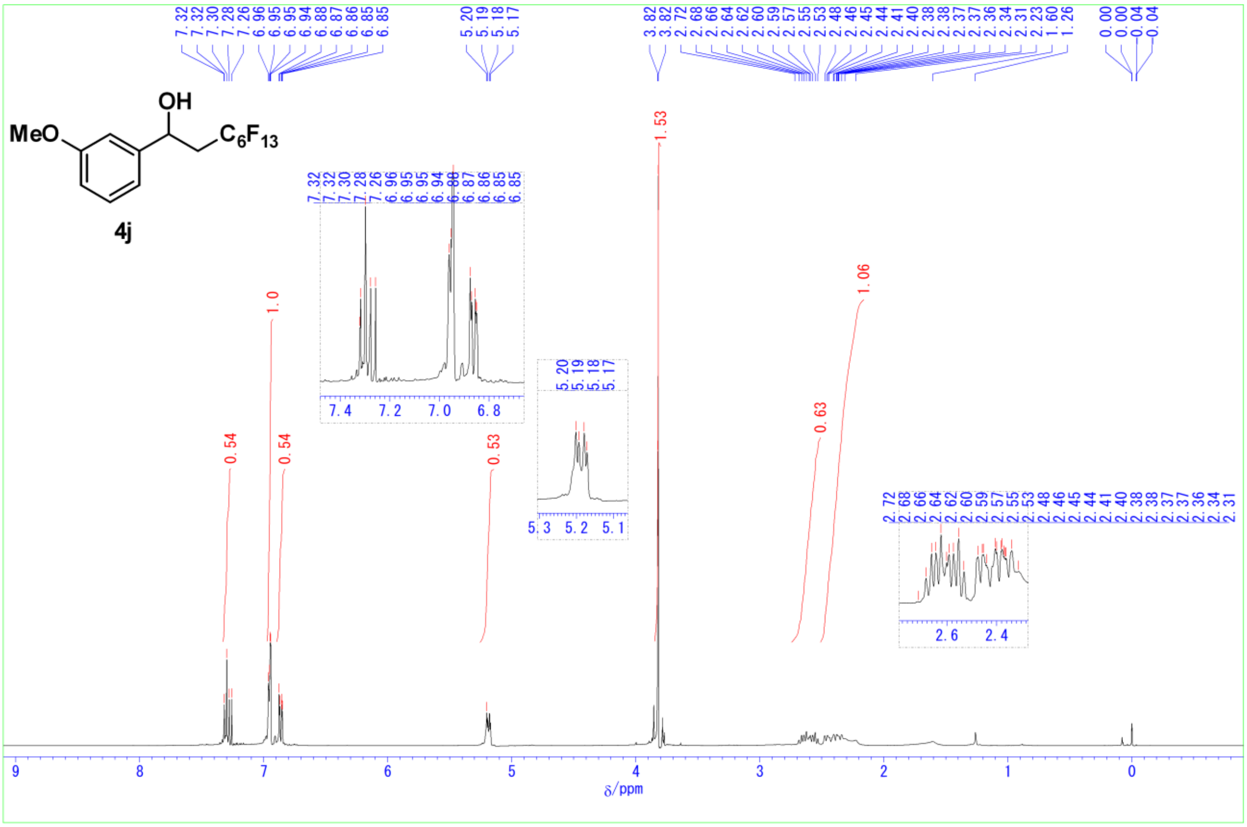

4j: ^{13}C NMR (CDCl$_3$, 151 MHz)

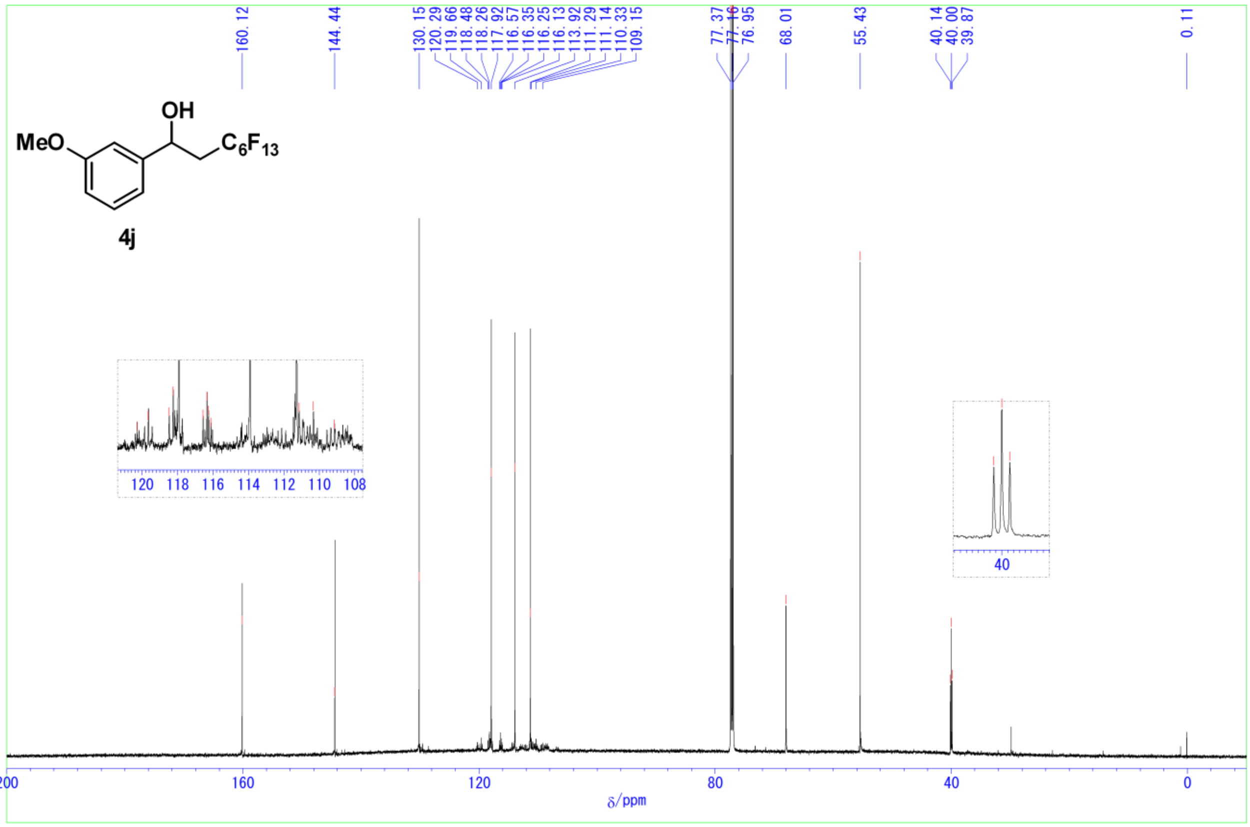

4j: ^{19}F NMR ($CDCl_3$, 376 MHz)

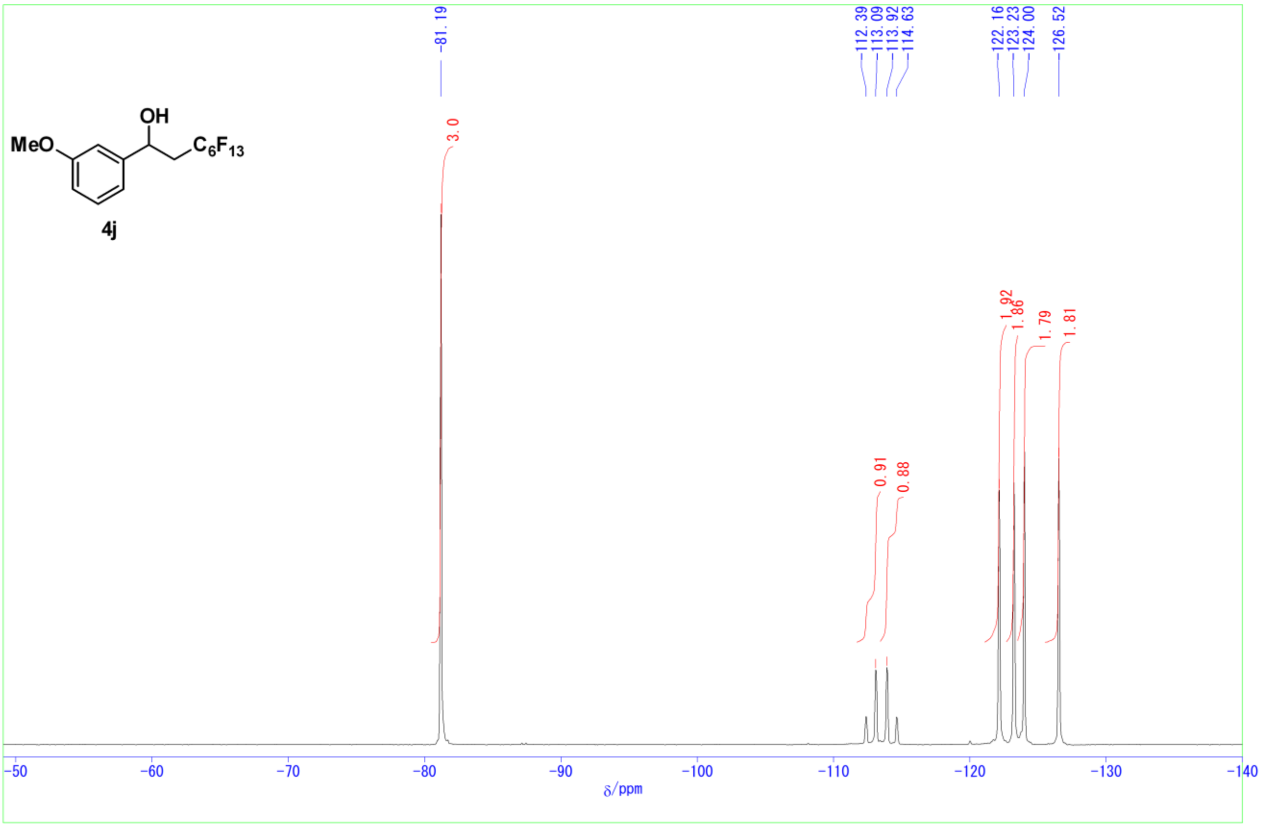

4k: ^{1}H NMR ($CDCl_3$, 400 MHz)

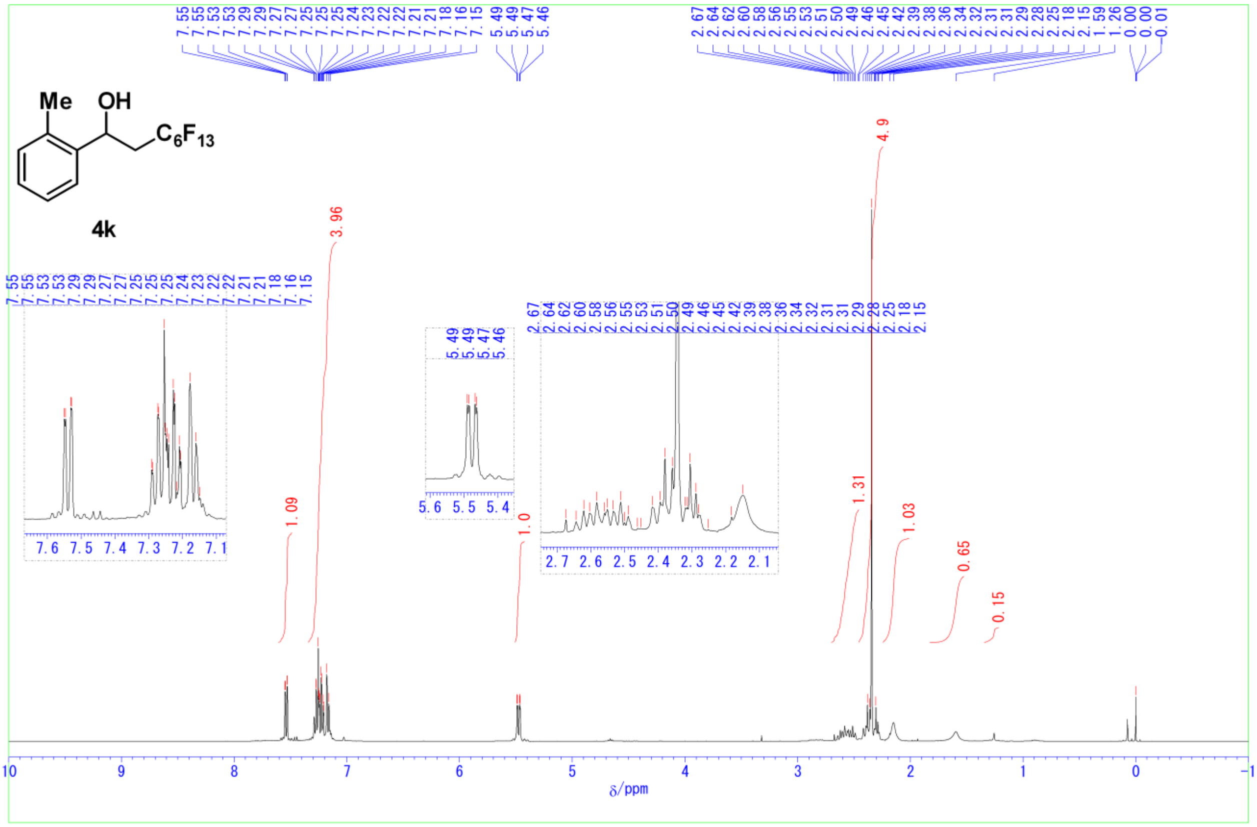

4k: ^{13}C NMR ($CDCl_3$, 151 MHz)

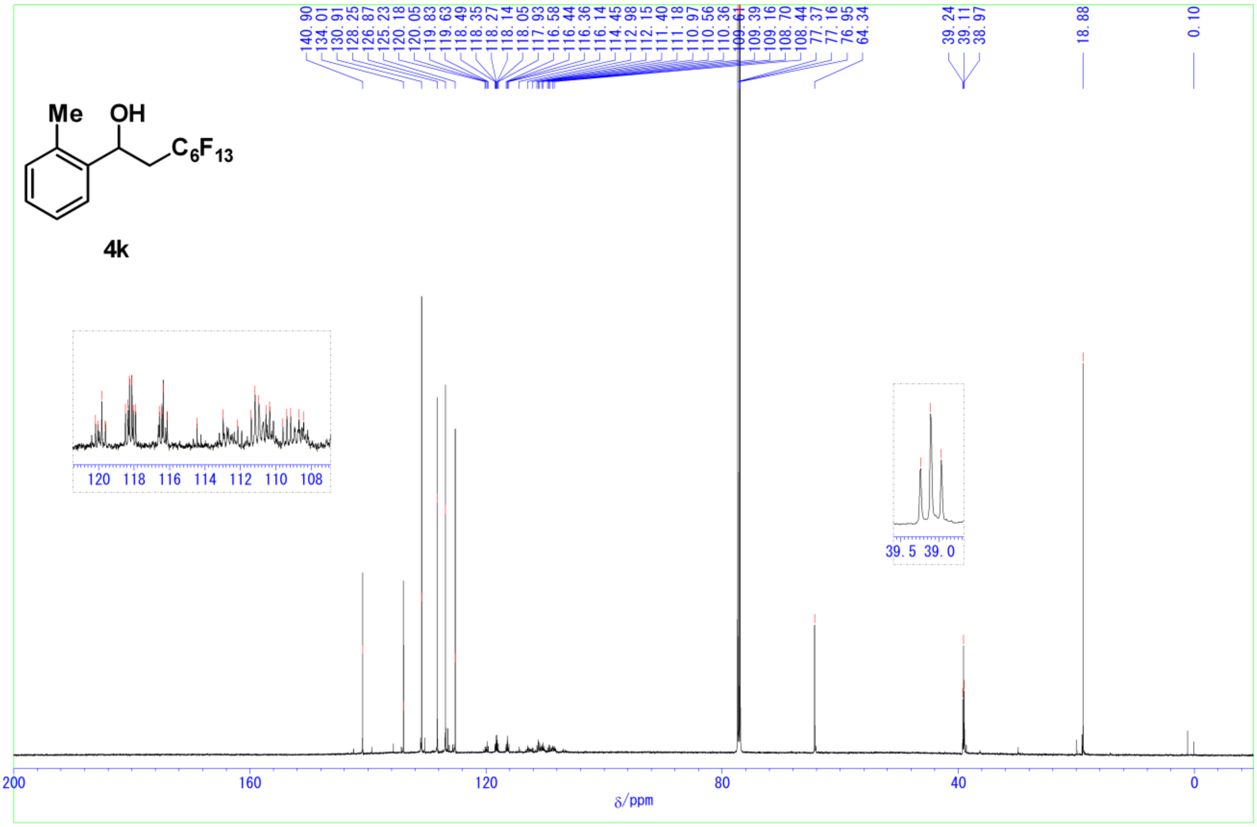

4k: ^{19}F NMR ($CDCl_3$, 376 MHz)

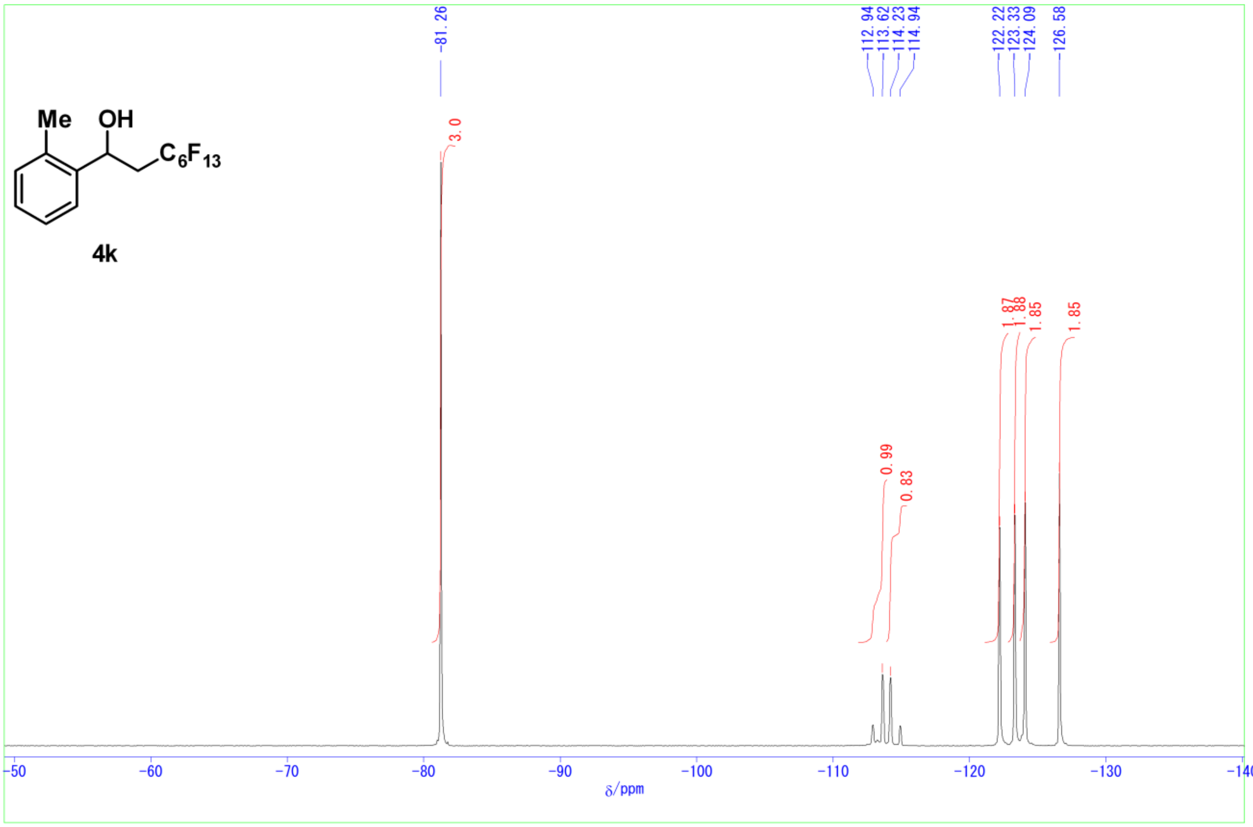

4l: ^{1}H NMR ($CDCl_3$, 400 MHz)

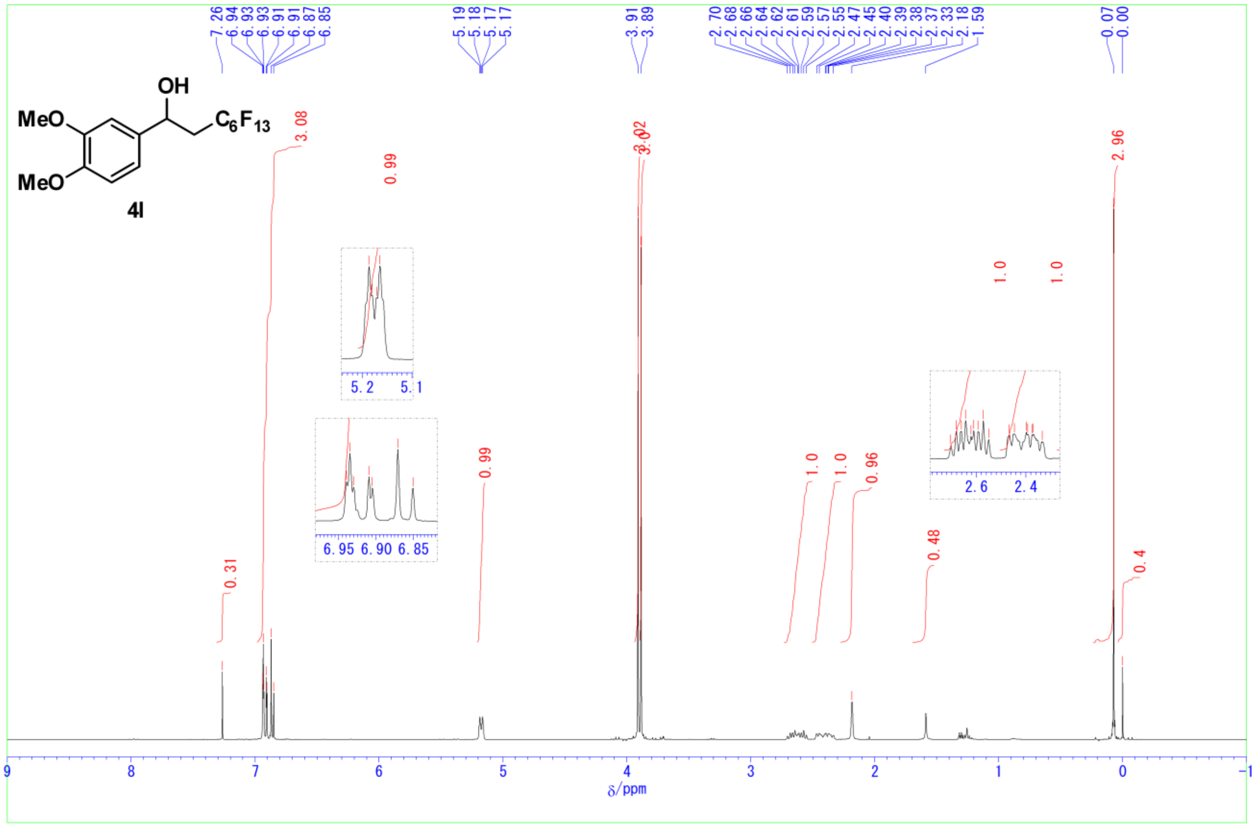

4l: ^{13}C NMR ($CDCl_3$, 151 MHz)

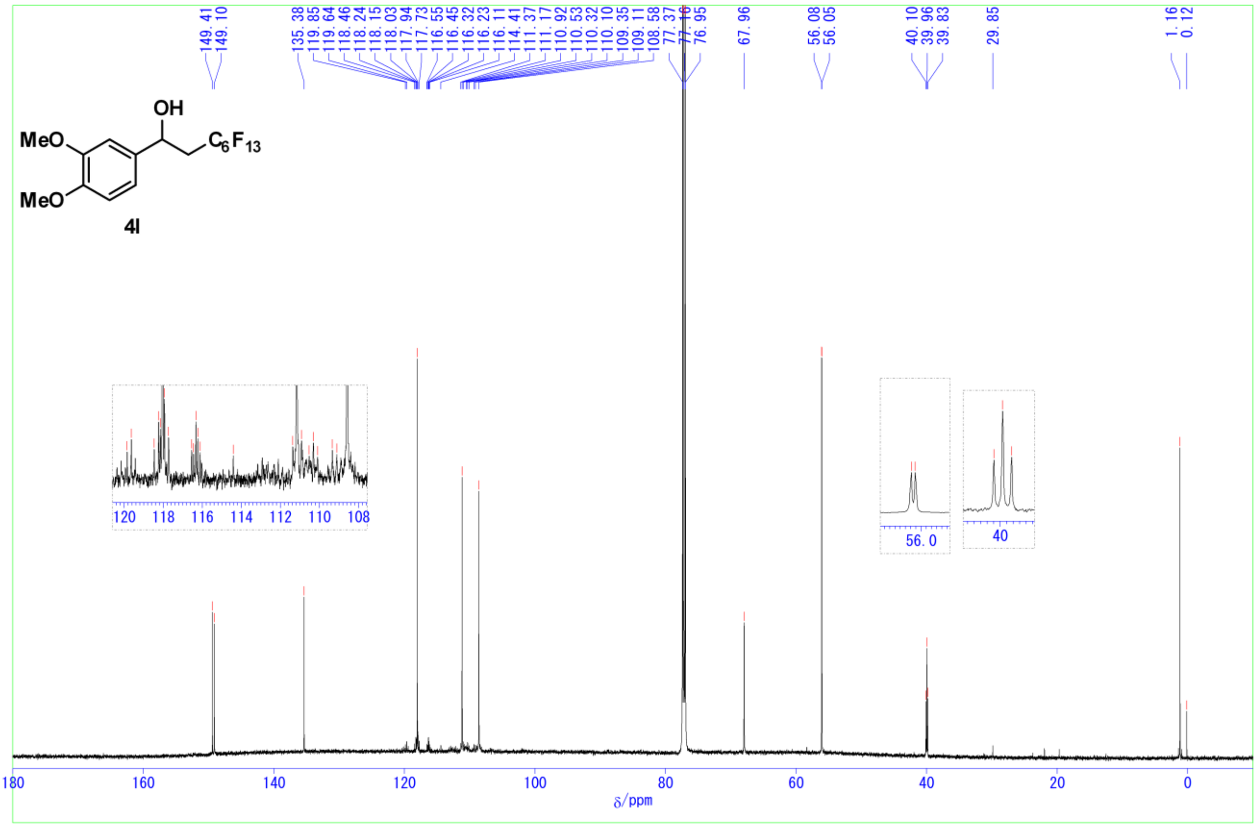

4l: ^{19}F NMR ($CDCl_3$, 376 MHz)

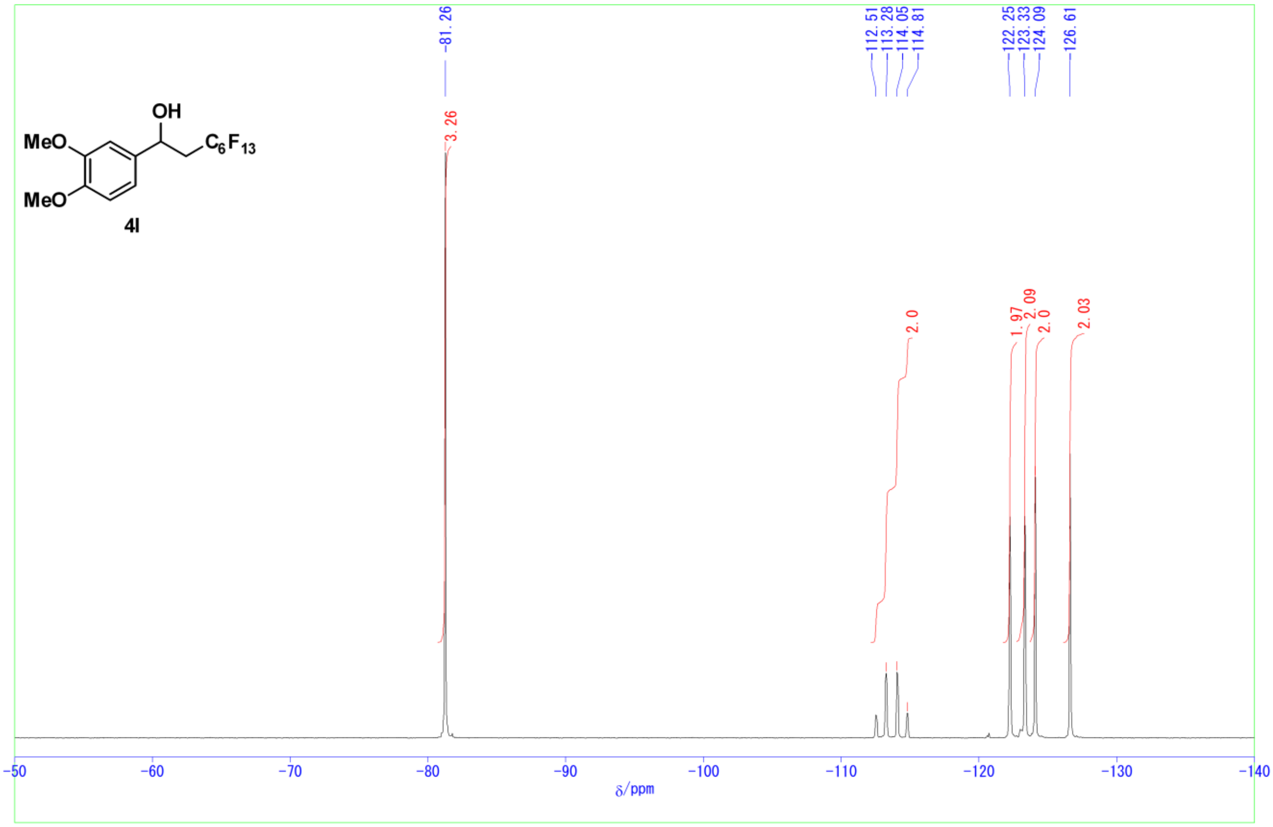

4m: ^{1}H NMR ($CDCl_3$, 400 MHz)

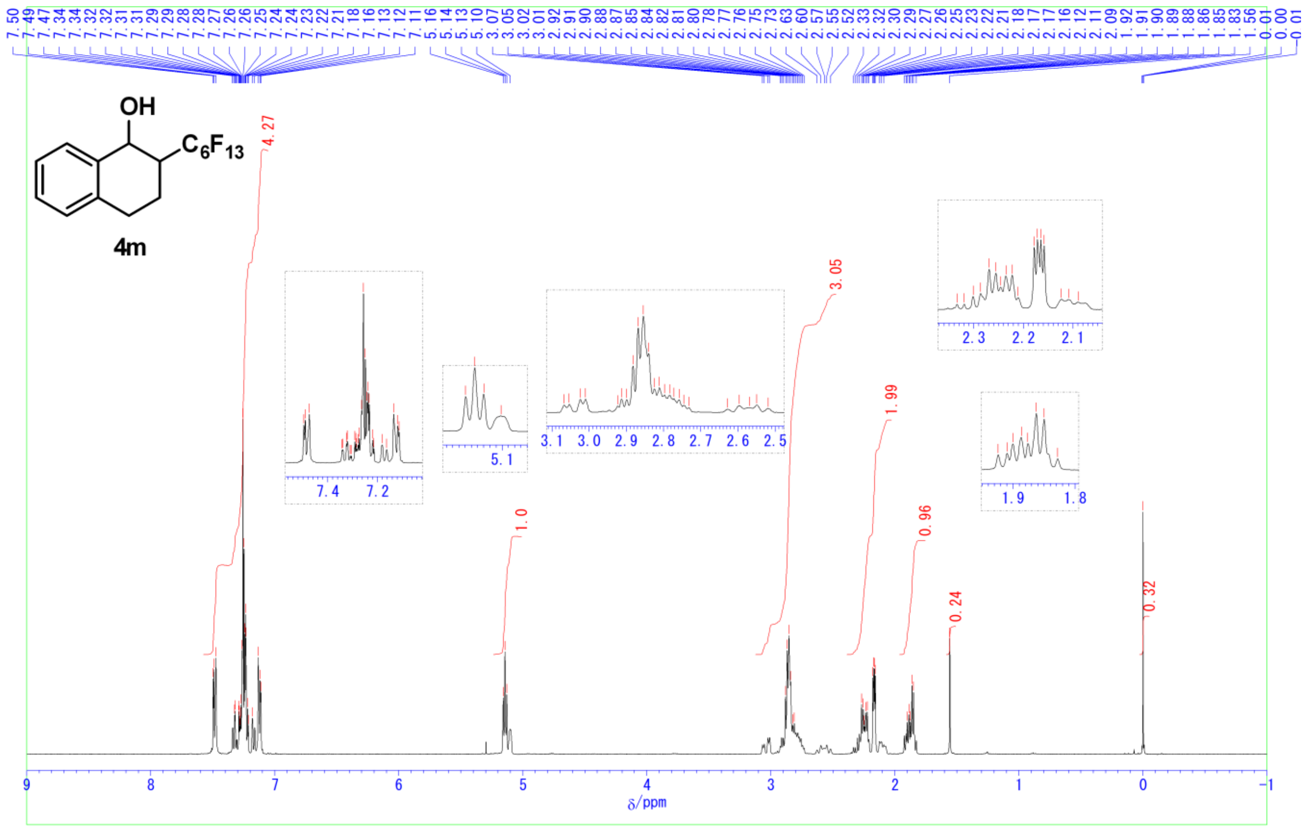

4m: ^{13}C NMR ($CDCl_3$, 151 MHz)

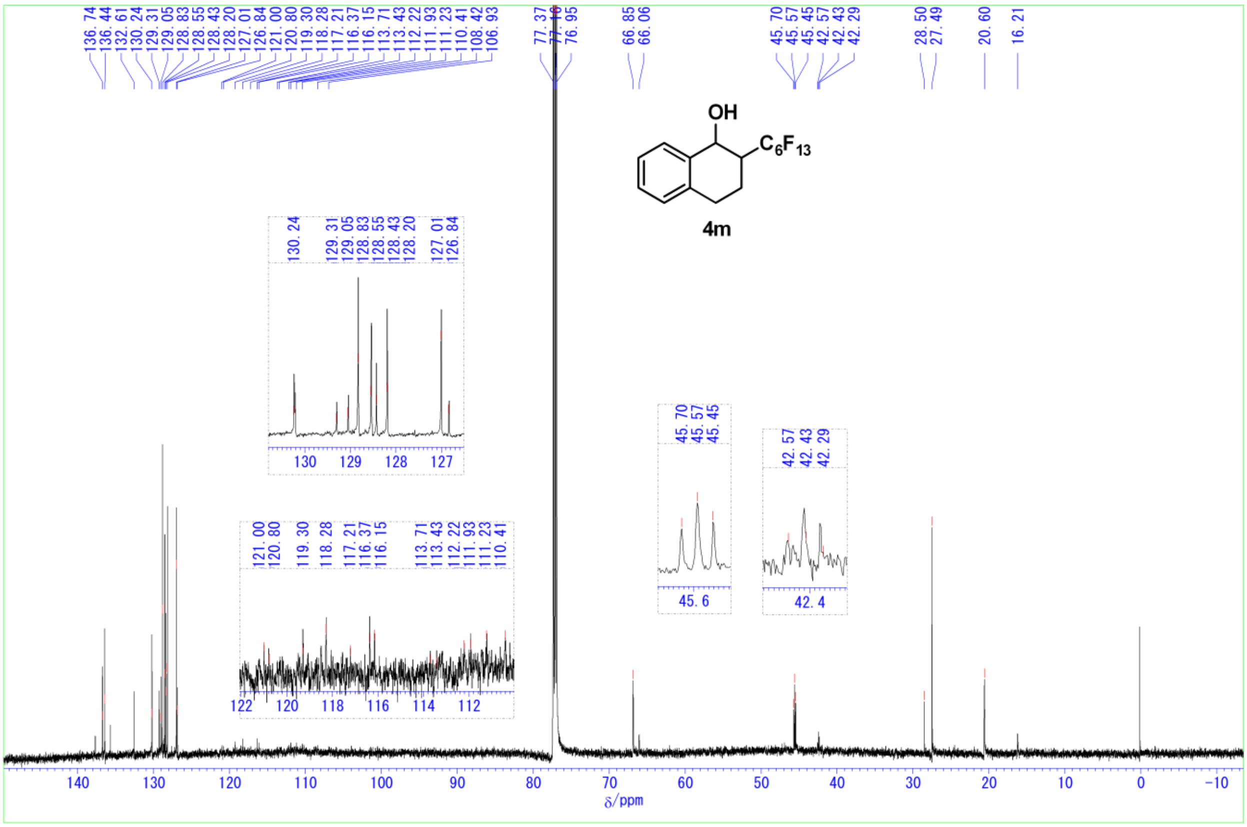

4m: ^{19}F NMR ($CDCl_3$, 376 MHz)

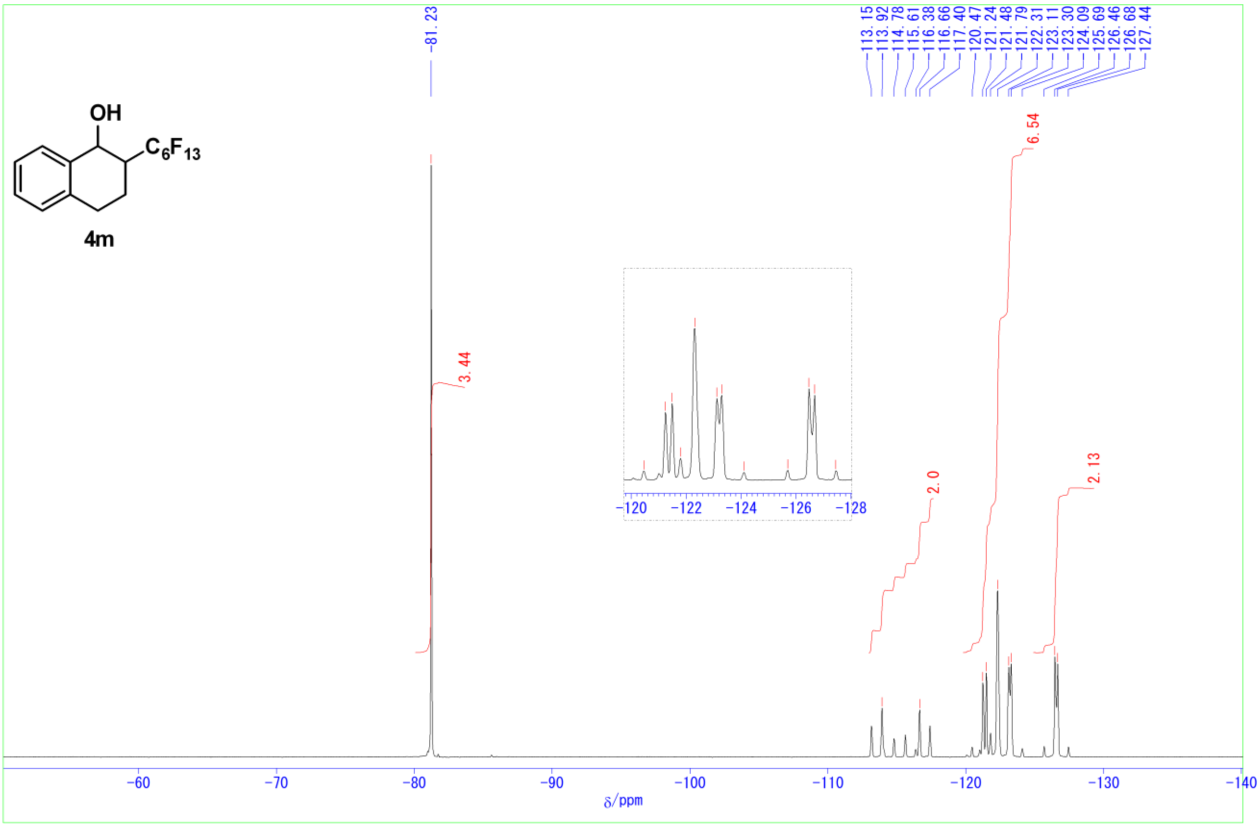

4n: ^{1}H NMR ($CDCl_3$, 400 MHz)

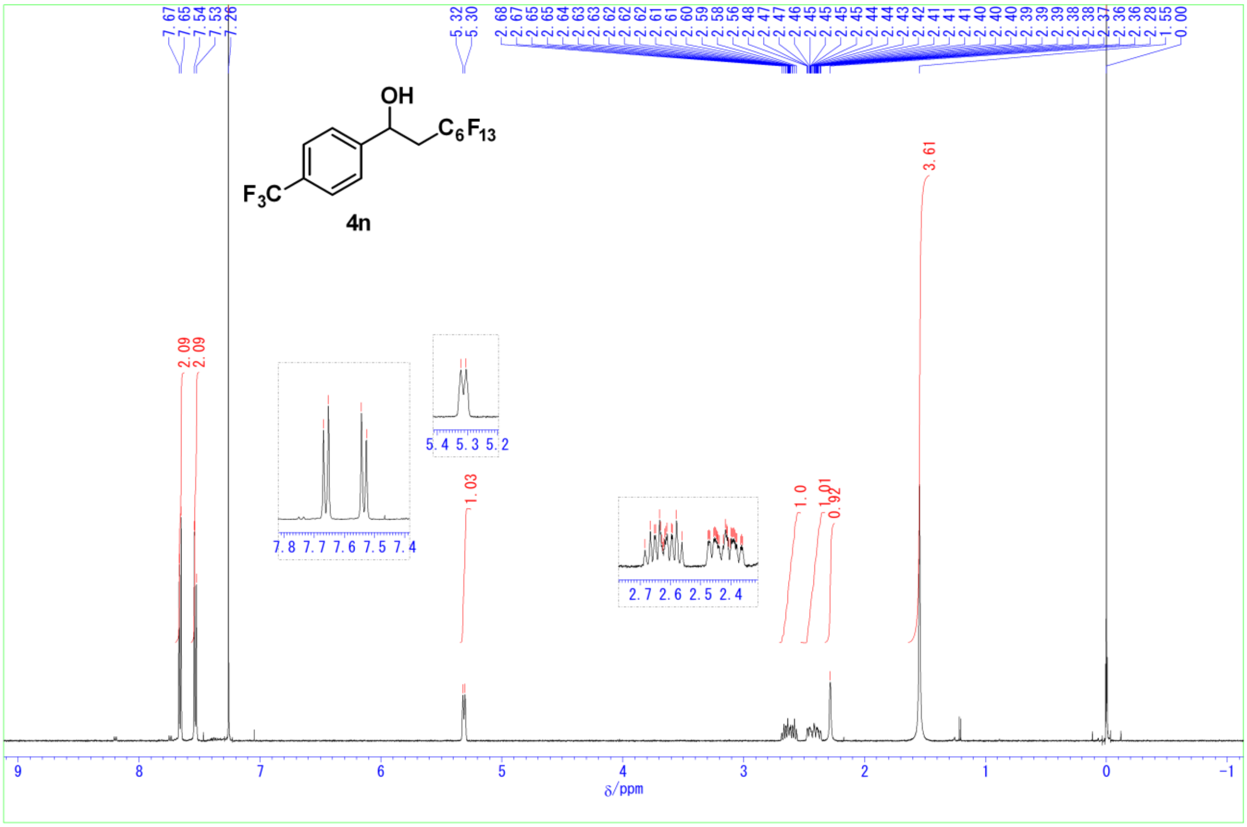

4n: ^{13}C NMR ($CDCl_3$, 151 MHz)

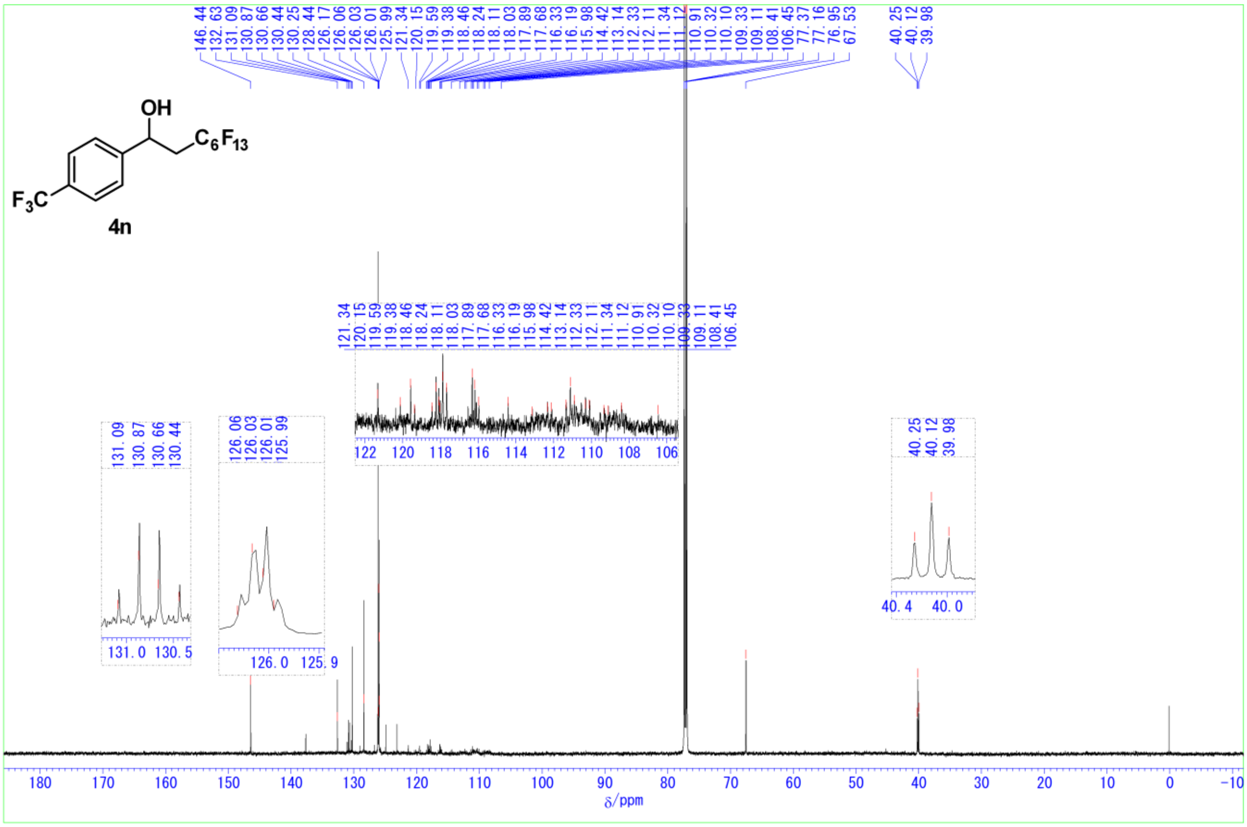

4n: ^{19}F NMR ($CDCl_3$, 376 MHz)

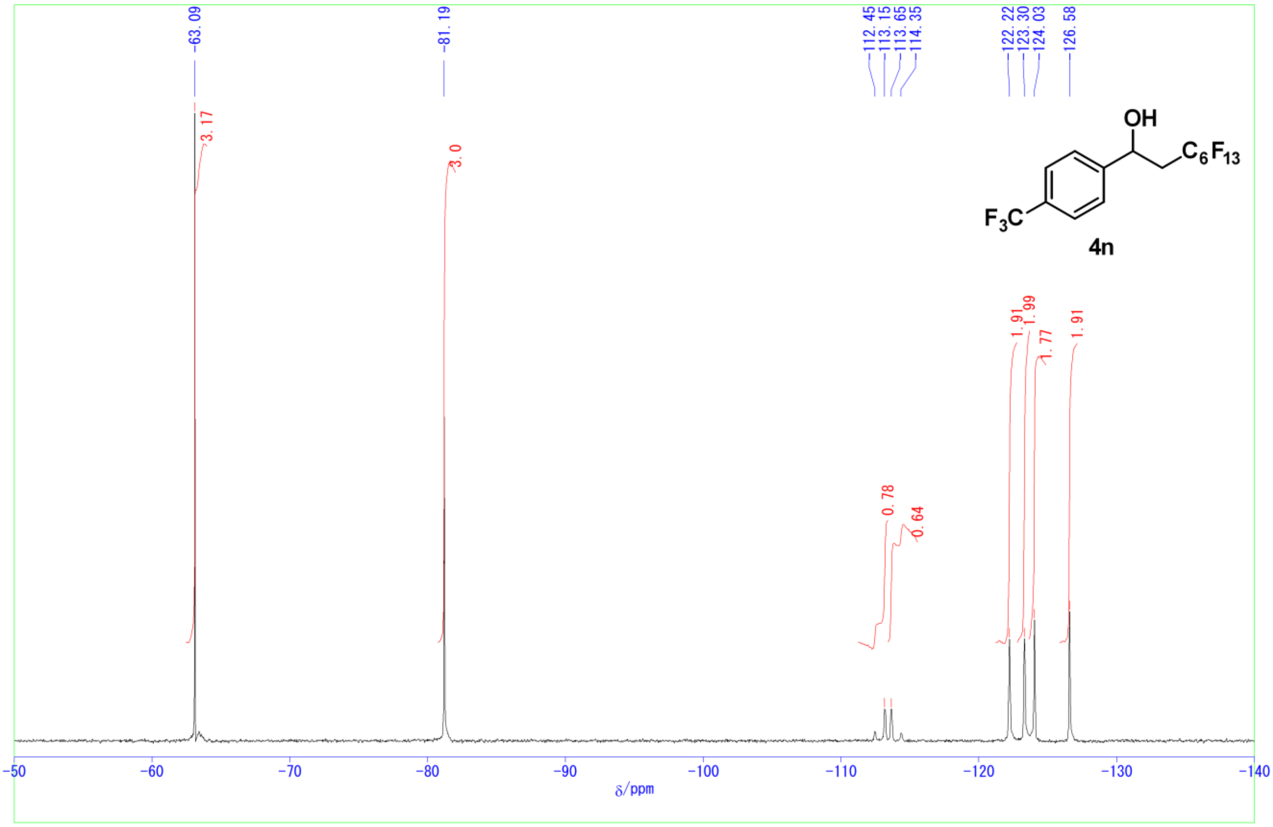

4o: ^{1}H NMR ($CDCl_3$, 500 MHz)

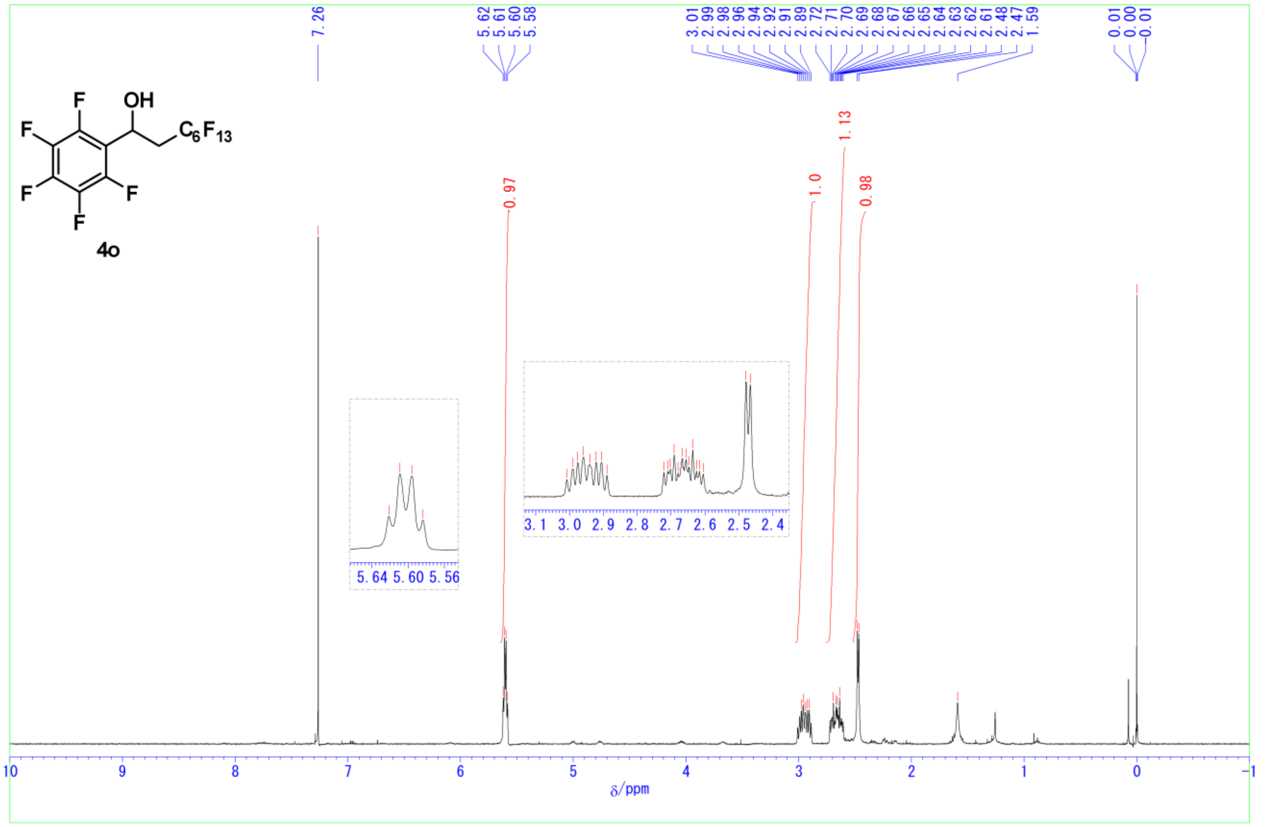

4o: ^{13}C NMR ($CDCl_3$, 151 MHz)

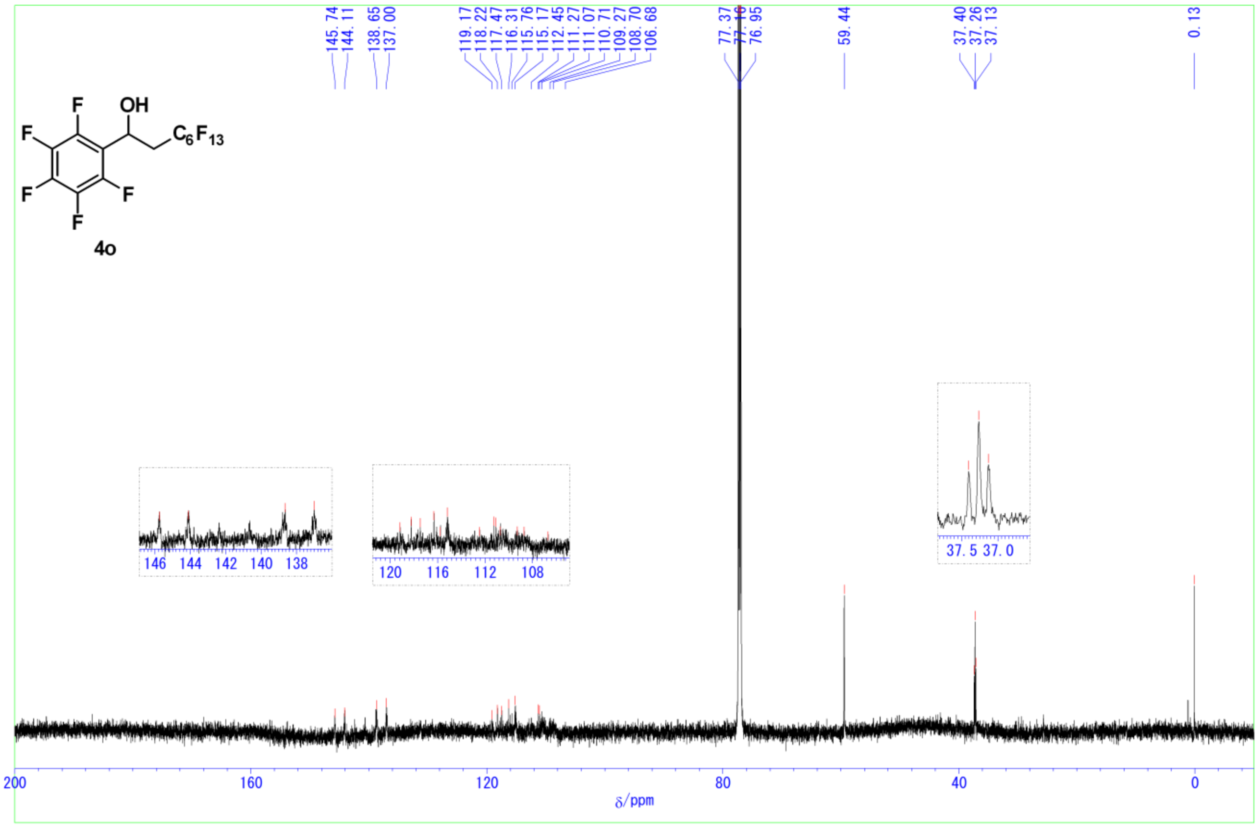

4o:^{19}F NMR ($CDCl_3$, 471 MHz)

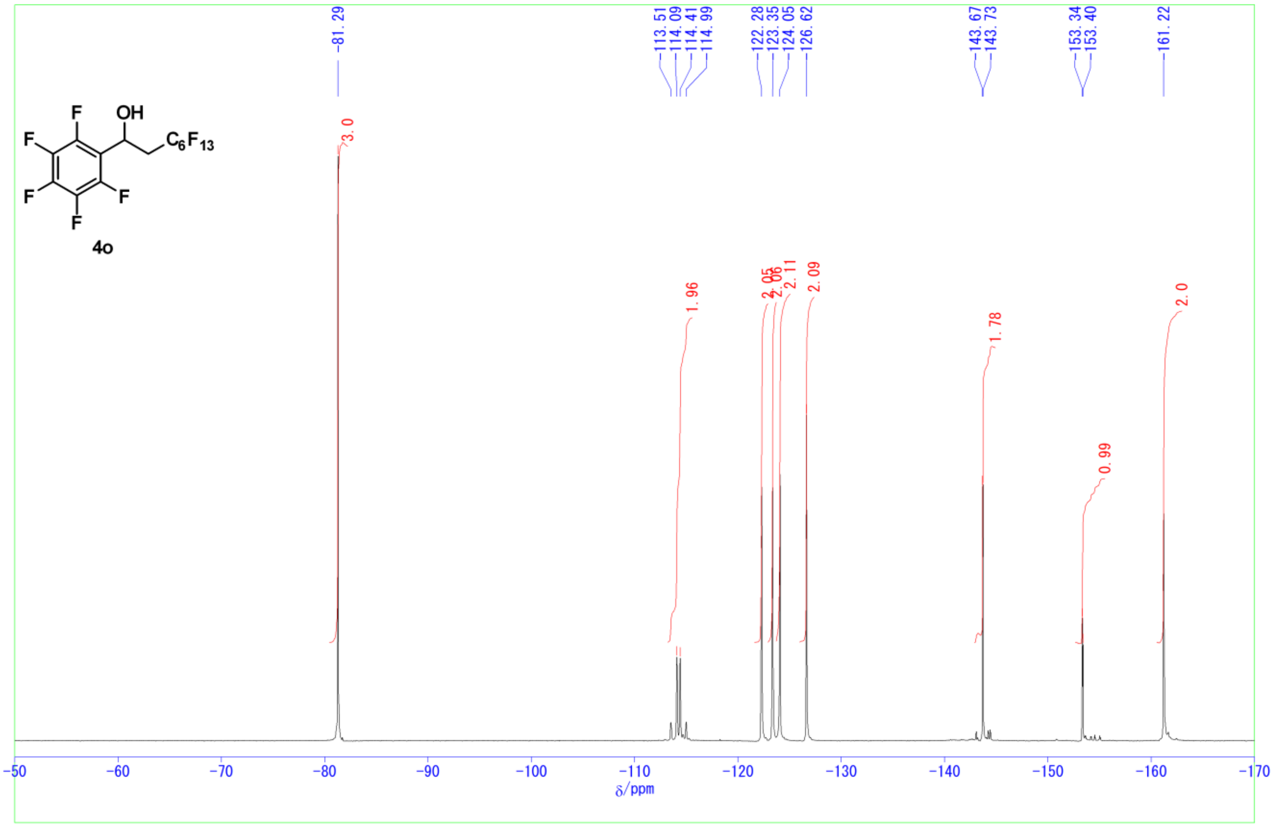

4p: ^{1}H NMR ($CDCl_3$, 400 MHz)

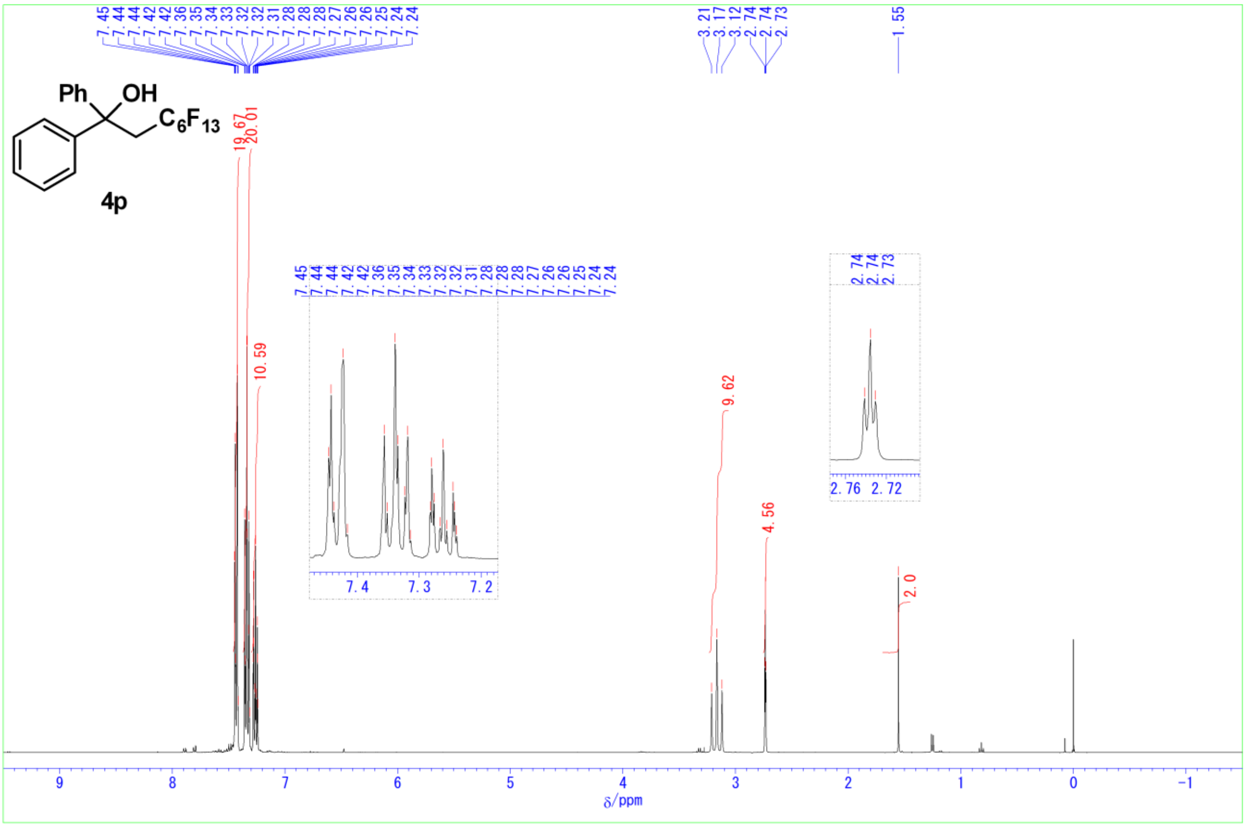

4p: ^{13}C NMR ($CDCl_3$, 151 MHz)

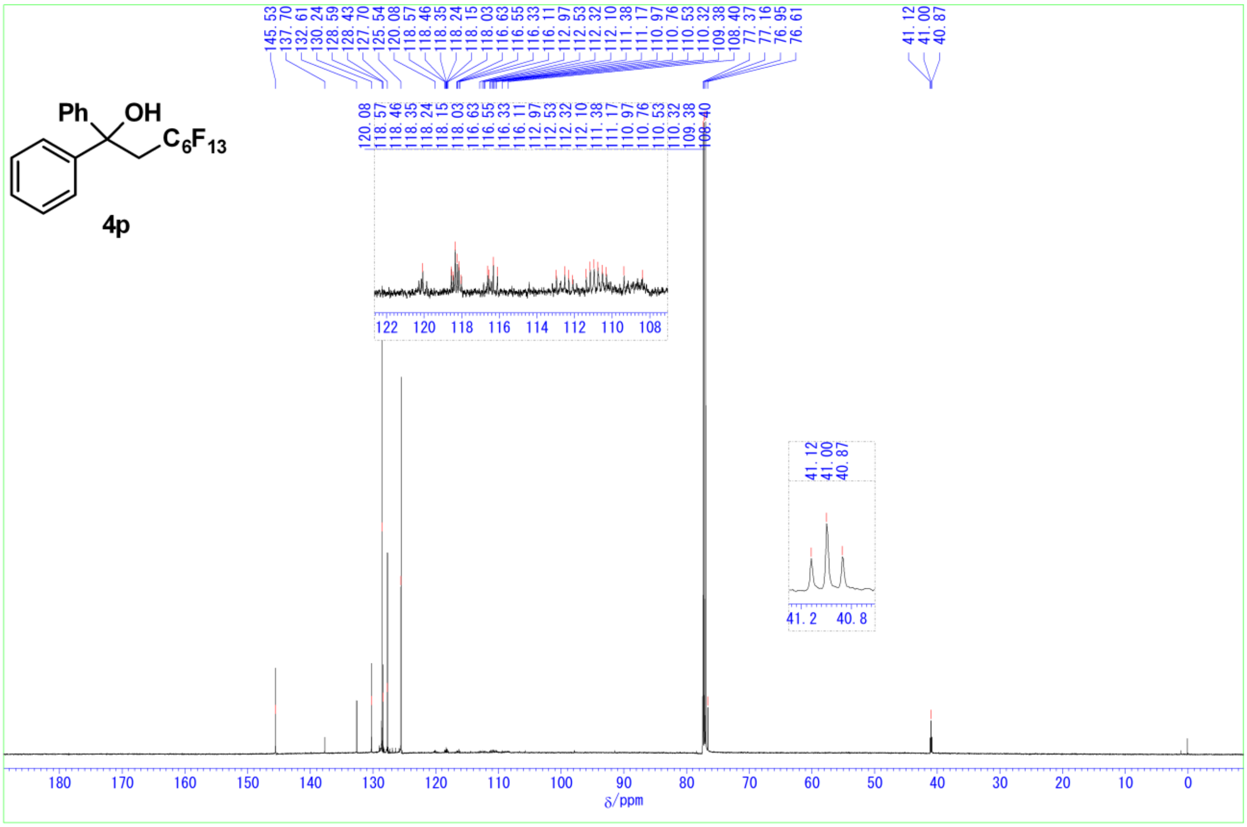

4p: ^{19}F NMR ($CDCl_3$, 376 MHz)

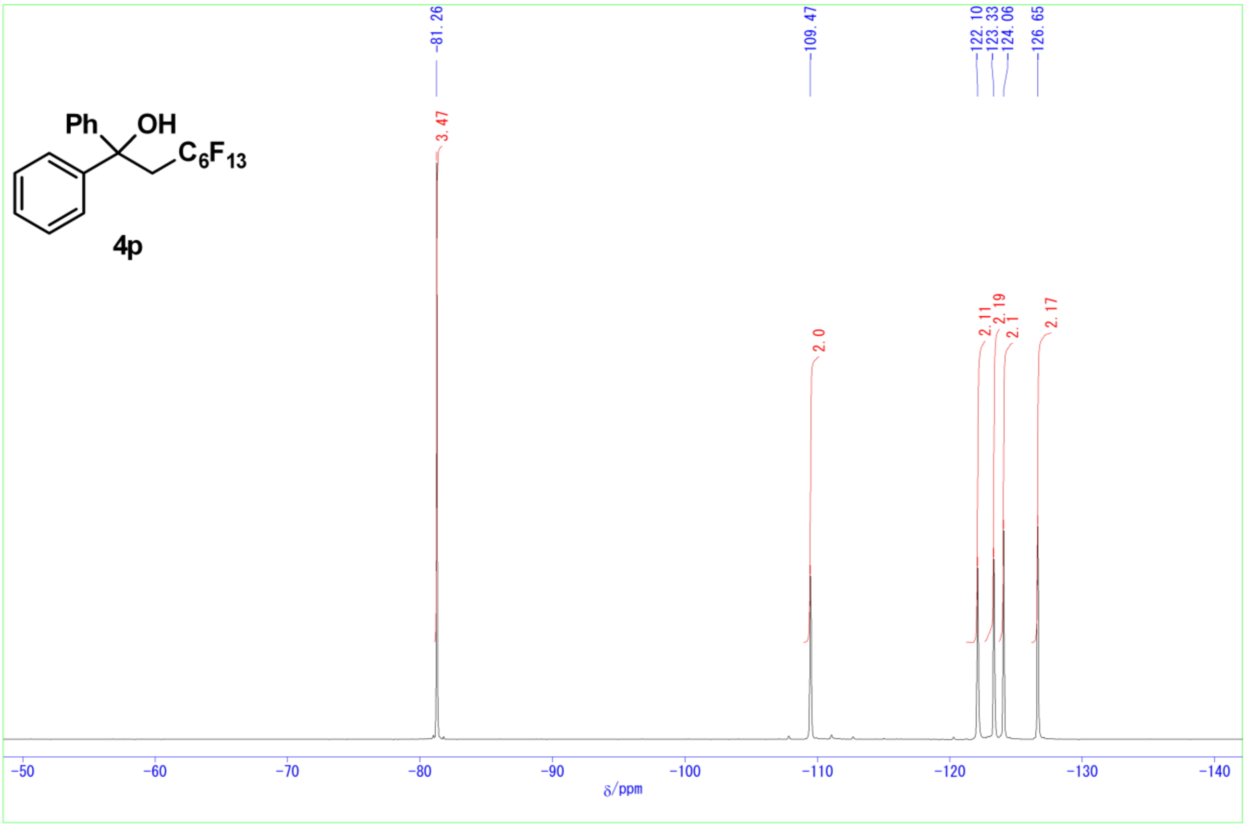

5b: ^{1}H NMR ($CDCl_3$, 400 MHz)

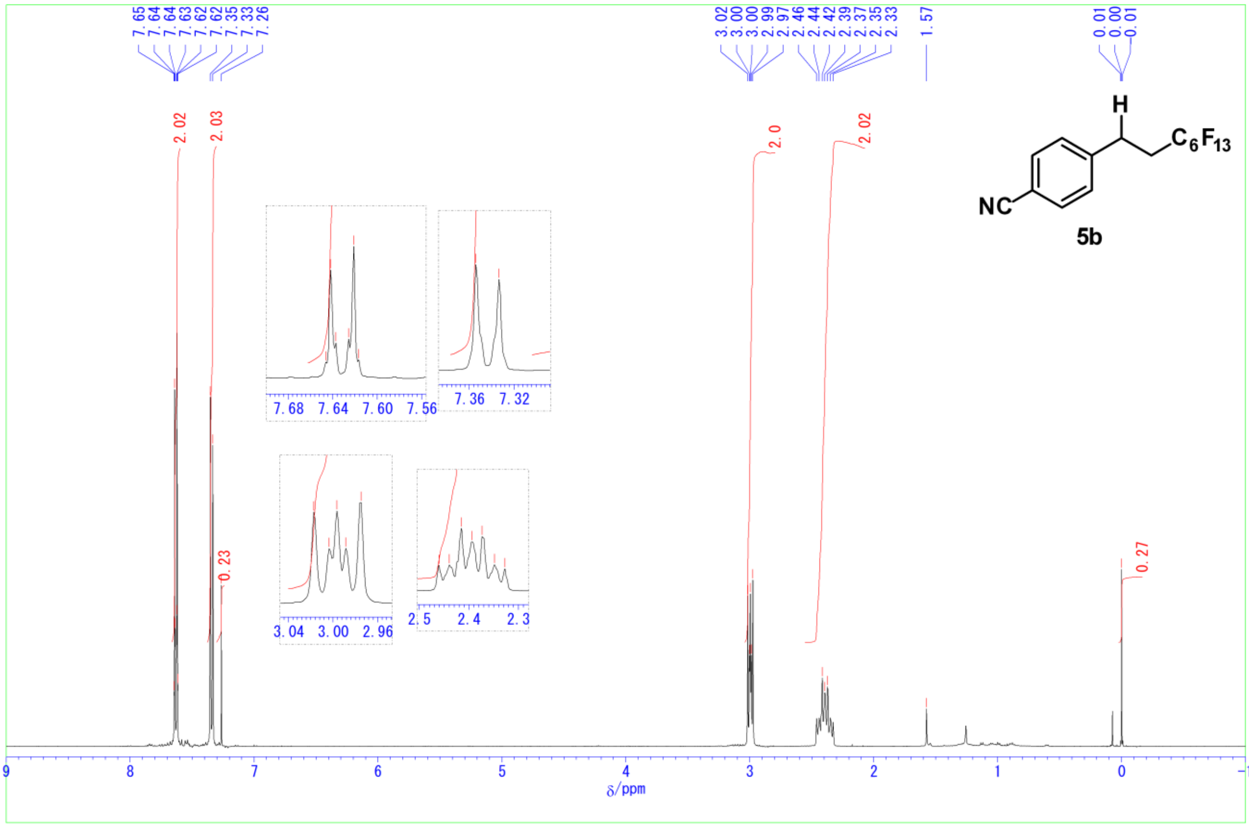

5b: ^{13}C NMR ($CDCl_3$, 151 MHz)

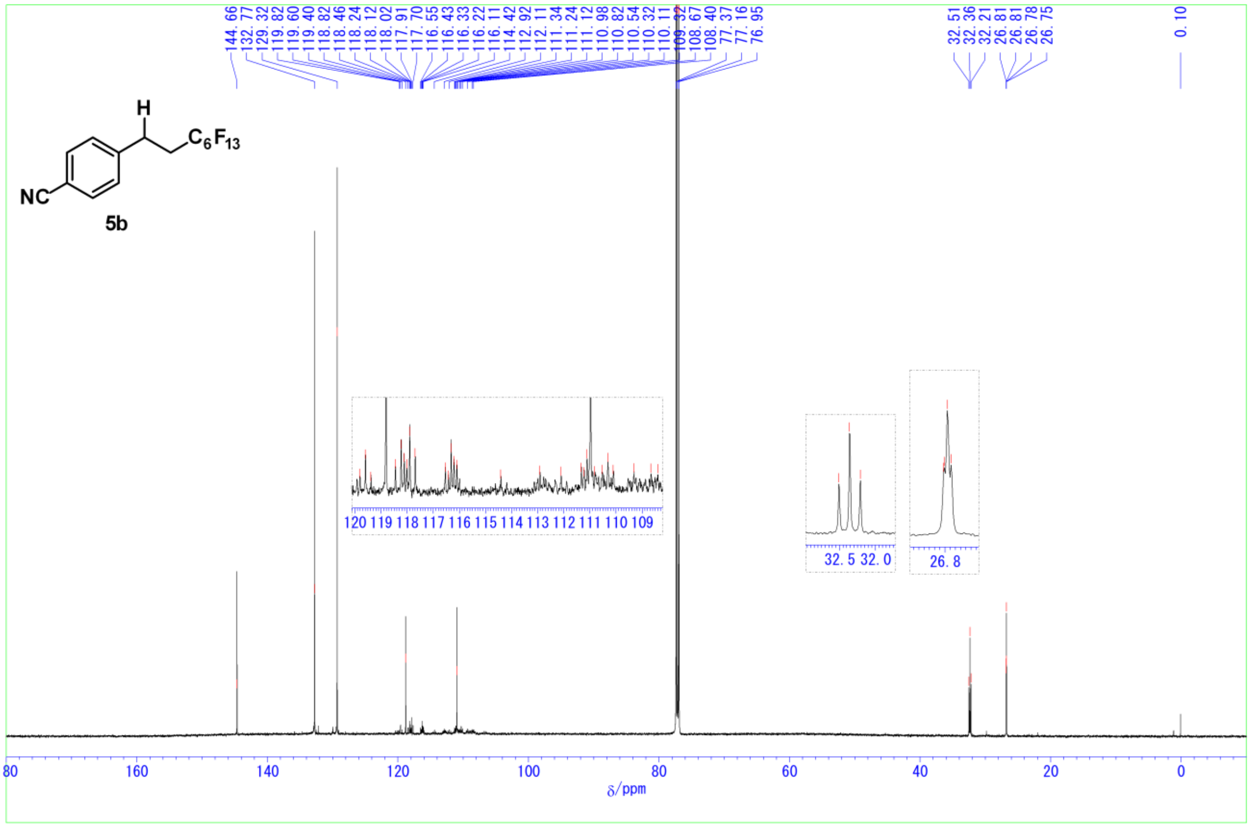

5b: ^{19}F NMR ($CDCl_3$, 376 MHz)

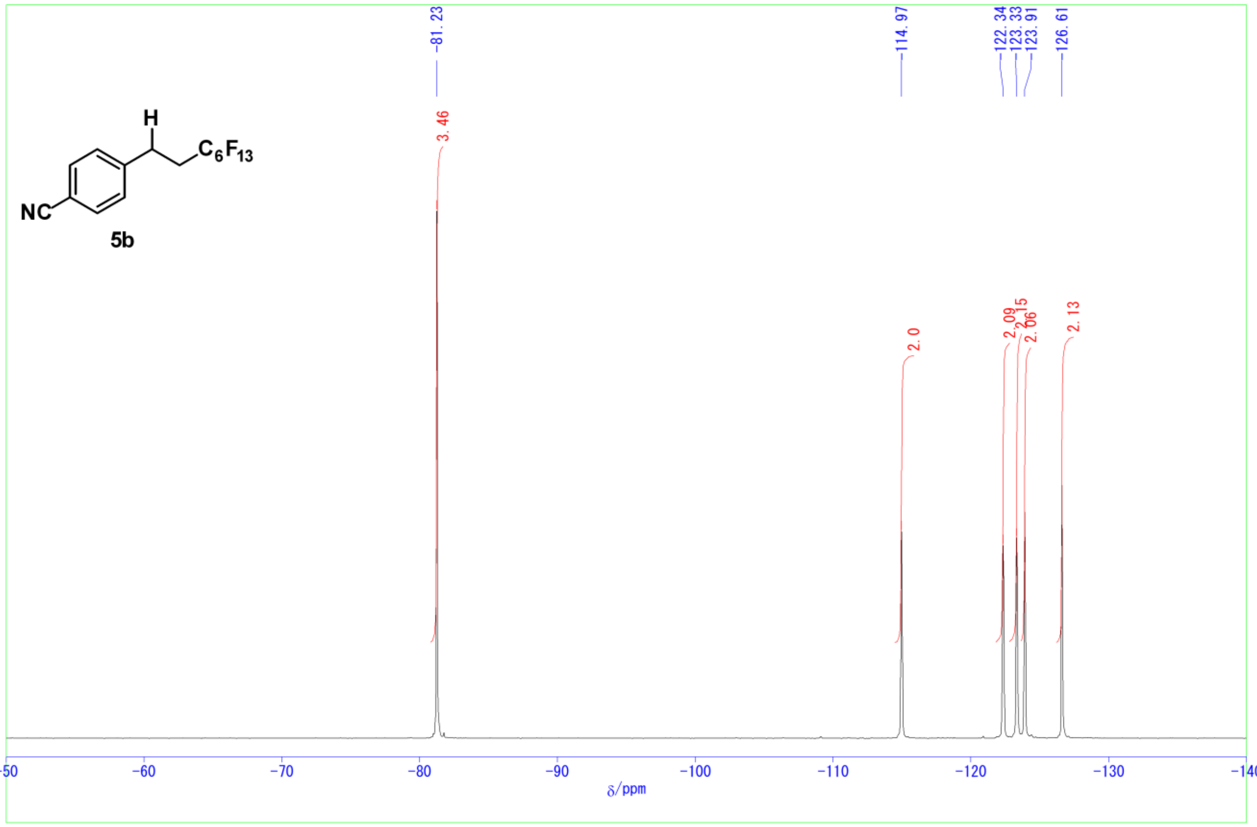

5bc: ^{1}H NMR ($CDCl_3$, 400 MHz)

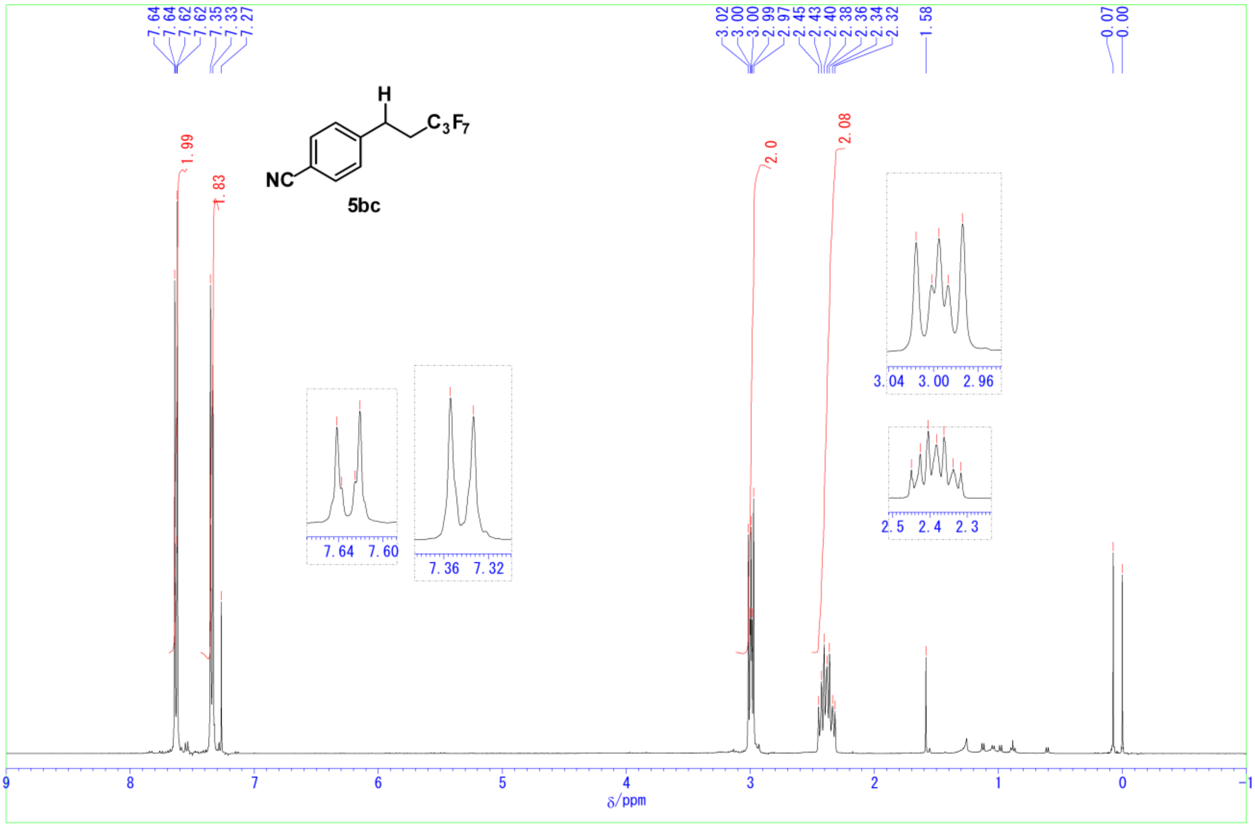

5bc: ^{13}C NMR ($CDCl_3$, 151 MHz)

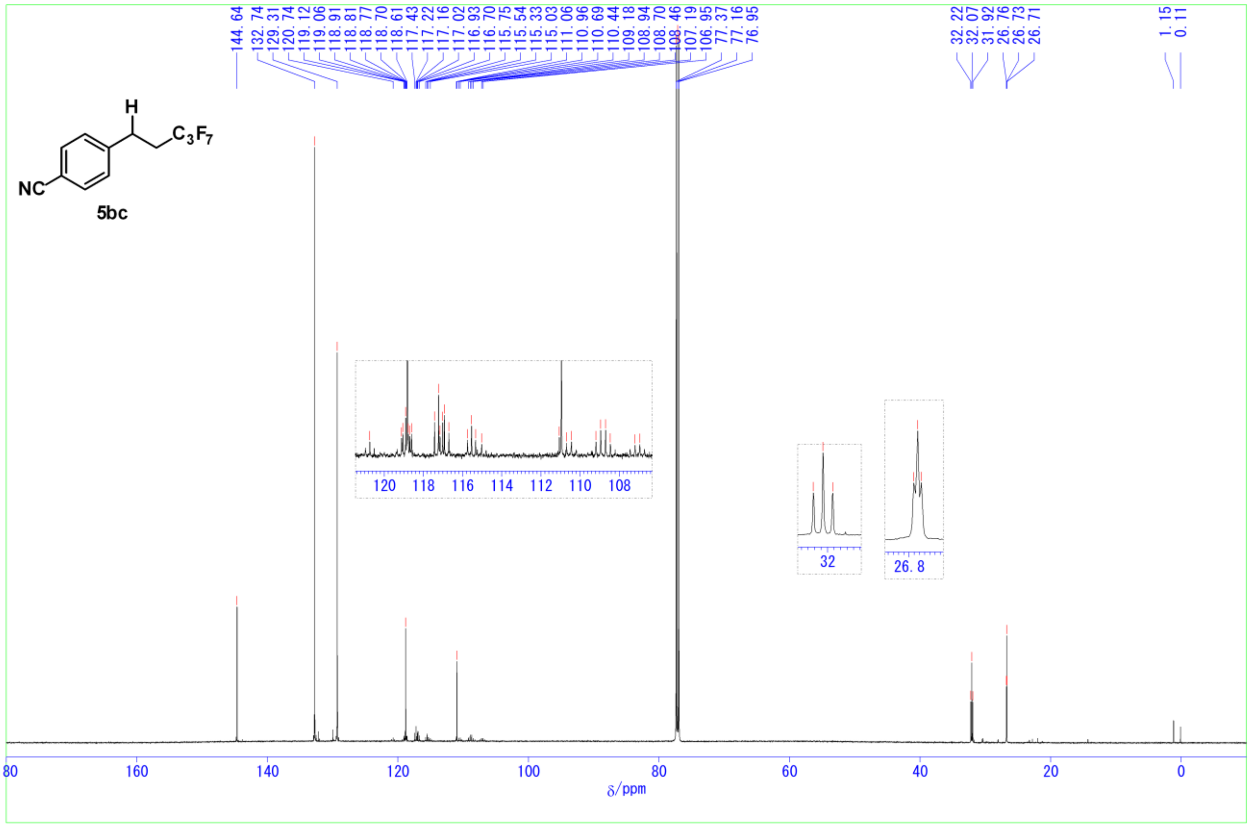

5bc: ^{19}F NMR ($CDCl_3$, 376 MHz)

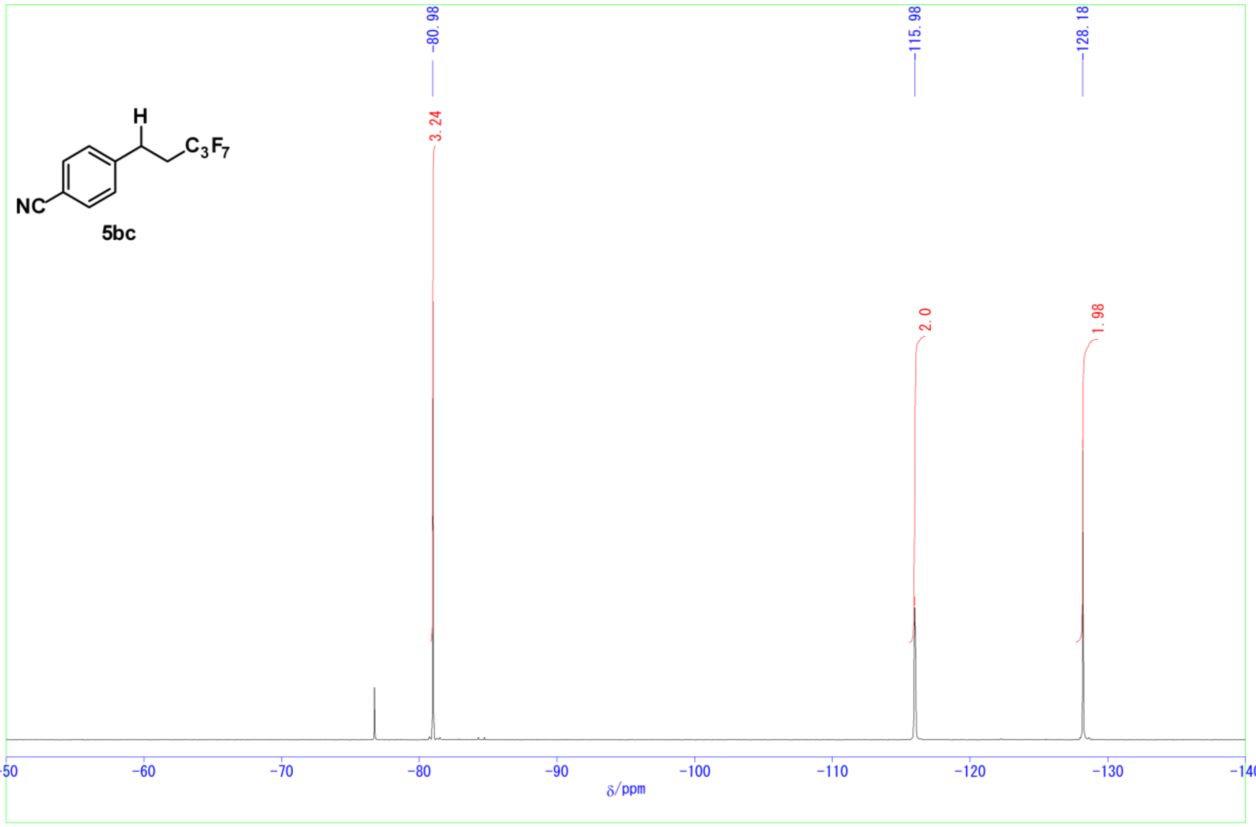

5be: ^{1}H NMR ($CDCl_3$, 400 MHz)

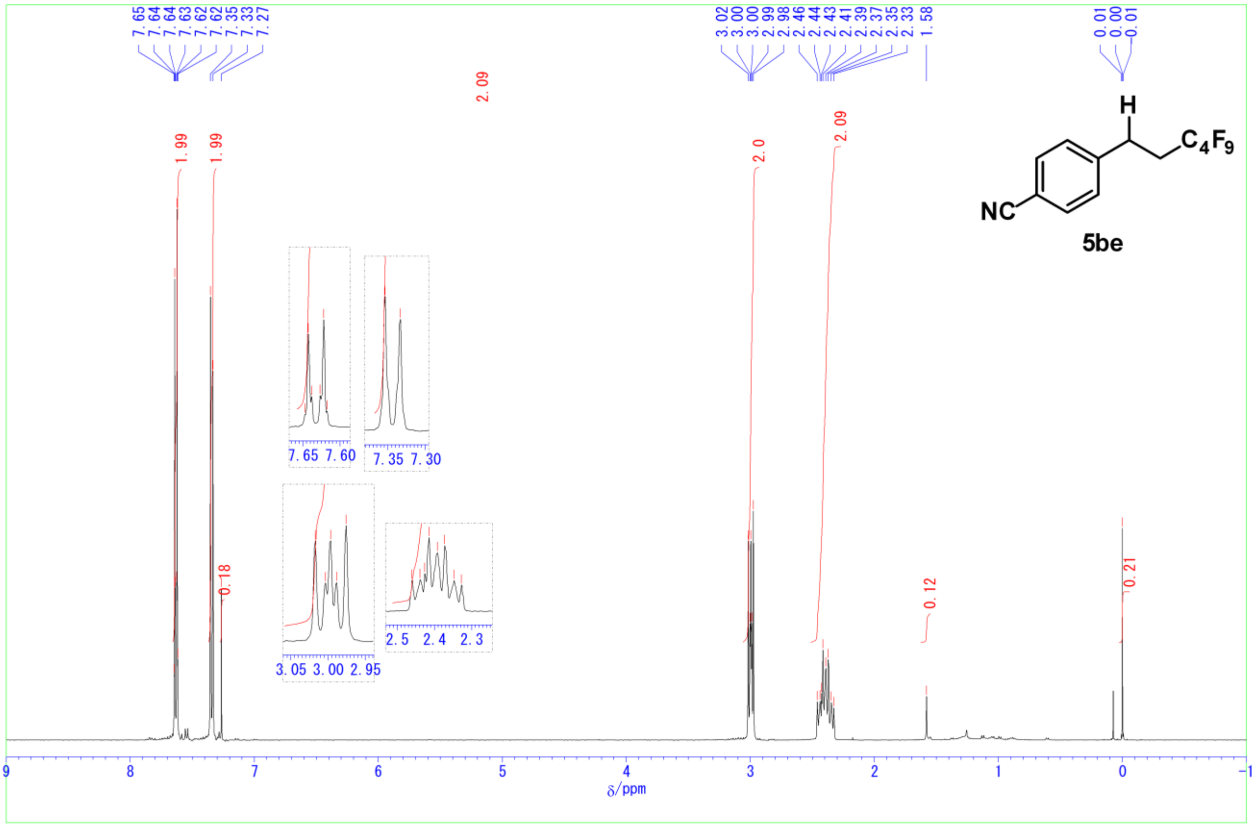

5be: ^{13}C NMR ($CDCl_3$, 151 MHz)

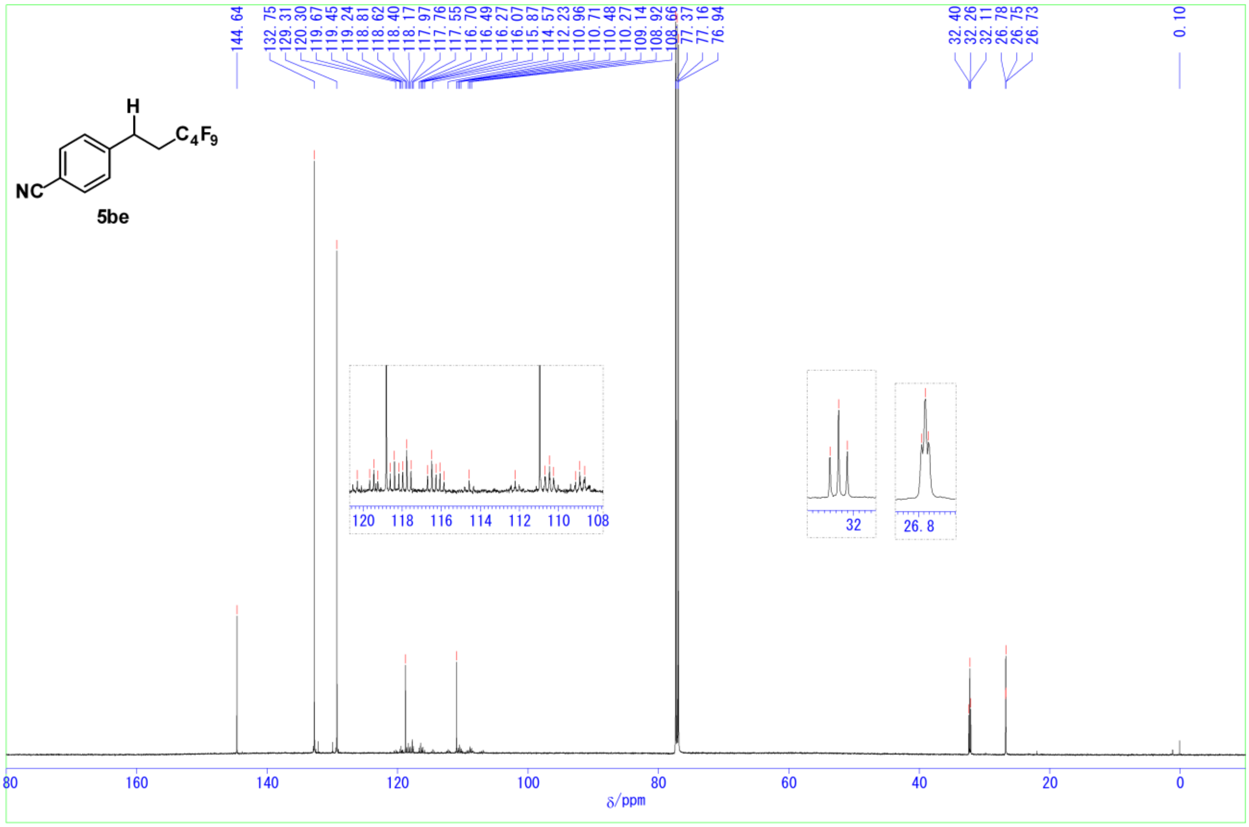

5be: ^{19}F NMR ($CDCl_3$, 376 MHz)

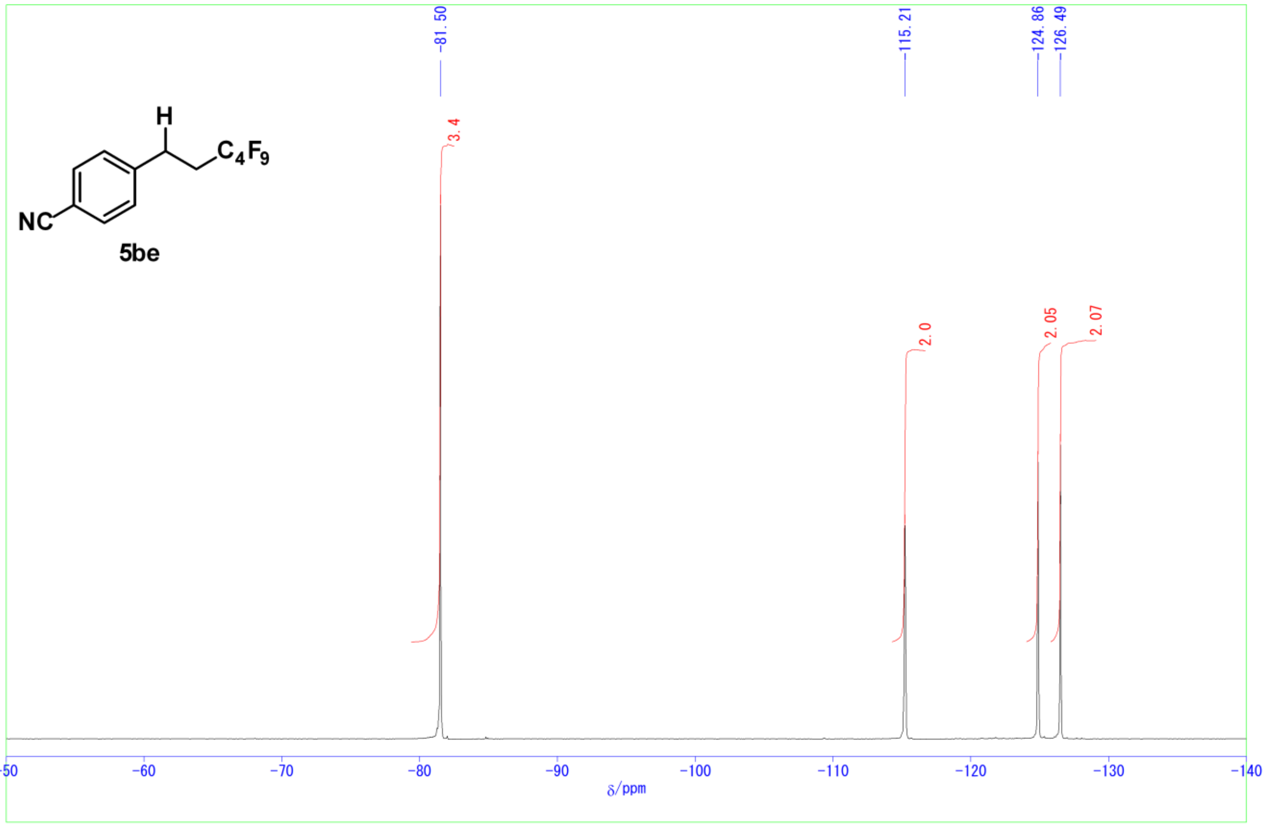

5n: ^{1}H NMR ($CDCl_3$, 500 MHz)

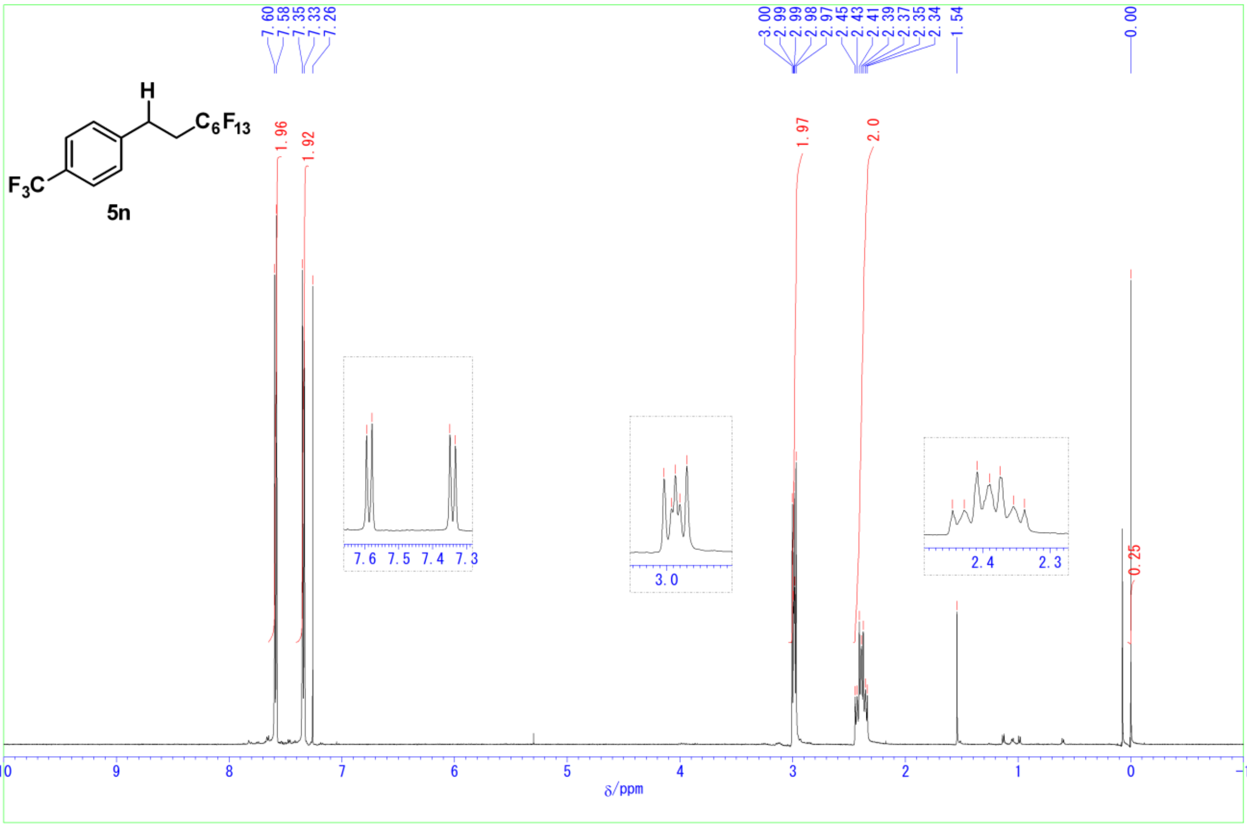

5n: ^{13}C NMR ($CDCl_3$, 151 MHz)

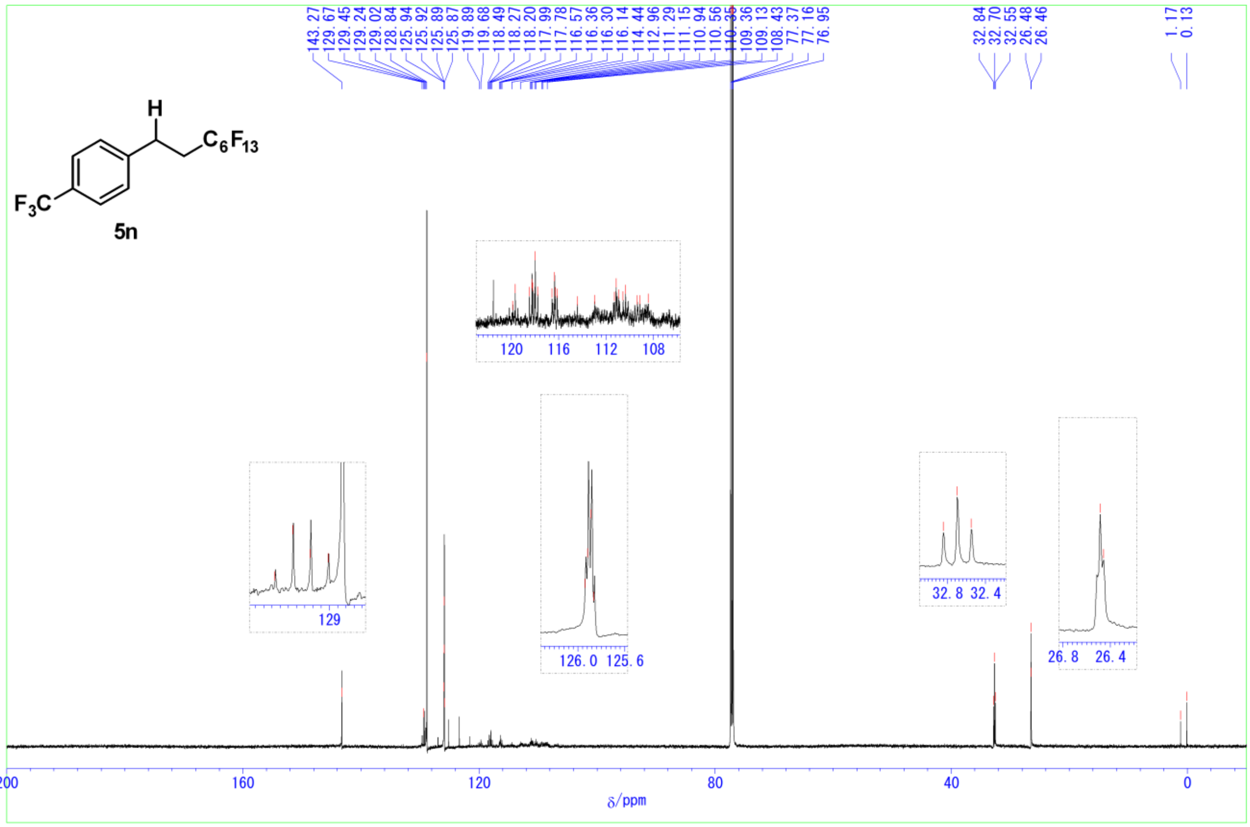

5n: ^{19}F NMR ($CDCl_3$, 471 MHz)

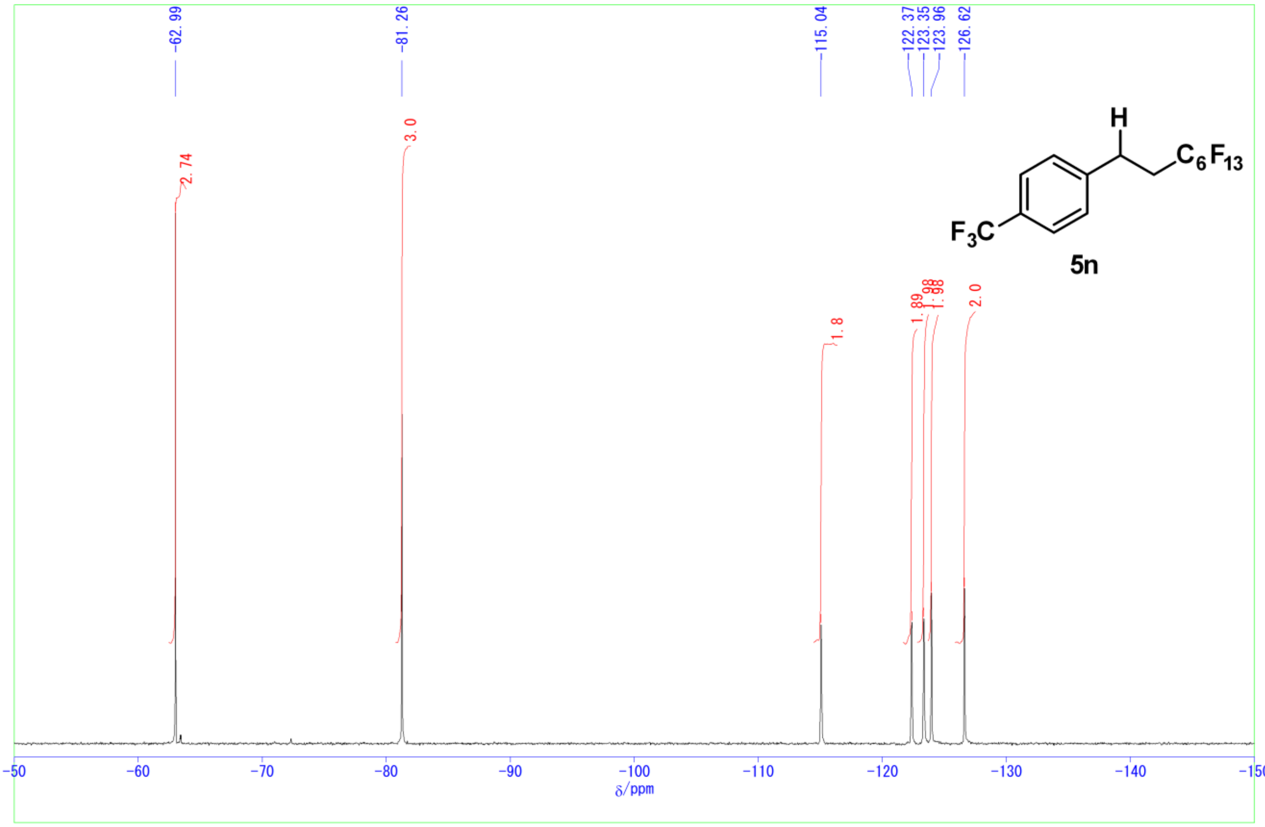

5o: ^{1}H NMR ($CDCl_3$, 500 MHz)

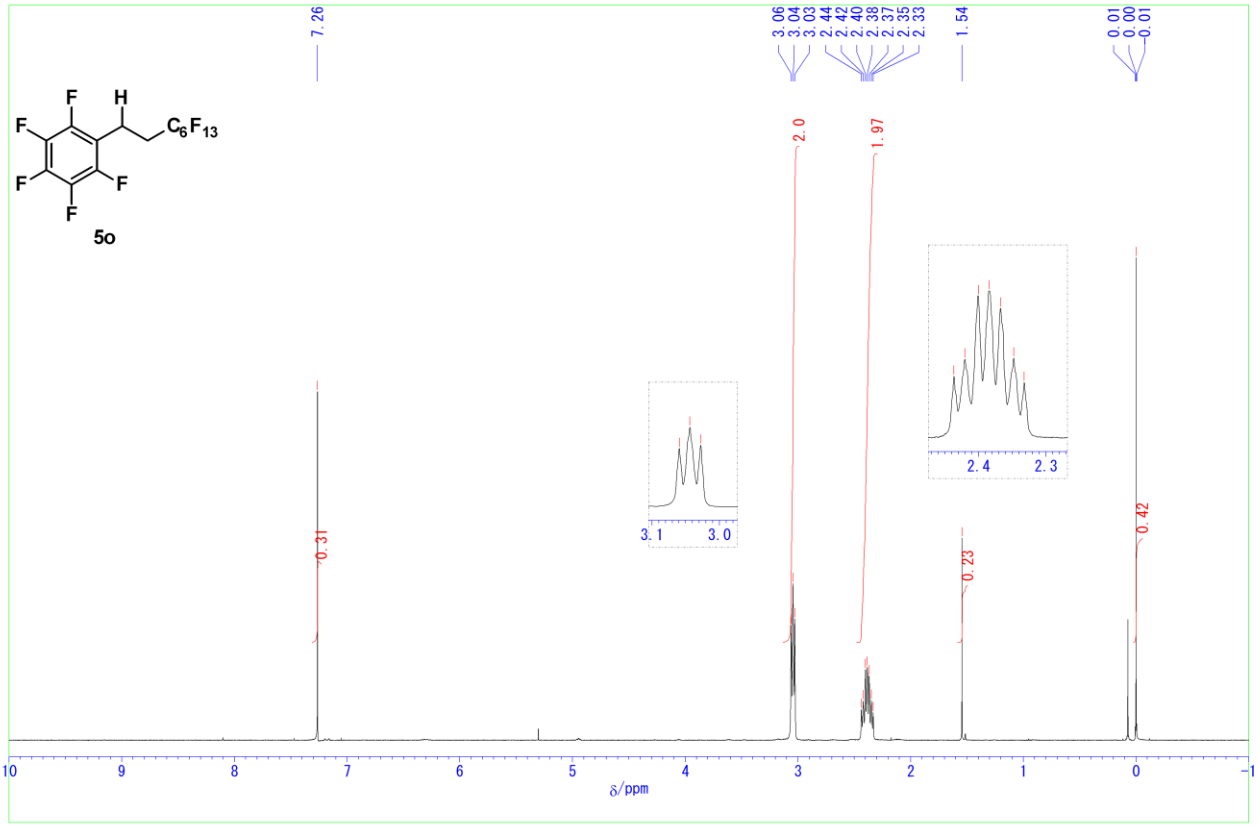

5o: ^{13}C NMR (CDCl$_3$, 151 MHz)

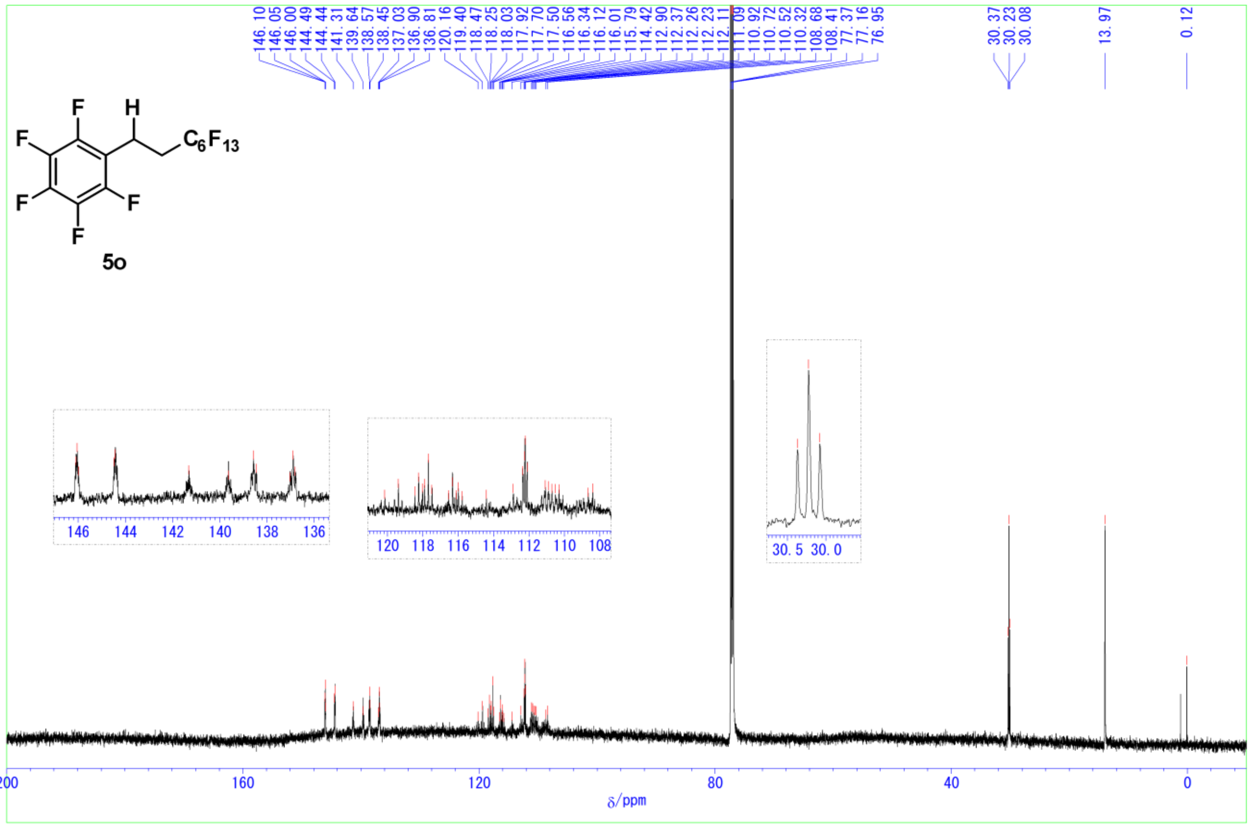

5o: ^{19}F NMR (CDCl$_3$, 471 MHz)

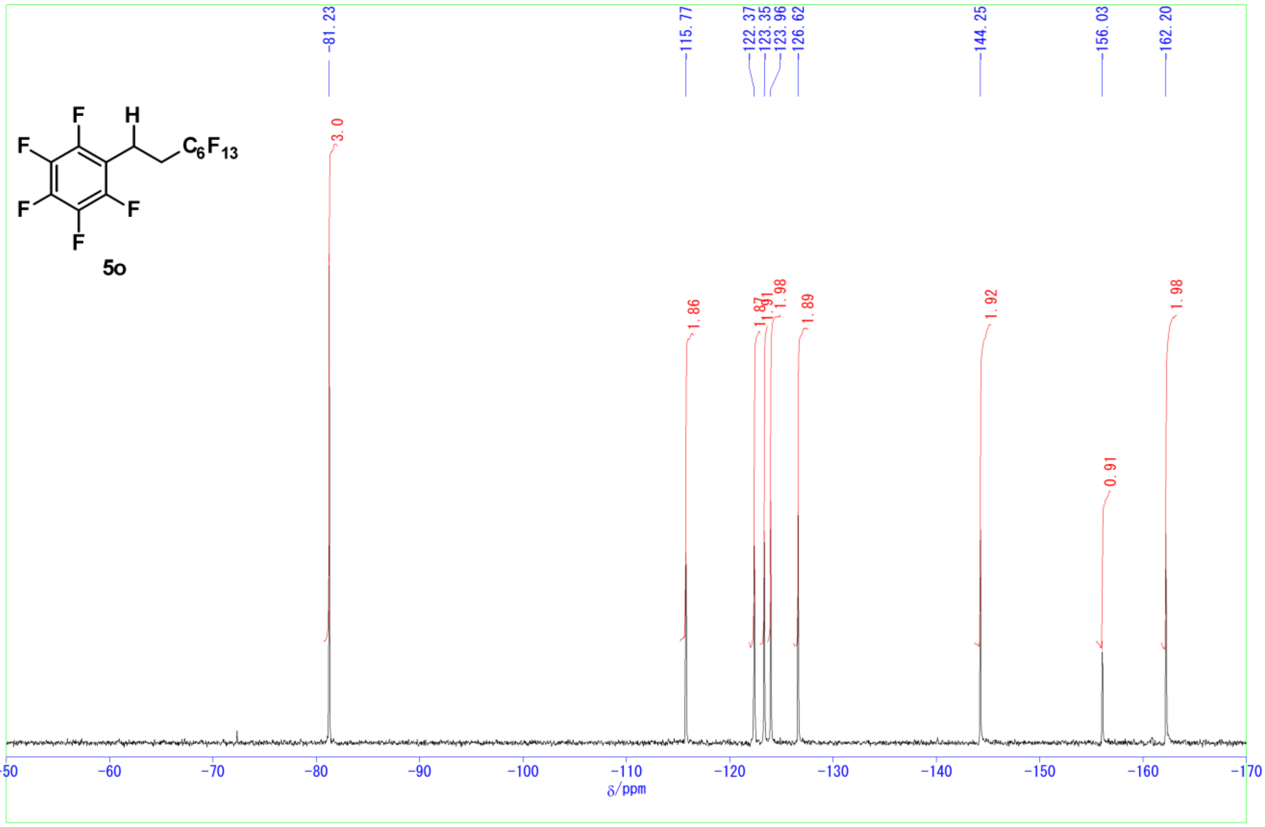

5p: ^{1}H NMR ($CDCl_3$, 400 MHz)

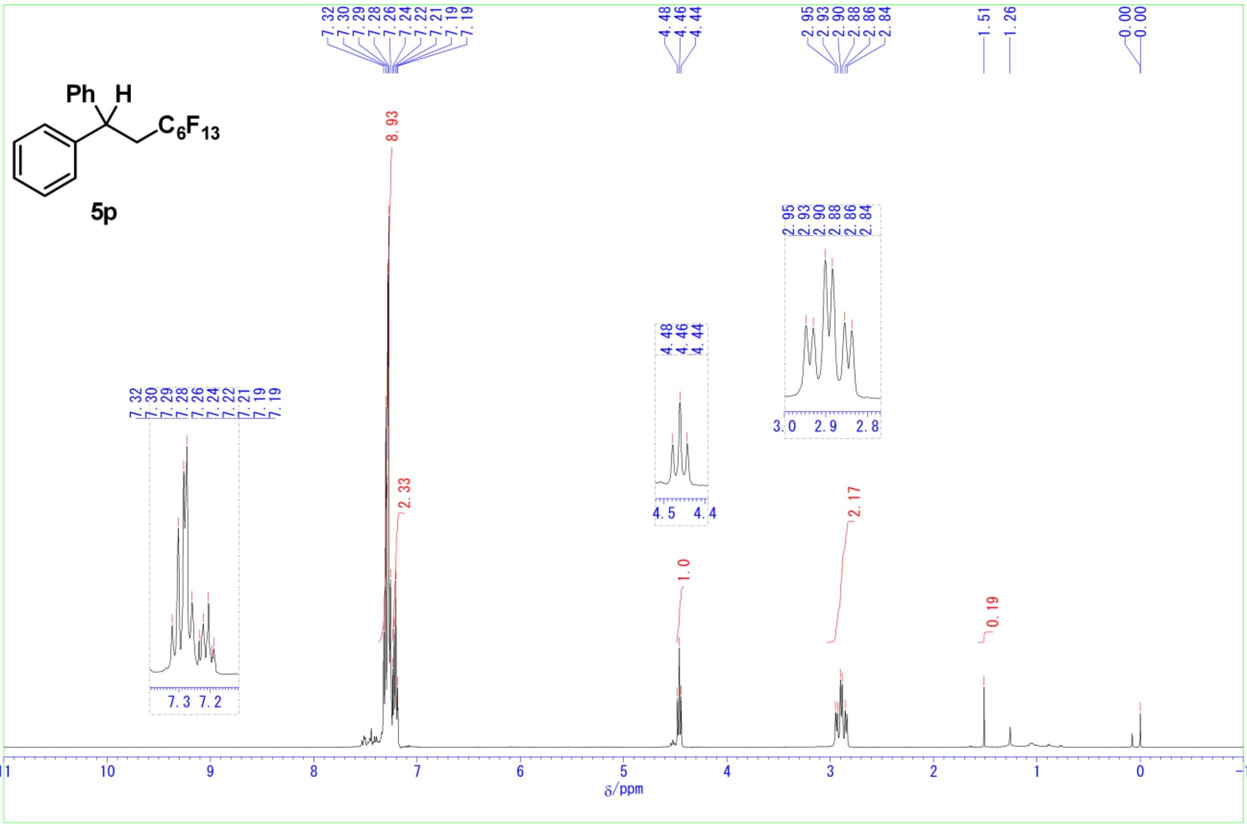

5p: ^{13}C NMR ($CDCl_3$, 151 MHz)

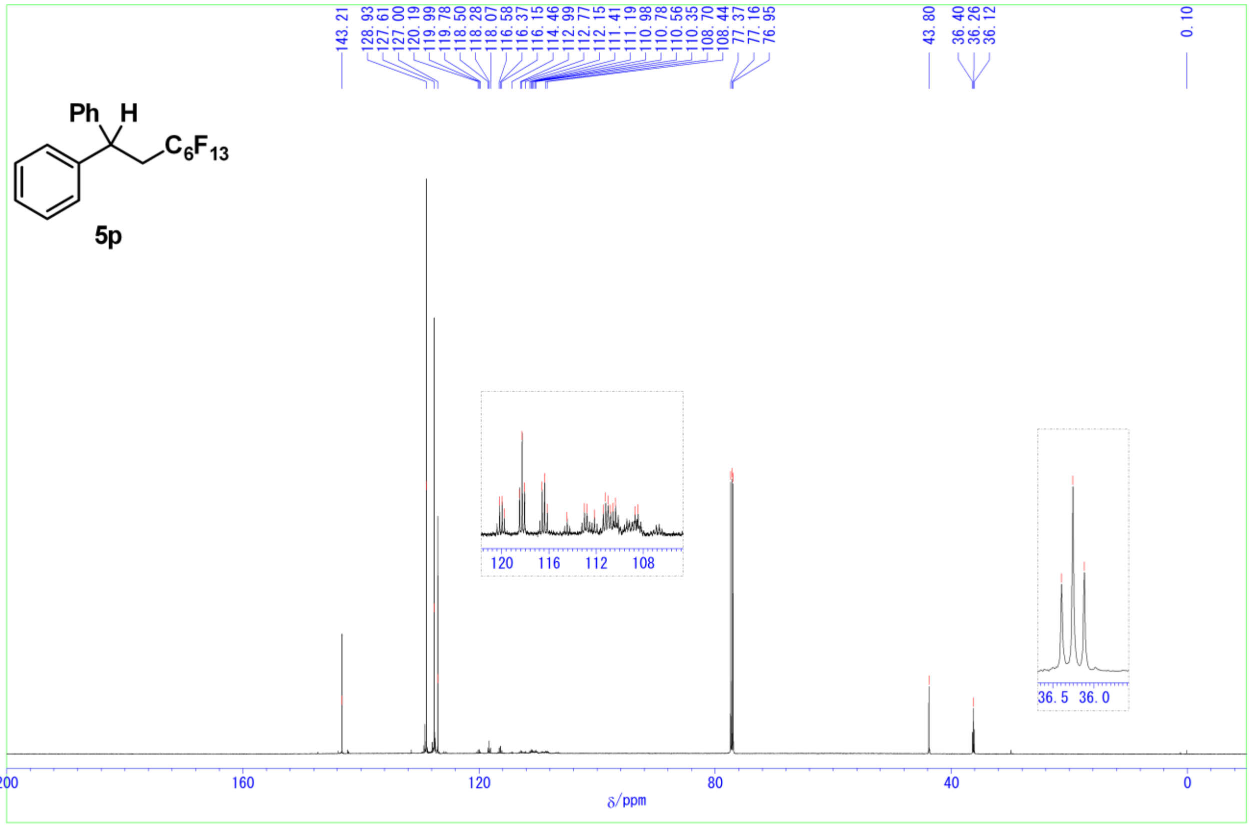

5p: ^{19}F NMR ($CDCl_3$, 376 MHz)

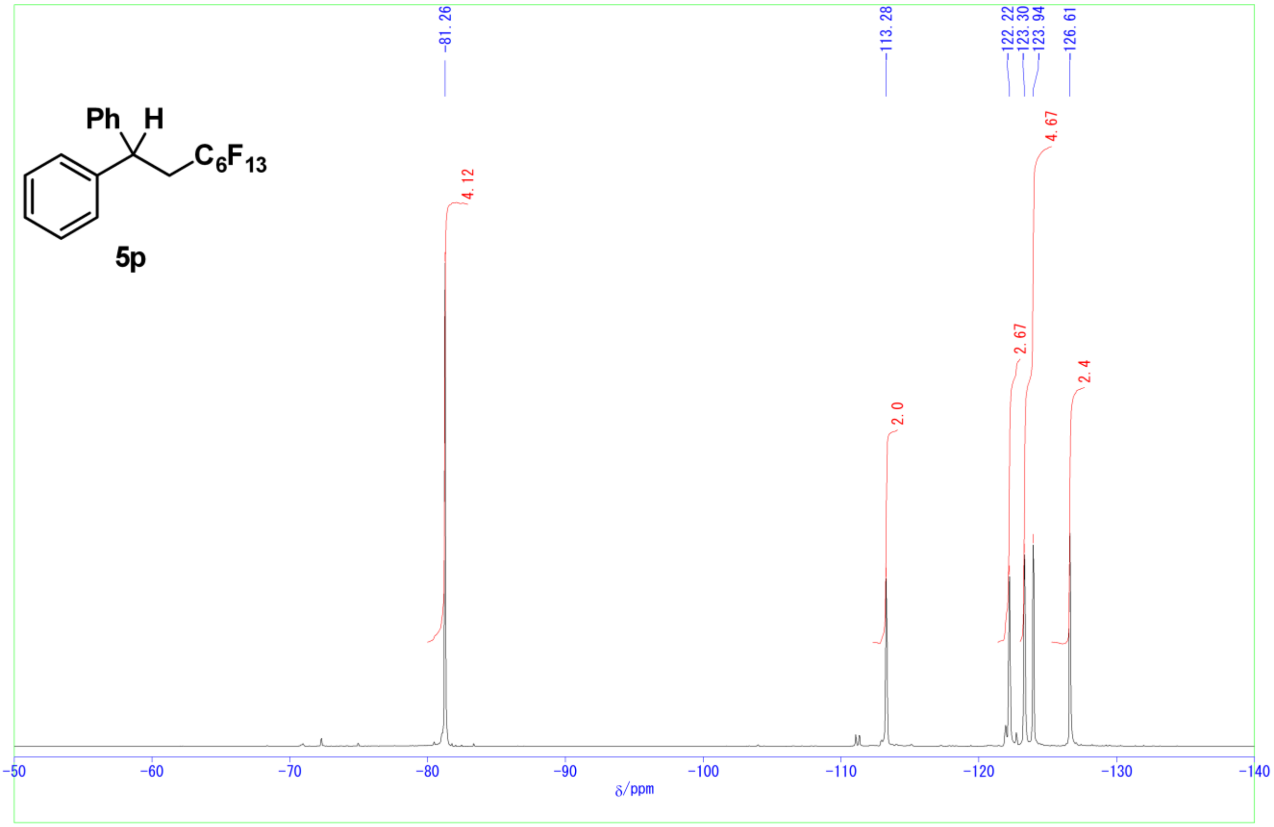

5q: ^{1}H NMR ($CDCl_3$, 400 MHz)

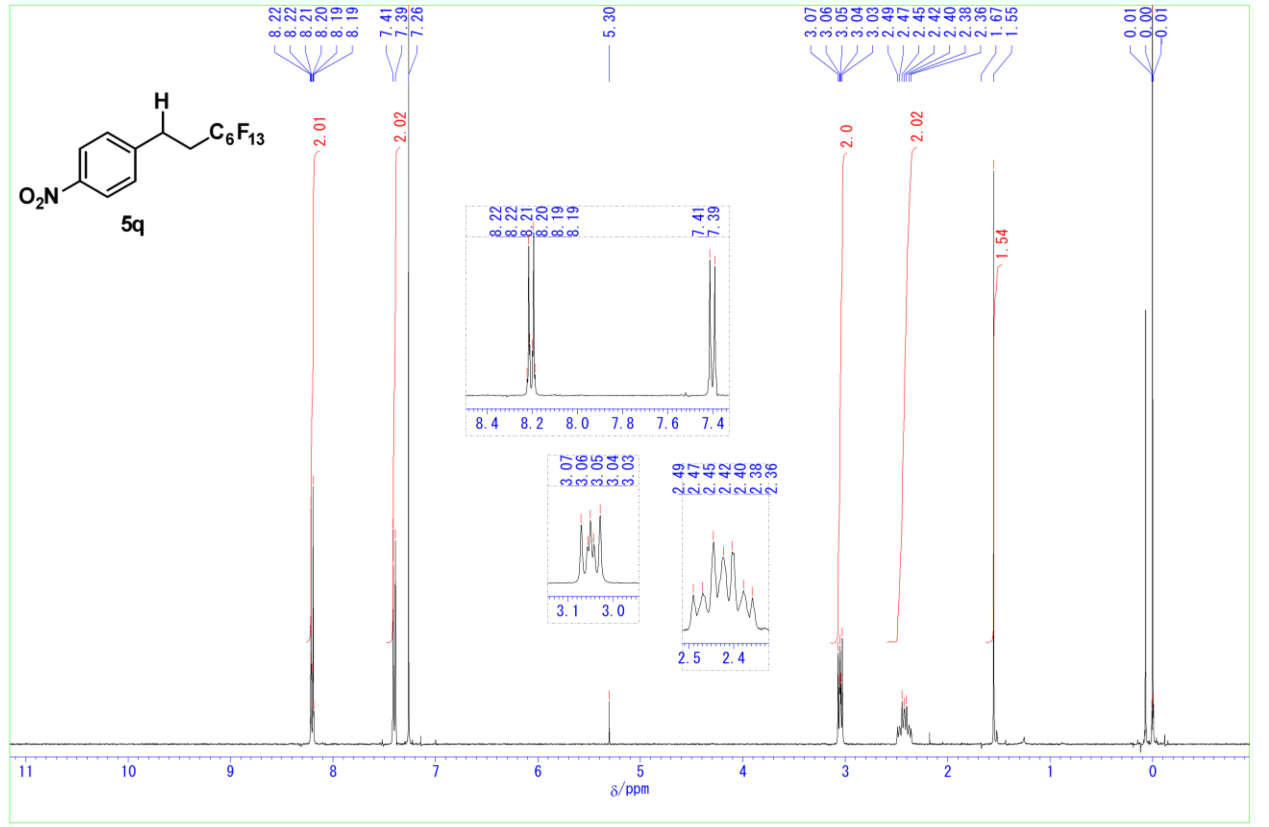

5q: ^{13}C NMR ($CDCl_3$, 151 MHz)

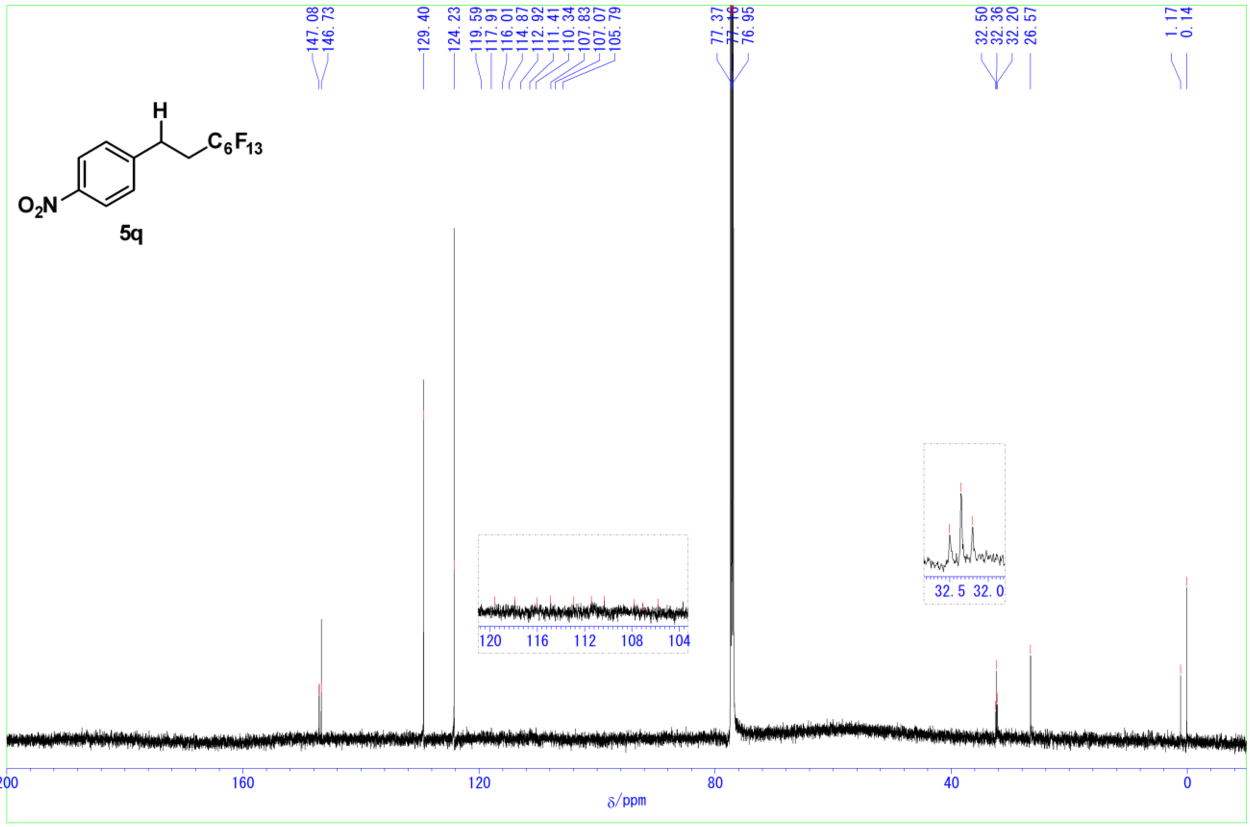

5q: ^{19}F NMR ($CDCl_3$, 376 MHz)

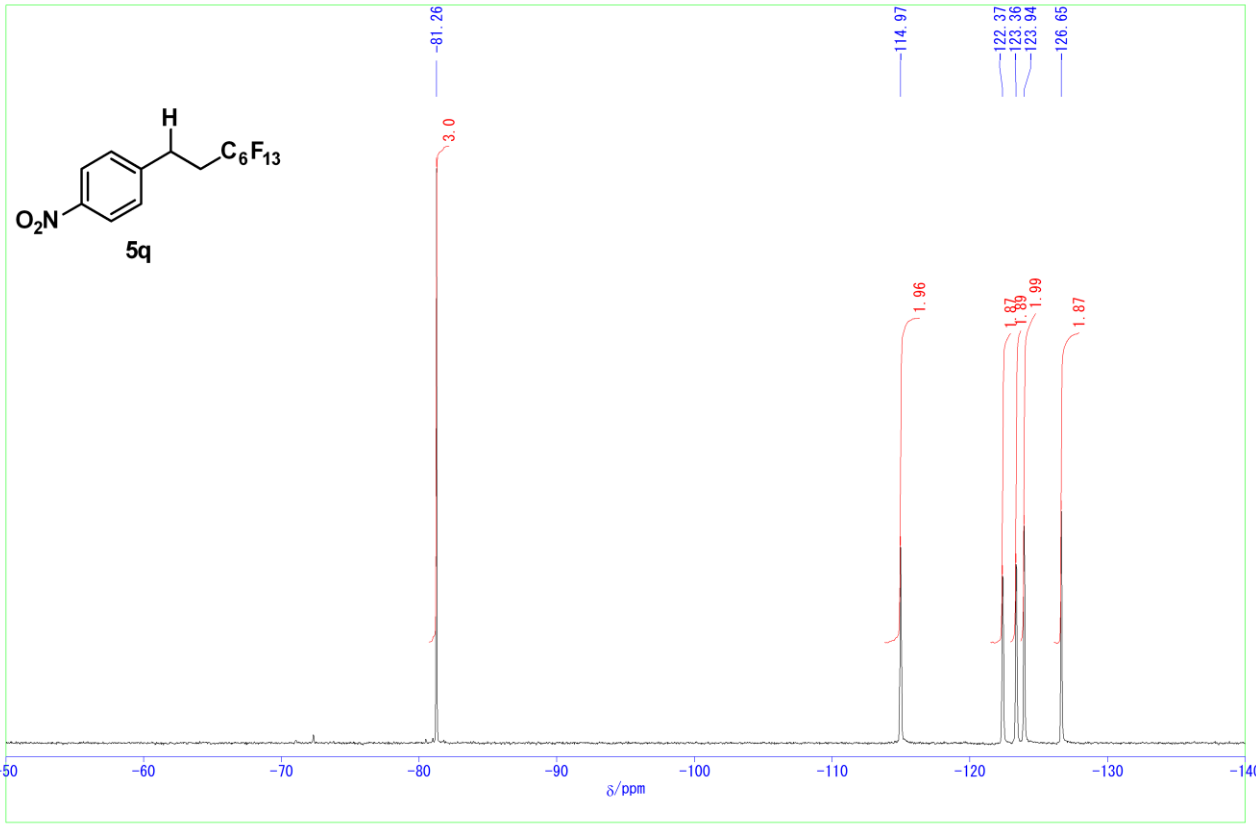

6a: ^{1}H NMR (CDCl$_3$, 400 MHz)

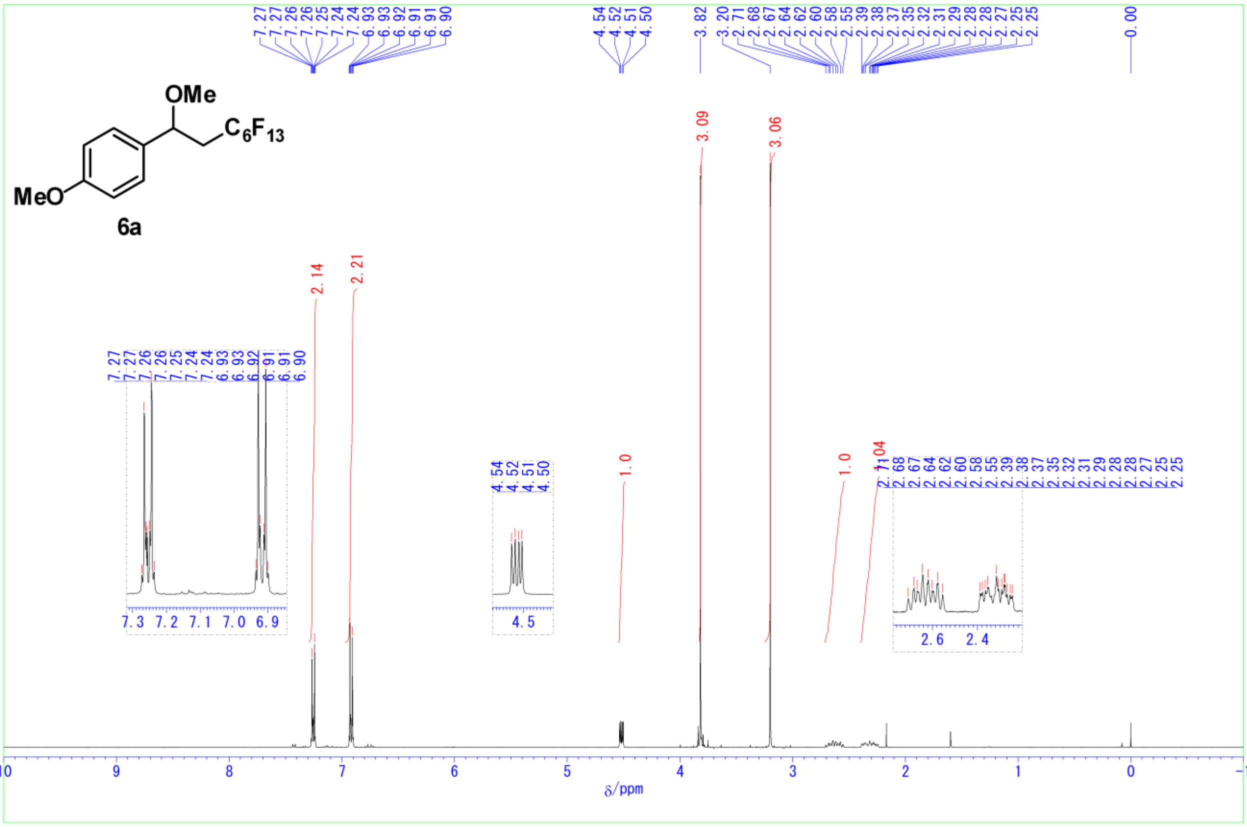

6a: ^{13}C NMR (CDCl$_3$, 151 MHz)

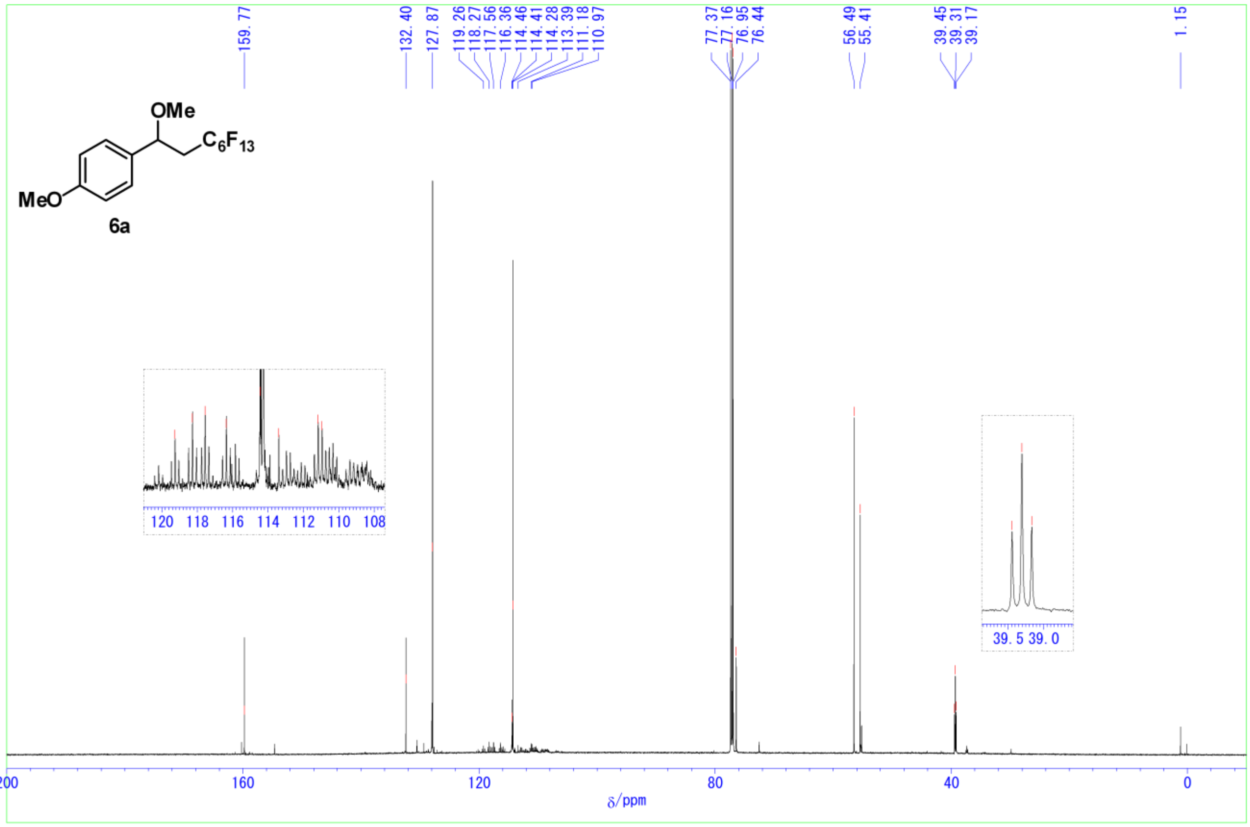

6a: ^{19}F NMR ($CDCl_3$, 376 MHz)

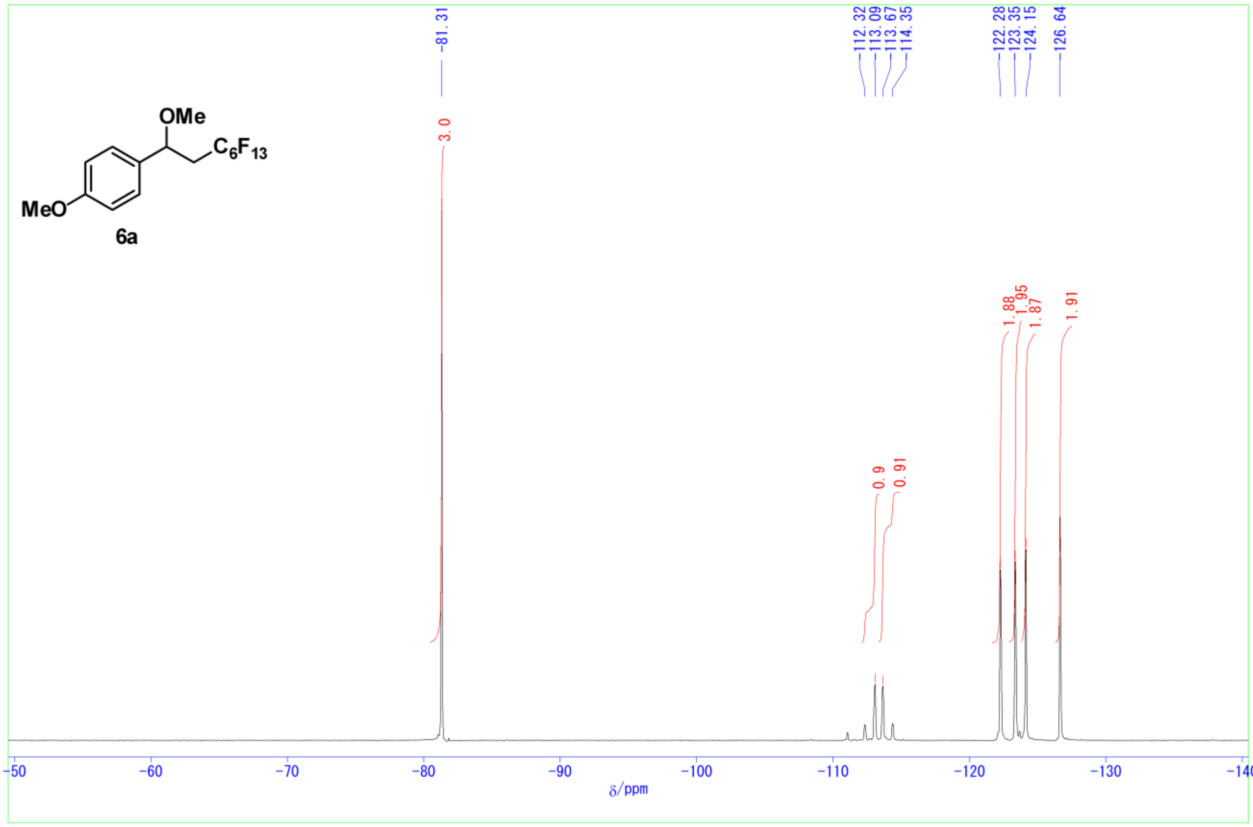

6b: ^{1}H NMR ($CDCl_3$, 400 MHz)

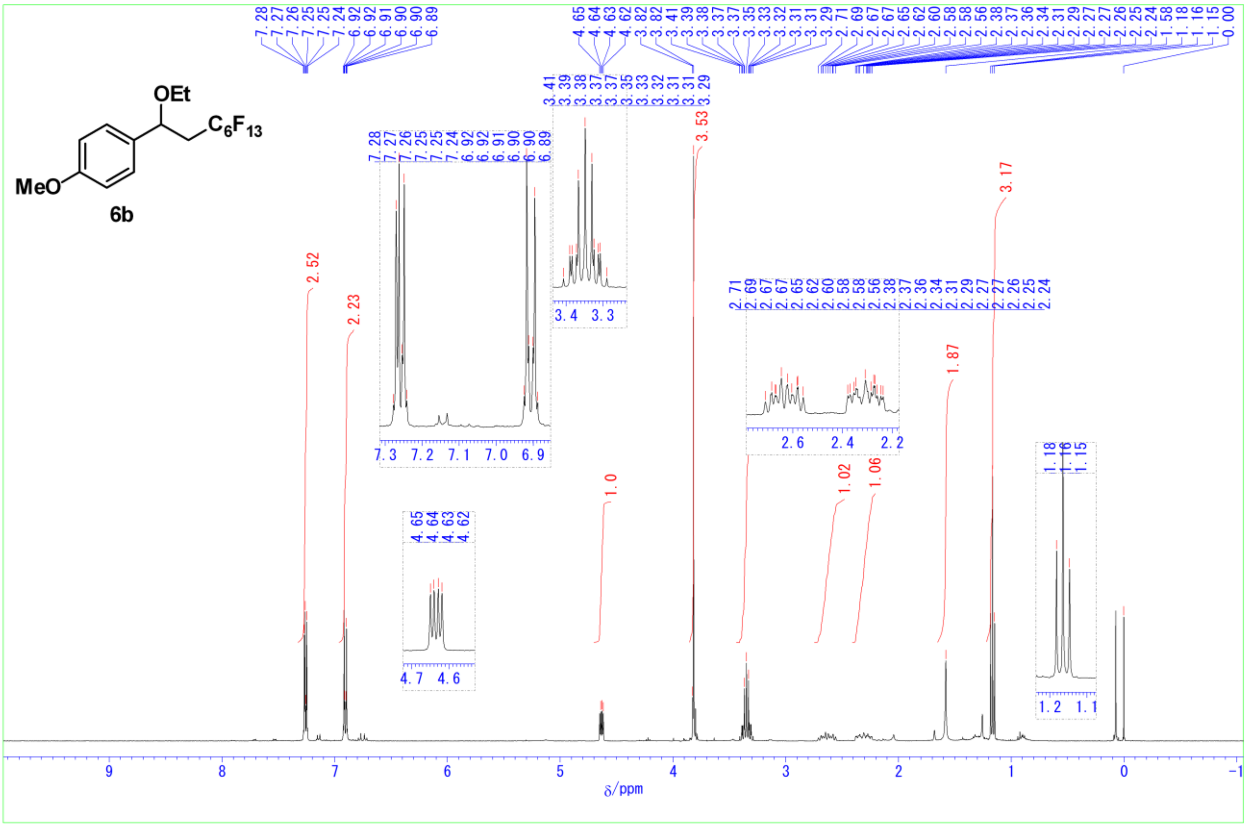

6b: ^{13}C NMR ($CDCl_3$, 151 MHz)

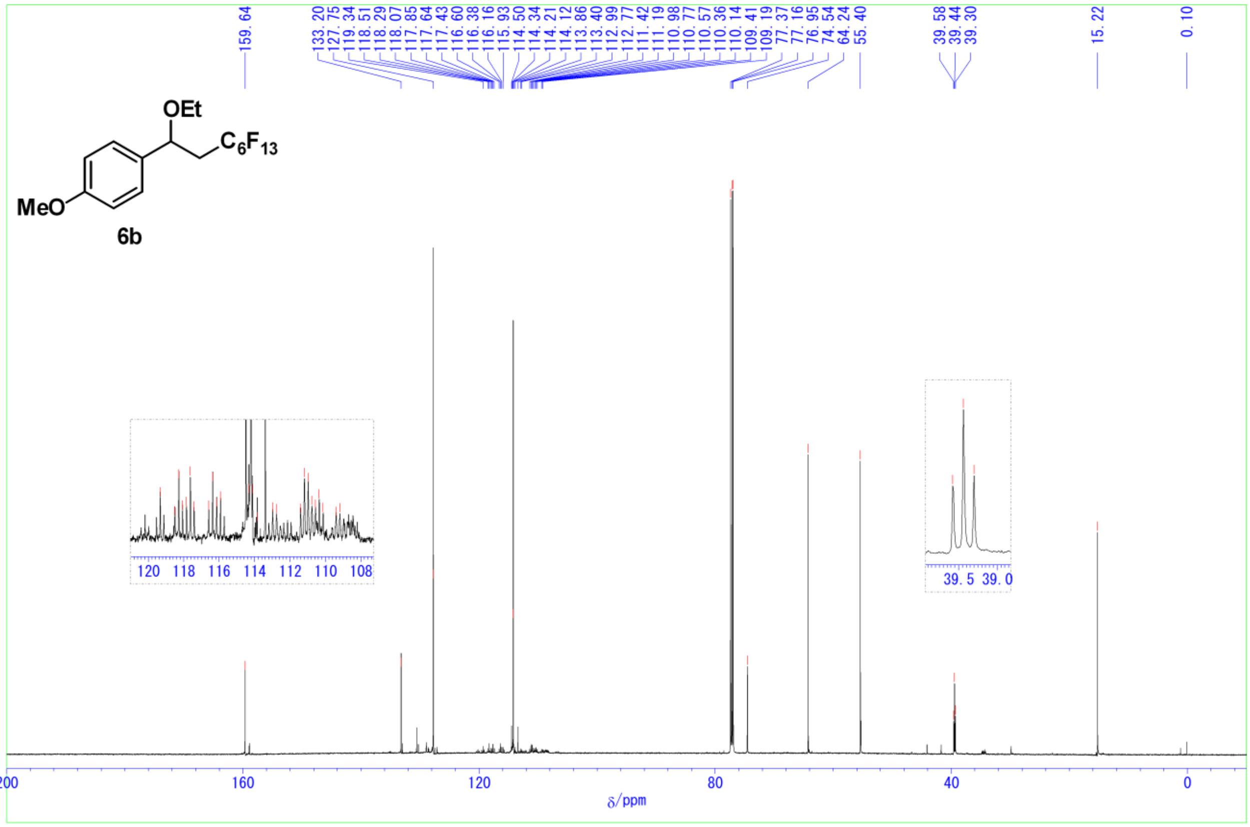

6b: ^{19}F NMR ($CDCl_3$, 376 MHz)

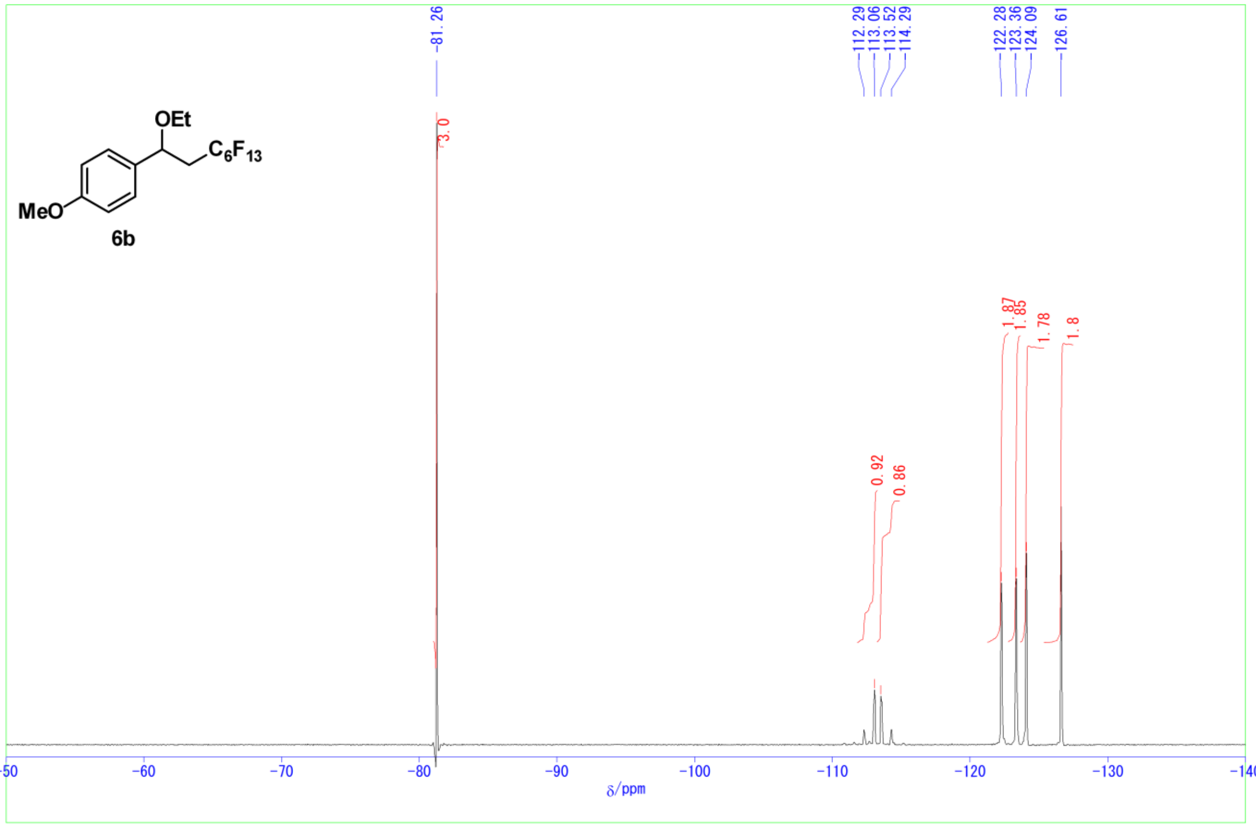

6c: ^{1}H NMR ($CDCl_3$, 500 MHz)

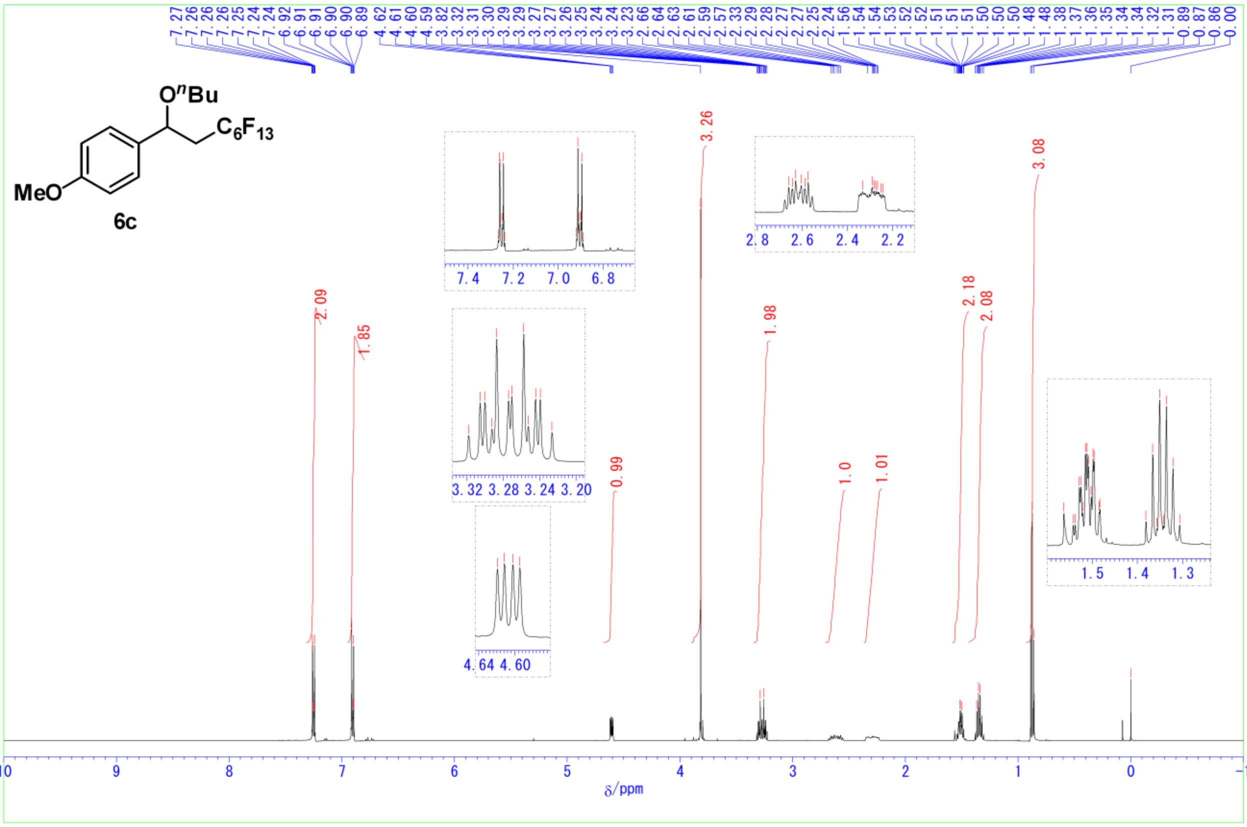

6c: ^{13}C NMR ($CDCl_3$, 151 MHz)

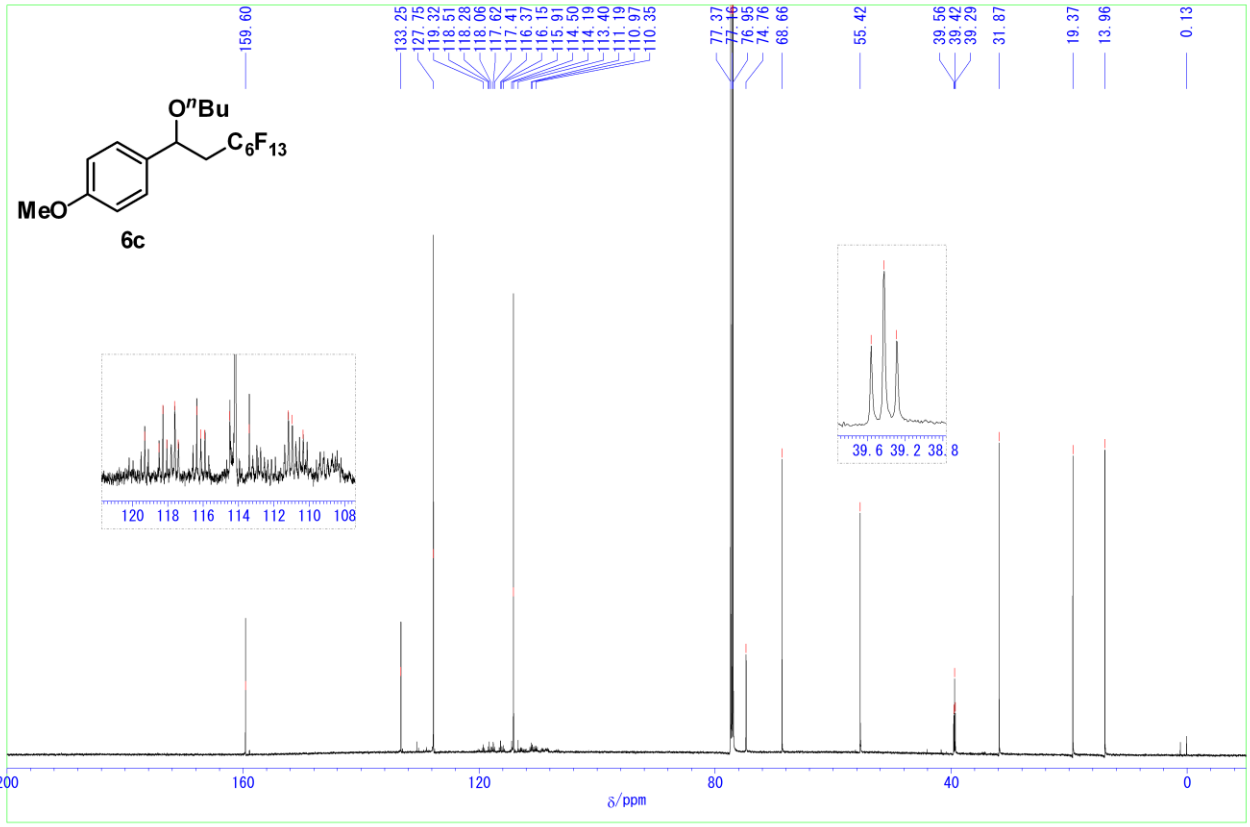

6c: ^{19}F NMR (CDCl$_3$, 471 MHz)

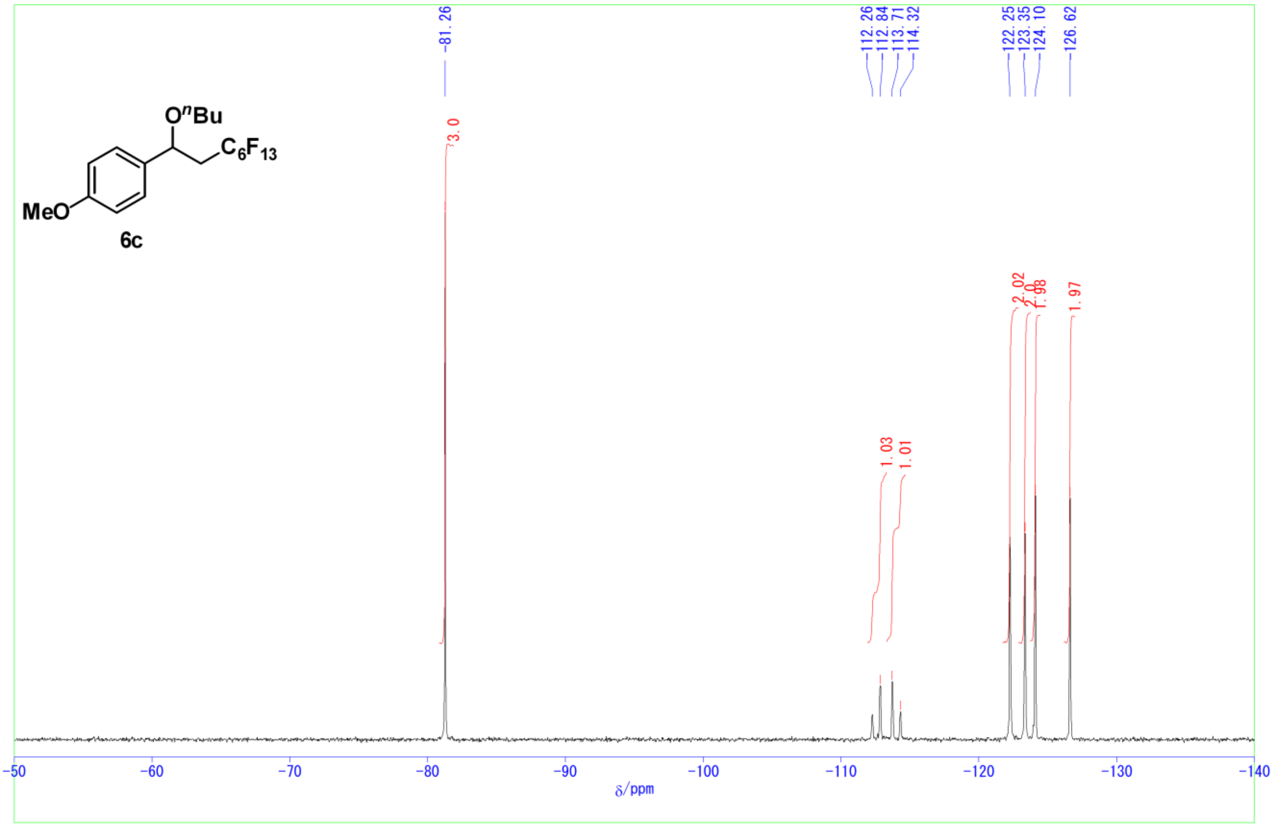

8: ^{1}H NMR (CDCl$_3$, 500 MHz, contains **9**)

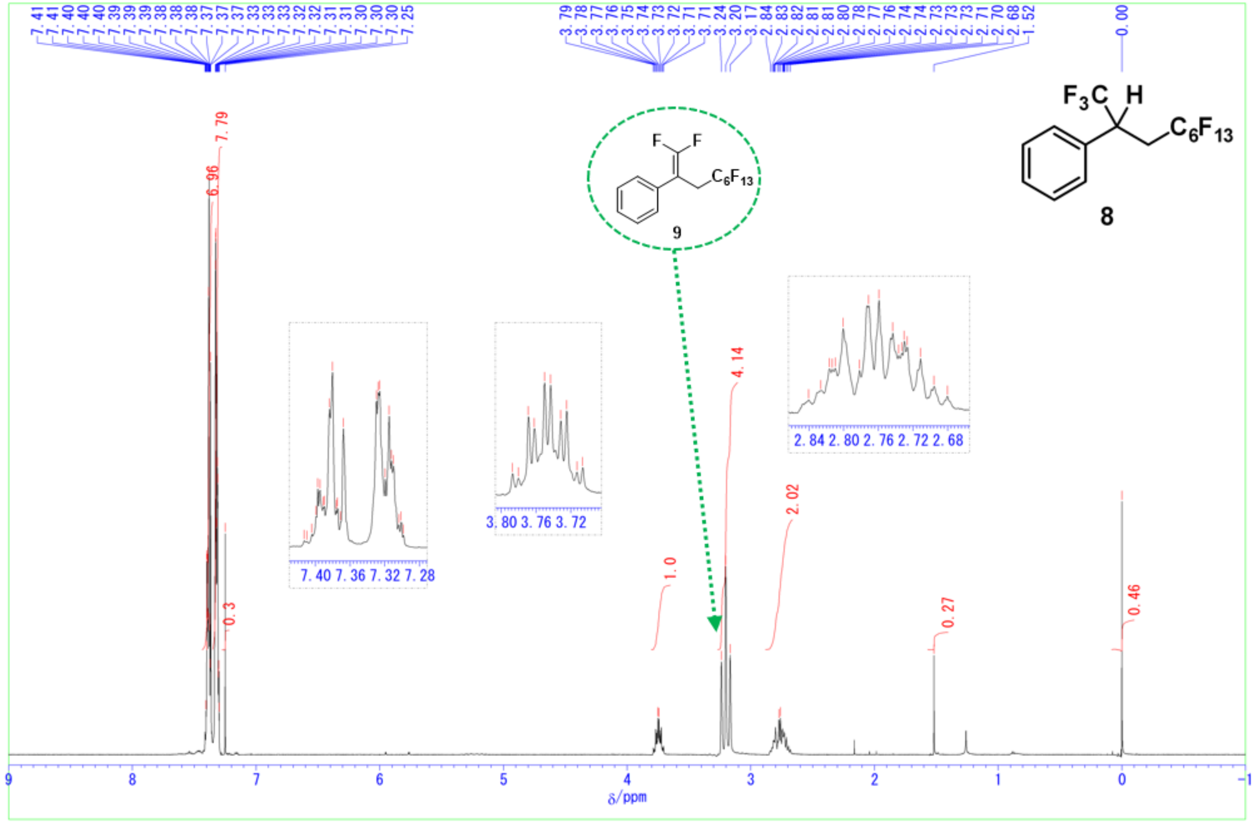

8: ^{13}C NMR ($CDCl_3$, 151 MHz contains **9**)

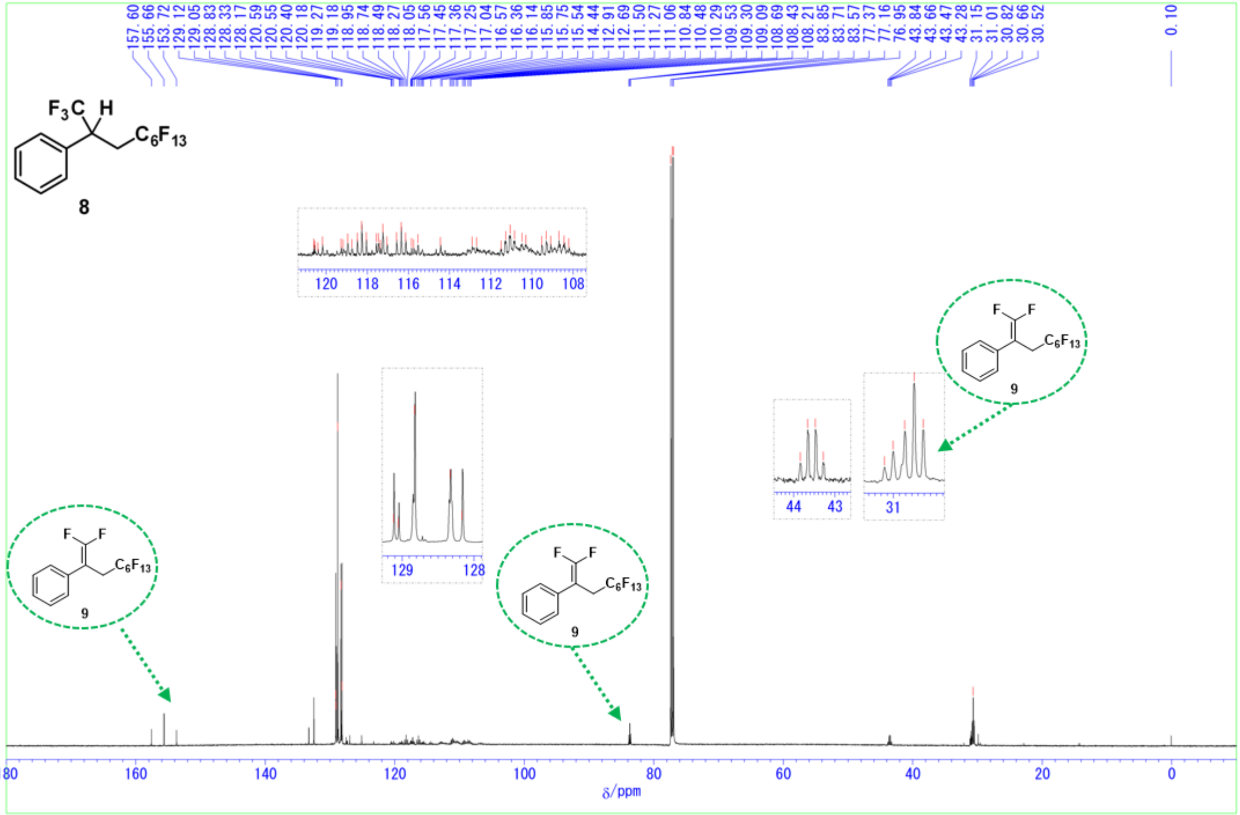

8: ^{19}F NMR ($CDCl_3$, 471 MHz, contains **9**)

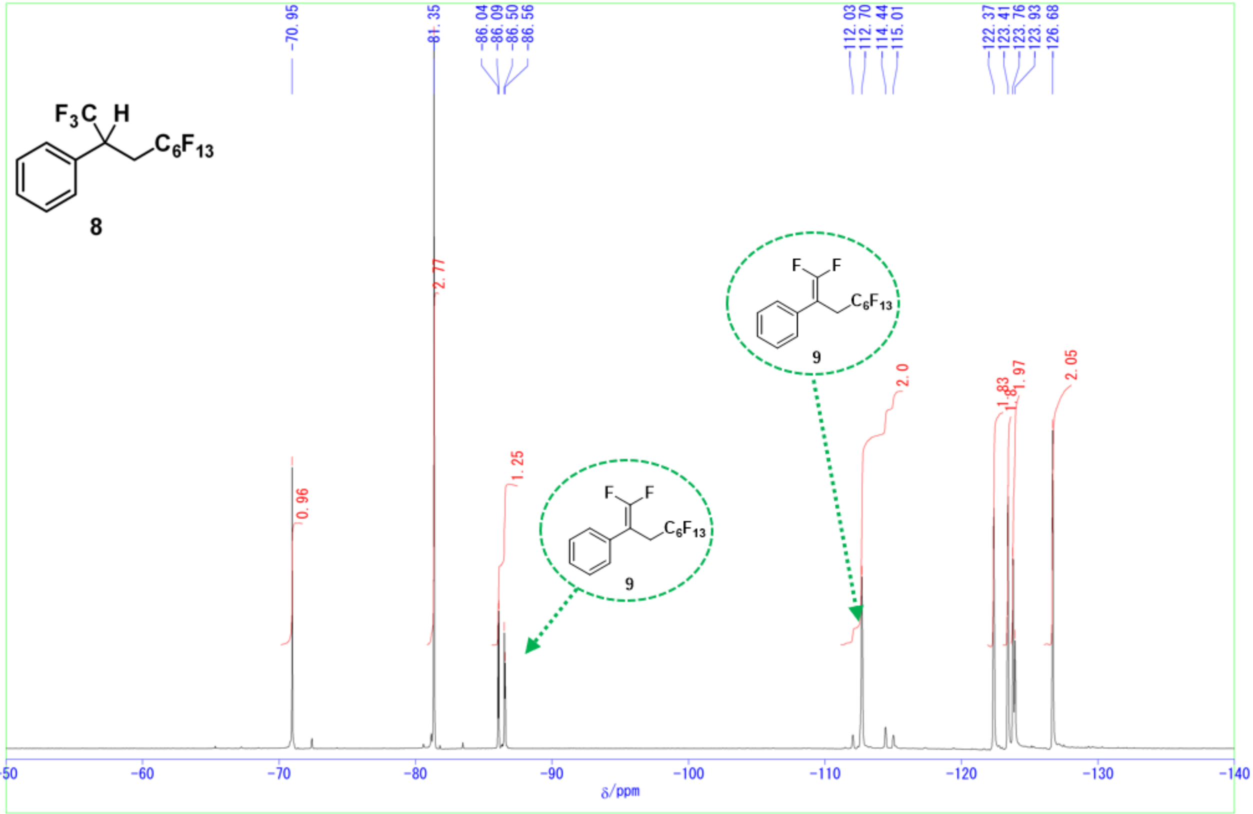

9: ^{1}H NMR ($CDCl_3$, 500 MHz)

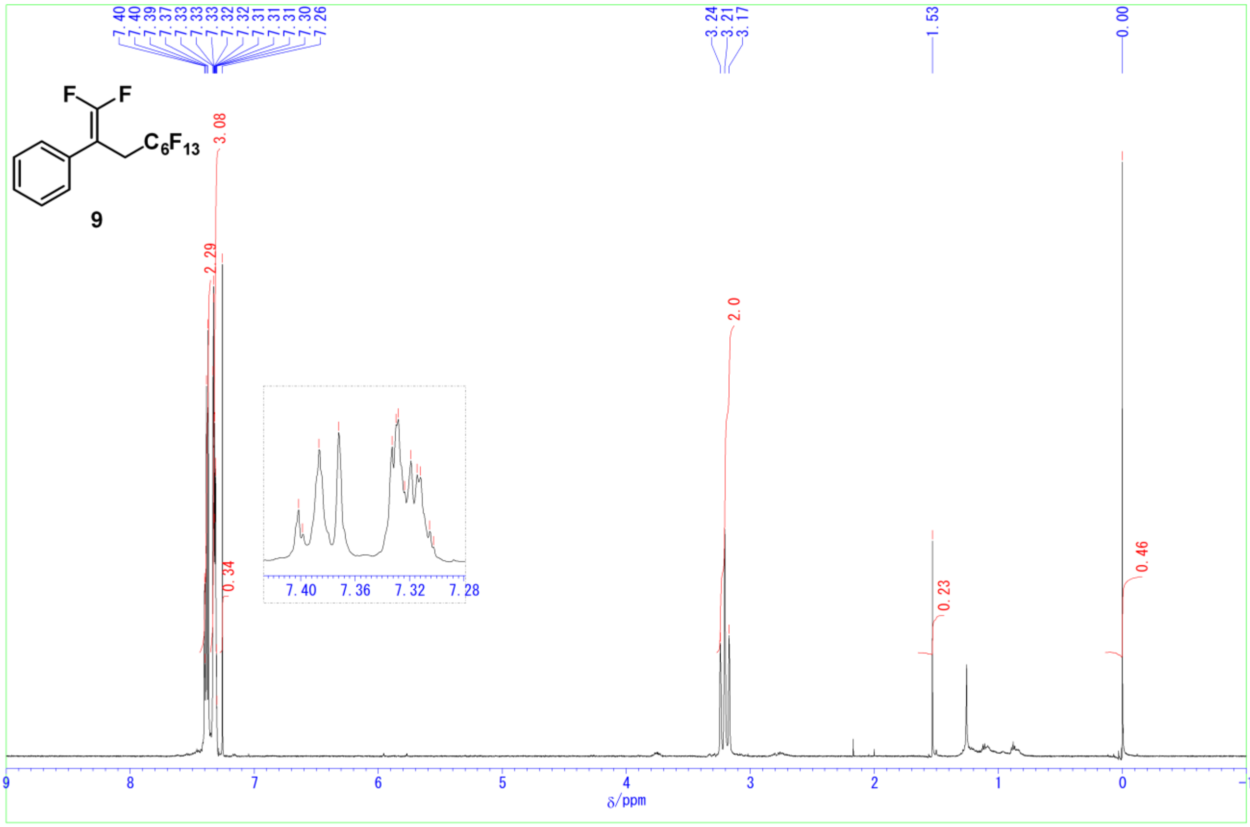

9: ^{13}C NMR ($CDCl_3$, 151 MHz)

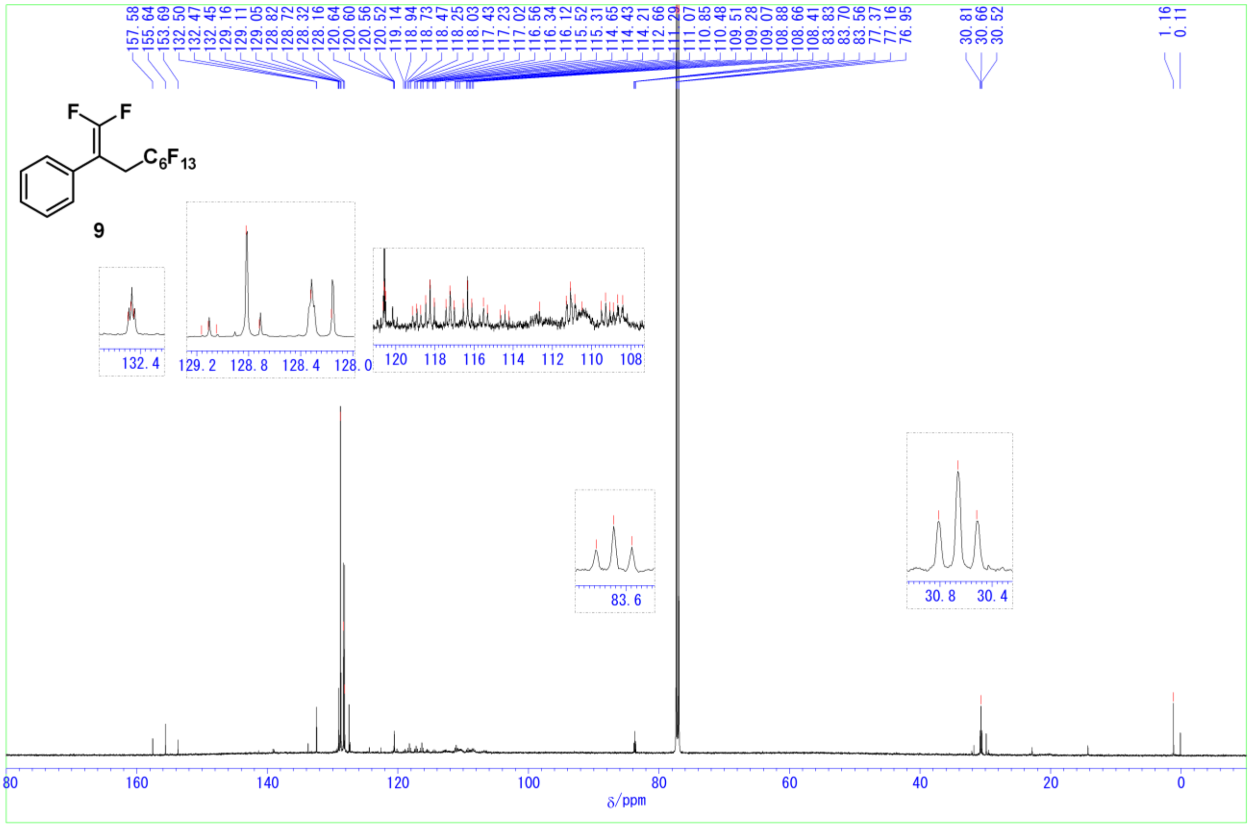

9: ^{19}F NMR (CDCl$_3$, 471 MHz)

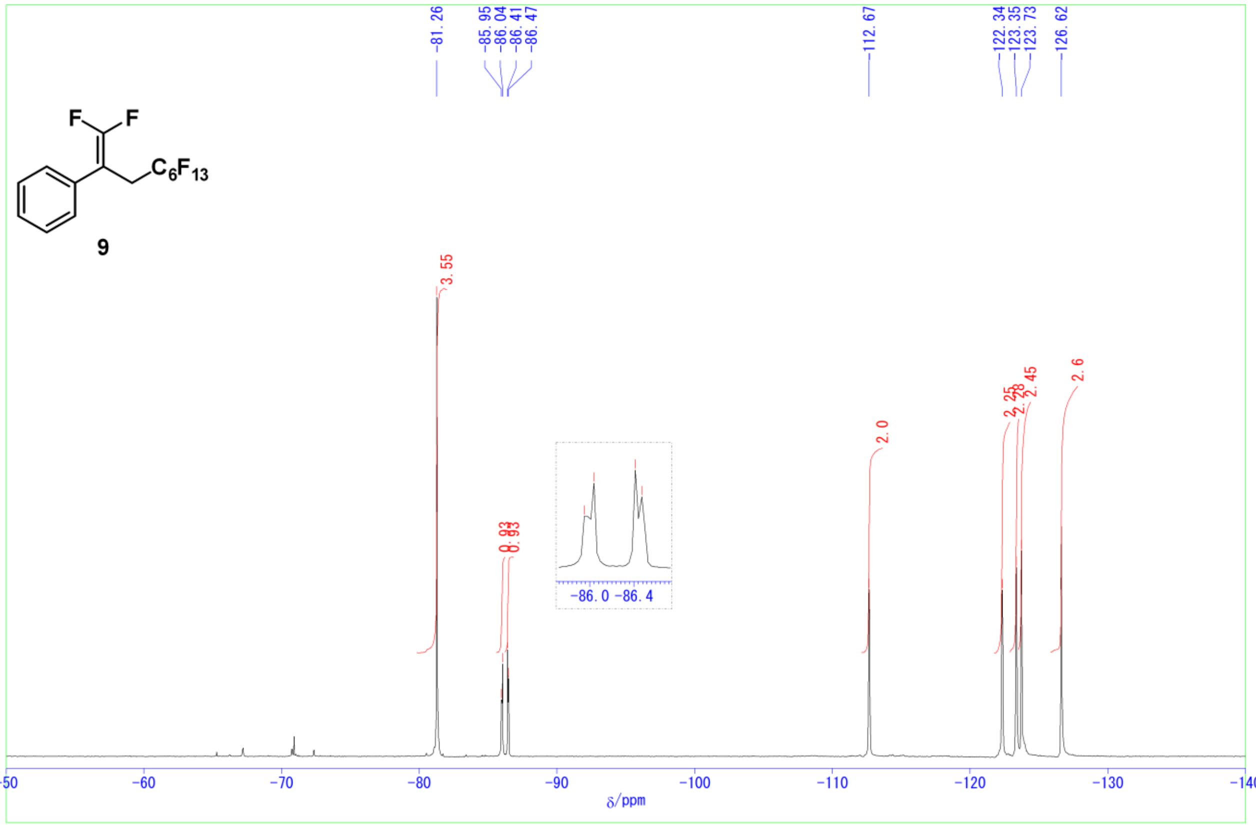

Supplement: Supplementary file 1 [file molecules-28-07577-s001.zip › molecules-2703371-supplementary.pdf]
